# A Unifying Bioinspired Synthesis of (−)-Asperaculin A and (−)-Penifulvin D

**DOI:** 10.1021/acs.orglett.1c00955

**Published:** 2021-04-08

**Authors:** Ian R. George, Miguel López-Tena, Anders P. Sundin, Daniel Strand

**Affiliations:** Centre for Analysis and Synthesis, Department of Chemistry, Lund University, Box 124, 221 00 Lund, Sweden

## Abstract

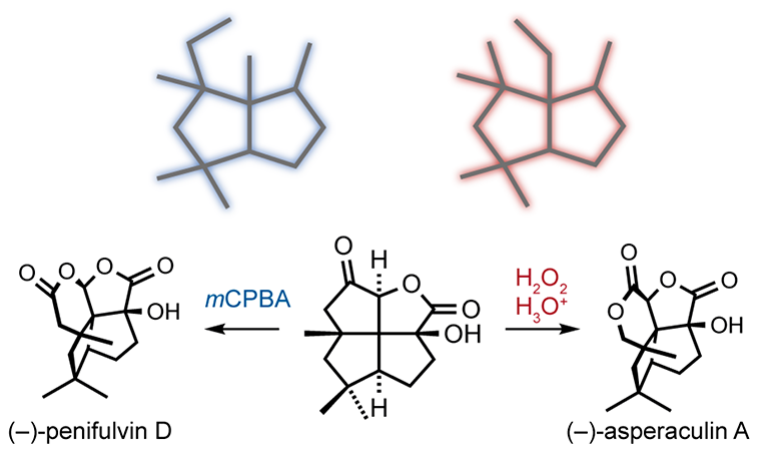

The first syntheses of the isomeric dioxafenestrene natural products (−)-asperaculin A and (−)-penifulvin D are reported. Each target is formed selectively by choice of oxidant in a final divergent bioinspired Baeyer–Villiger (BV) reaction. Density functional theory calculations reveal that electrostatic interactions between the oxidant leaving group and the lactone motif accounts for a reversal of selectivity with H_2_O_2_/H_3_O^+^ compared to peracids. Synthetic features include forging the polycyclic carbon framework with a diastereoselective *meta*-photocycloaddition biased by an ether substituent at the aryl α-position. The encumbered tertiary alcohol was installed by cyanation of a ketone intermediate followed by nonaqueous hydrolysis of the resulting delicate cyanohydrin.

Biosynthesis remains a rich source of inspiration for discovering new strategies and tactics in chemical synthesis.^1,2^ Guided by this view, we were drawn to the isomeric triquinanes asperaculin A (**3**)^3^ and penifulvin D (**2**)^4^ (Scheme 1). These daunting structures present three consecutive quaternary stereogenic centers flanked by two tertiary centers across the heart of a tetracyclic dioxa[5.5.5.6]fenestrene motif.^5,6^ The penifulvins exhibit potent insecticidal properties, but mechanistic details are still unknown. An elegant study by Zou revealed that the penifulvins originate biosynthetically from a silphinene core via an enzyme-mediated Baeyer–Villiger (BV) pathway.^7^ During the preparation of this manuscript, the same lab extended this mechanism to aspergilanes.^8^ We similarly considered the possibility that asperaculin A and penifulvin D share a biosynthetic origin via a BV reaction, but also, that chemical synthesis of both structures through a divergent biomimetic BV reaction was a feasible prospect. Here, we report the first chemical synthesis of a member of the aspergilanes, asperaculin A, through the postulated BV approach. We also show that a simple change of oxidant reverses the selectivity in favor of the isomeric penifulvin D, a first chemical synthesis of a ring-hydroxylated penifulvin. Reagent and substrate influence on regioselectivity in BV reactions has been studied,^9−14^ but to our knowledge, such levels of reversal of selectivity by choice of simple reagents have not been described. The study aligns with the revised biosynthetic relationship between aspergilanes and penifulvins^3,8^ and offers new mechanistic insight into regioselective BV reactions. The concise approach to both targets, and by extension to their designed congeners, enables access to new substrates for enzymatic-modification and mechanistic studies.

**Scheme 1:**
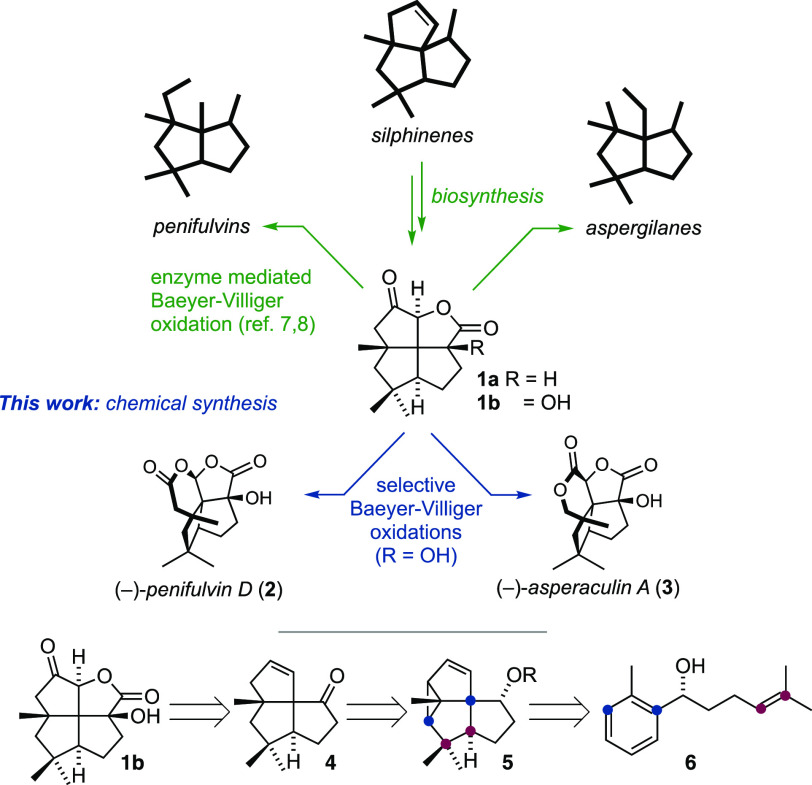
Biosynthesis and Chemical Syntheses of Penifulvins and Aspergilanes from a Silphinene Core (Top); Retrosynthetic Analysis (Bottom)

A synthetic connection between the silphinenes and the penifulvins was realized in pioneering studies by Mülzer and Gaich. Concise asymmetric syntheses of penifulvins A–C^15−17^ were accomplished through *meta*-photocycloadditions to assemble the silphinene cores. Wender and Sirois independently applied the same approach to penifulvin A,^18^ and we reported an efficient formal catalytic asymmetric synthesis.^19^ The aspergilanes, on the other hand, have withstood attempts of total synthesis. Mehta explored a Pauson–Khand approach,^20^ and more recently, Chakraborty synthesized des-hydroxy asperaculin A.^21^

At the outset, we identified ketone **1b** as the critical gateway to asperaculin A and penifulvin D. Installation of the tertiary alcohol in this structure is challenging; a 3D-projection reveals the alcohol to form part of a triad of 1,3-related *pseudo*-axial substituents. The proximal methyl group thus shields the α-carboxylate position for late-stage manipulation.^21^ We therefore devised a diastereoselective addition of a carboxyl anion equivalent to ketone **4** to create this motif. The tricyclic **4** was further deconvoluted into a simple *meta*-photocycloaddition precursor **6**. The key *meta*-photocycloaddition has an almost unrivaled history of enabling concise syntheses.^22−25^ Using high-energy light as the benign sole reagent, it introduces immense complexity that map onto biological targets from simple substrates. It is, however, also a capricious reaction, whose generality and applicability remain underexplored.

In the context of this synthesis, the utility of a chiral benzyl alcohol substrate like **6** was an outstanding question, though substrates with an oxygen tether^26^ and a benzylic acetal^27^ provided encouraging precedence. An attractive feature of the planned approach was that all stereochemical information would originate from **6**. We thus commenced with an asymmetric synthesis of this compound (Scheme 2). Direct alkylation of *o*-methyl acetophenone (**7**) with prenyl bromide followed by reduction of the resulting ketone with (+)-β-chlorodiisopinocampheylborane (DIPCl) gave **6** in multigram quantities and with an excellent 97.5:2.5 enantiomeric ratio. Unfortunately, irradiation of **6** at 254 nm gave only small amounts of photocycloadducts and a 75:25 preference for the undesired linear isomer. To address selectivity and efficiency issues, we conducted a systematic screening of alcohol protecting groups. Compared to *tert*-butyldimethysilyl, triisopropylsilyl, triethylsilyl, and tetrohydropyranyl ethers, ethoxy-methoxy (EOM) ether **8** gave a uniquely clean reaction profile when irradiated at 254 nm. A change to 300 nm light produced less byproducts and reviled a 60:40 kinetic preference in favor of the sought angular isomer **9a** at low conversions. Allylic strain between the aromatic methyl group and the EOM-ether moreover induces a sufficient conformational bias during exciplex formation to give **9a** and **9b** as single detected diastereomers. The angular-to-linear product ratio drops with conversion, in part, due to decomposition of the more sensitive angular isomer (see Supporting Information Figures S3 and S4). Optimization of solvent, reaction time, and light source clarified that irradiation in pentane with 300 nm light until 75% conversion (∼70 h) gave the highest yield, 24% of **9a** (measured by ^1^H NMR spectroscopy using an internal standard). A medium pressure mercury lamp or 254 nm light gave a much faster reaction (∼30 min) but a lower net efficiency (<15% of **9a**).

**Scheme 2:**
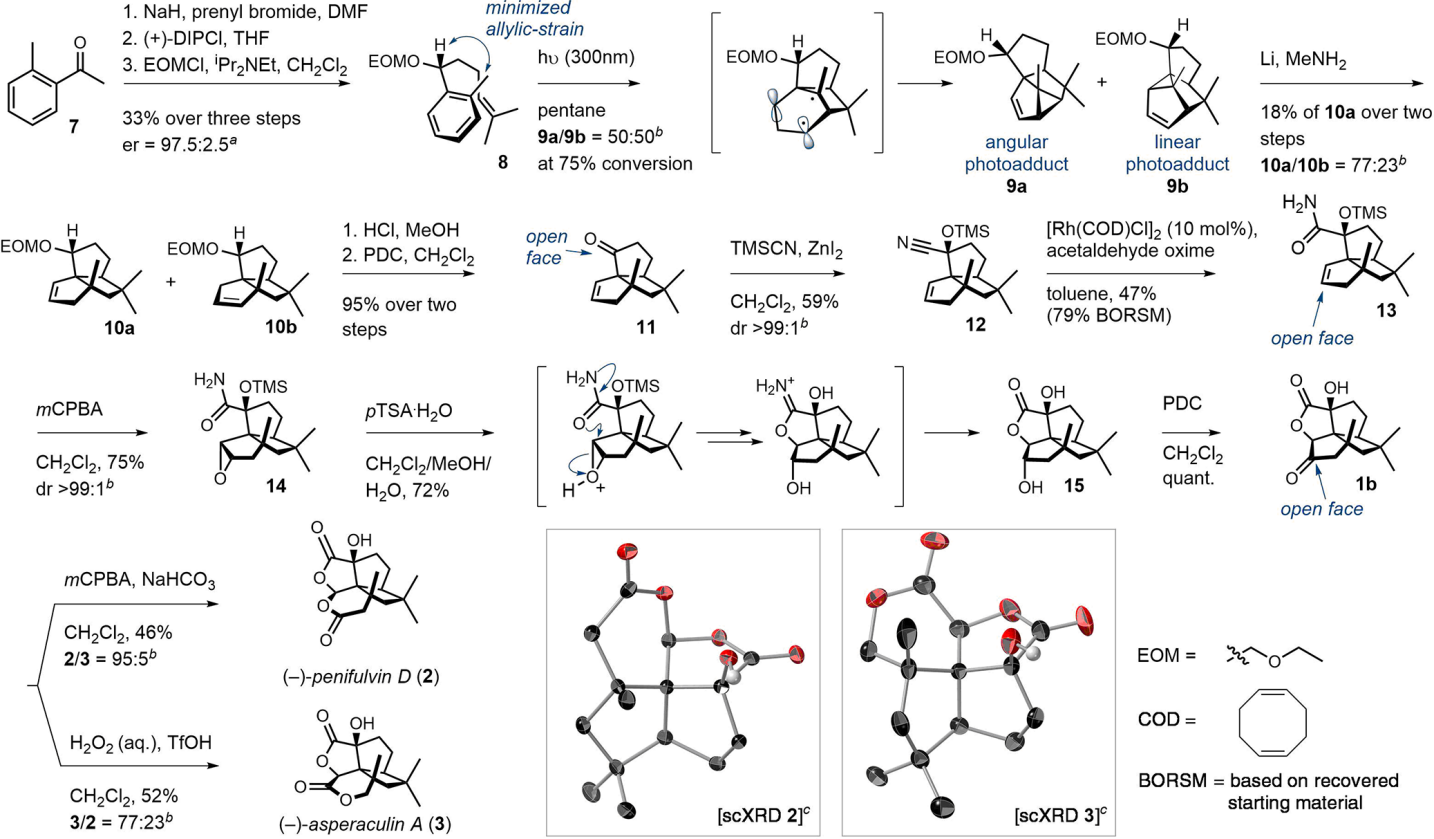
Synthesis of Asperaculin A and Penifulvin D Determined by chiral HPLC. Determined by ^1^H NMR spectroscopy of the crude reaction mixture. Thermal ellipsoids are drawn at 30% probability. Non-acidic hydrogen atoms are omitted for clarity.

A homogeneous sample of linear **9b** could be re-equilibrated to a 24:76 mixture of **9a** and **9b** by irradiation at 300 nm for 72 h. With ready access to **9a**, we turned to a radical ring opening of the vinyl cyclopropane. Because **9a** was obtained as an intractable mixture with **8**, the reduction was performed directly on the crude photomixture. Benkeser conditions with EtNH_2_ as solvent^28,16^ gave a clean reduction but produced a 74:26 mixture of isomers **10a**/**b**. We were not able to isomerize **10b** to **10a** with *p*TSA or I_2_. A switch to MeNH_2_ improved the selectivity of **10a** (77:23) and an isolated yield of 18% was obtained over two steps from **8**. Additives such as *t-*BuOH or ethylenediamine gave a lower selectivity for **10a**. We also explored ring opening of an unprotected **10a**. The free alcohol reacted very slowly with extensive side product formation under Benkeser conditions, which underlines a second purpose of the protective group in enabling efficient rupture of the cyclopropane ring. Deprotection of **10a** by acid catalysis followed by oxidation of the resulting secondary alcohol with pyridinium dichromate (PDC) then gave ketone **11** in 95% yield over two steps. Isolation of **11** is challenging, even from pentane, due to its volatility. Related to this property, we could not escape noticing its delightful marine and mineral fragrance. With scalable access to ketone **11** in hand, we turned to install the tertiary hydroxyl group.

Cyanide addition using trimethylsilyl cyanide (TMSCN)/ZnI_2_^29^ proceeded cleanly and exclusively from the molecule’s convex side. The orientation of the hydroxyl group is thus inverted compared to **8**, but prudent for the synthesis. An isolated yield of 59% for amide **12** reflects the cyanohydrin’s lability to chromatographic purification and protic workup conditions. Attempts to remove the TMS group gave an immediate release of cyanide to reform ketone **11**. Steric congestion from the 1,3-related methyl group likely contributes to this instability. To convert the nitrile into a carboxylate, we first evaluated the Parkins catalyst in EtOH/H_2_O.^30^ Stoichiometric amounts of this expensive platinum salt were needed due to poisoning of the catalyst by cyanide released from substrate decomposition. We therefore turned to nonaqueous conditions. Significantly, a rhodium-catalyzed reaction using acetaldehyde oxime as the formal water source gave amide **13**.^31^ Further optimization revealed that [Rh(COD)Cl]_2_ was as efficient as the previously used Wilkinson’s catalyst but gave a simpler workup. Moreover, it showed that a slow addition of the oxime enabled a higher conversion. Under the optimized conditions, amide **13** was obtained in 42% isolated yield, along with 37% recovered starting material.

With a completed silphinene framework in place, only an increase in the oxidation level stood between amide **13** and the target natural products. First, a Prilezhaev oxidation of the alkene with *m*CPBA gave epoxide **14** as a single diastereomer in 75% yield. The addition occurs exclusively from the olefin’s open face and thus proceeds without coordination interference from the amide group. Treatment of this epoxide with an excess of *p*TSA-hydrate then triggered a cascade that removed the silyl group and formed the first oxa-fenestrene intermediate, lactone **15**, in 72% overall yield. Oxidation of the secondary alcohol with PDC provided the gateway ketone **1b** in quantitative yield and set the stage for exploring BV oxidations.

We first turned to peracid oxidants.^32^ Pleasingly, *m*CPBA activated by either acid (TFA, *p*TSA) or base (NaHCO_3_) all gave an ∼95:5 preference for the formation of penifulvin D. The base mediated conditions were higher-yielding, and penifulvin D was obtained in 46% isolated yield. Peracetic acid gave a 90:10 selectivity. The successful formation of penifulvin D is significant, as it represents the first chemical synthesis of a ring-hydroxylated penifulvin, and it provides a simple alternative to enzyme-mediated BV oxidations in this context. We next sought conditions that would enable selective formation also of asperaculin A. Much to our delight, oxidation with H_2_O_2_ (aq.)^33^ activated by *p*TSA gave a 50:50 mixture of asperaculin A and penifulvin D. The selectivity for asperaculin A could be further increased using the noncoordinating triflic acid. By monitoring the reaction progress, we also found that the ratio increases over time due to the decomposition of the more fragile penifulvin D. Quenching the reaction at full conversion (2 min) gave a 77:23 selectivity in favor of asperaculin A and an isolated yield of 52%. Spectroscopic data for both natural products agreed with the literature values. The structures were also corroborated by single-crystal X-ray diffraction (scXRD) analysis.

To understand the intriguing reversal of selectivity in the BV oxidation, we modeled the reaction pathways^34,35^ by density functional theory (DFT) calculations using the m06-2x-d3 functional and the 6-31G** basis set (Scheme 3). As expected, only the Si-face of ketone **1b** is accessible for nucleophilic addition. Two Criegee intermediates, **CI**_**asp**_ and **CI**_**pen**_, related by rotation around the C–O bond were found for each oxidant. For the peracid reaction,^34^ the lowest paths found to each product respectively proceeded via the closed transition states **TS**_**asp**_ and **TS**_**pen**_. The calculated ΔΔ*G*^‡^_298_ was 1.9 kcal mol^–1^, which corresponds to a 96:4 ratio in favor of penifulvin D. The model thus aligns well with experimental data. A longer C···C bond to the migrating carbon in **TS**_**pen**_ than in **TS**_**asp**_ (1.82 Å vs 1.75 Å) reveals a later transition state leading to penifulvin D. Donation of electron density from the oxygen on the migrating carbon into σ*_C–C_ in **TS**_**pen**_ facilitates migration and contributes to the preference for penifulvin D. The acetal arrangement of penifulvin D is also more favored than the bis-lactone of asperaculin A reflecting in a 5.2 kcal mol^–1^ lower free energy (see Supporting Information Table S4 for a brief model study supporting the analysis). For the acid-catalyzed H_2_O_2_ oxidation, an explicit oxonium ion was needed to find computational reaction pathways with reasonable energy levels. This model replicates the experimentally observed preference for asperaculin A (ΔΔ*G*^‡^_298_ = 2.7 mol kcal^–1^) and the faster reaction rate with H_2_O_2_ compared to peracids. The critical factor behind the calculated selectivity is a stabilization by a strong electrostatic interaction between a proton on the oxonium ion and the lactone motif in **TS**_**asp**_. Such interactions are not available in **TS**_**pen**_. In absolute terms, the selectivity is overestimated by ∼1.8 kcal mol^–1^, likely connected to the difficulty of accurately accounting for the energy of solvation for protons.

**Scheme 3:**
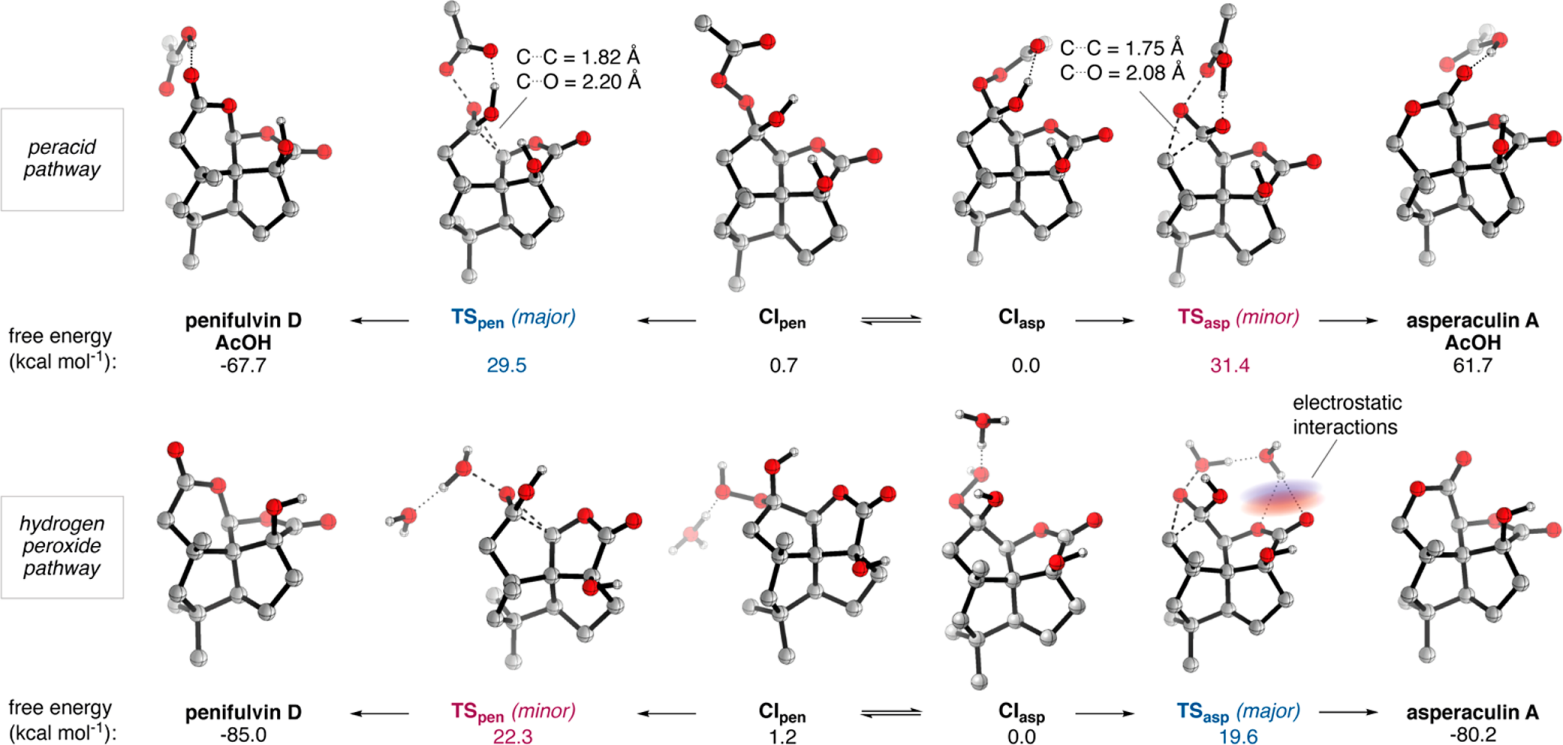
DFT Investigation of BV Regioselectivity The peracid pathway (top) is modeled with AcOOH as the oxidant. The hydrogen peroxide pathway (bottom) is modeled with H_2_O_2_/H_3_O^+^ as the oxidant. TS = transition state. CI = Criegee intermediate. Energies are calculated at 298 K.

In summary, a unifying bioinspired synthesis of (−)-asperaculin A and (−)-penifulvin D via divergent BV oxidations was completed in 13 steps from abundant *o*-methyl acetophenone. Difficult isolation of volatile intermediates and arduous purification contribute to reduced yields for certain steps. On the other hand, the approach balances such drawbacks with simple starting material, a dramatic increase in molecular complexity in the photochemical step, and a versatility to reach several targets. DFT calculations on the final BV oxidation with peracids reveal that donation of electron density from the oxygen on the migrating carbon facilitates cleavage of the C–C bond and contributes to the preference for penifulvin D. Electrostatic interactions between the oxidant leaving group and the lactone motif in turn explain the reversal of selectivity with H_2_O_2_/H_3_O^+^. It seems plausible that similar effects are at play also in enzymatic systems. Additional synthetic highlights include a diastereoselective *meta*-photocycloaddition biased by an ether substituent at the aryl α-position that broadens the scope of this remarkable reaction. The successful hydrolysis of a sensitive cyanohydrin by rhodium catalysis supports a wider utility with complex substrates. From a strategic perspective, we note that the developed pathway maps onto cyclase and oxidase phases resonant with Nature’s approach to assembling complex molecules.^36^ Efforts to further generalize divergent BV reactions and mechanistic studies on the biological activity of aspergilanes and penifulvins are ongoing in our laboratory and will be reported in due course.

## Supporting Information Available

Synthetic procedures, characterization data, and copies of ^1^H and ^13^C NMR spectra for all isolated compounds; Optimization and kinetic data for photochemistry; Crystallographic details; DFT procedures and energies; Comparison of spectroscopic data between natural and synthetic material (PDF)
DFT geometries (XYZ)

## Supplementary Material

ol1c00955_si_001.pdf

ol1c00955_si_002.xyz

## Accession Codes

CCDC 2042599–2042600 contain the supplementary crystallographic data for this paper. These data can be obtained free of charge via www.ccdc.cam.ac.uk/data_request/cif, or by emailing data_request@ccdc.cam.ac.uk, or by contacting The Cambridge Crystallographic Data Centre, 12 Union Road, Cambridge CB2 1EZ, UK; fax: +44 1223 336033.

## References

[ref1] Bach T.; Hehn J. P. Photochemical reactions as key steps in natural product synthesis. Angew. Chem., Int. Ed. 2011, 50 (5), 1000–1045. 10.1002/anie.201002845. 21246702

[ref2] Hugelshofer C. L.; Magauer T. Bioinspired total syntheses of terpenoids. Org. Biomol. Chem. 2017, 15 (1), 12–16. 10.1039/C6OB02488B. 27918058

[ref3] Ingavat N.; Mahidol C.; Ruchirawat S.; Kittakoop P.; Asperaculin A. a sesquiterpenoid from a marine-derived fungus, Aspergillus aculeatus. J. Nat. Prod. 2011, 74 (7), 1650–1652. 10.1021/np200221w. 21667999

[ref4] Shim S. H.; Gloer J. B.; Wicklow D. T. Penifulvins B-E and a silphinene analogue: sesquiterpenoids from a fungicolous isolate of Penicillium griseofulvum. J. Nat. Prod. 2006, 69 (11), 1601–1605. 10.1021/np060327z. 17125229

[ref5] Boudhar A.; Charpenay M.; Blond G.; Suffert J. Fenestranes in synthesis: unique and highly inspiring scaffolds. Angew. Chem., Int. Ed. 2013, 52 (49), 12786–12798. 10.1002/anie.201304555. 24281879

[ref6] Das D.; Chakraborty T. K. An overview of the recent synthetic studies toward penifulvins and other fenestranes. Tetrahedron Lett. 2016, 57 (33), 3665–3677. 10.1016/j.tetlet.2016.07.011.

[ref7] Zeng H.; Yin G.; Wei Q.; Li D.; Wang Y.; Hu Y.; Hu C.; Zou Y. Unprecedented [5.5.5.6]Dioxafenestrane Ring Construction in Fungal Insecticidal Sesquiterpene Biosynthesis. Angew. Chem., Int. Ed. 2019, 58 (20), 6569–6573. 10.1002/anie.201813722. 30908782

[ref8] Wei Q.; Zeng H.-C.; Zou Y. Divergent Biosynthesis of Fungal Dioxafenestrane Sesquiterpenes by the Cooperation of Distinctive Baeyer–Villiger Monooxygenases and α-Ketoglutarate-Dependent Dioxygenases. ACS Catal. 2021, 11 (2), 948–957. 10.1021/acscatal.0c05319.

[ref9] Geibel I.; Dierks A.; Muller T.; Christoffers J. Formation of delta-Lactones with anti-Baeyer-Villiger Regiochemistry: Investigations into the Mechanism of the Cerium-Catalyzed Aerobic Coupling of beta-Oxoesters with Enol Acetates. Chem. - Eur. J. 2017, 23 (30), 7245–7254. 10.1002/chem.201605468. 28230284

[ref10] Romney D. K.; Colvin S. M.; Miller S. J. Catalyst control over regio- and enantioselectivity in Baeyer-Villiger oxidations of functionalized ketones. J. Am. Chem. Soc. 2014, 136 (40), 14019–14022. 10.1021/ja508757g. 25250713 PMC4195385

[ref11] Itoh Y.; Yamanaka M.; Mikami K. Complete reversal in regioselectivity in the Baeyer-Villiger reaction of an alpha-CF(3)-ketone and theoretical rationale for axial orientation of sterically demanding CF(3) group at the transition state. Org. Lett. 2003, 5 (25), 4803–4806. 10.1021/ol0358298. 14653678

[ref12] Harmata M.; Rashatasakhon P. Observations on the regioselectivity of some Baeyer–Villiger reactions. Tetrahedron Lett. 2002, 43 (20), 3641–3644. 10.1016/S0040-4039(02)00616-0.

[ref13] Cossy J.; Gille B.; Bellosta V. Synthesis of spirocyclic bislactones substituent effects on the regioselectivity of the Baeyer-Villiger of 1,3-diketones. Tetrahedron Lett. 1998, 39 (25), 4459–4462. 10.1016/S0040-4039(98)00816-8.

[ref14] Corey E. J.; Kang M. C.; Desai M. C.; Ghosh A. K.; Houpis I. N. Total Synthesis of (±)-Ginkgolide B. J. Am. Chem. Soc. 1988, 110 (2), 649–651. 10.1021/ja00210a083. 31527923 PMC6746322

[ref15] Shim S. H.; Swenson D. C.; Gloer J. B.; Dowd P. F.; Wicklow D. T.; Penifulvin A. a sesquiterpenoid-derived metabolite containing a novel dioxa[5,5,5,6]fenestrane ring system from a fungicolous isolate of Penicillium griseofulvum. Org. Lett. 2006, 8 (6), 1225–1228. 10.1021/ol060107c. 16524309

[ref16] Gaich T.; Mulzer J. Total synthesis of (−)-Penifulvin A, an insecticide with a dioxafenestrane skeleton. J. Am. Chem. Soc. 2009, 131 (2), 452–453. 10.1021/ja8083048. 19140787

[ref17] Gaich T.; Mulzer J. From silphinenes to penifulvins: a biomimetic approach to penifulvins B and C. Org. Lett. 2010, 12 (2), 272–275. 10.1021/ol902594b. 20000335

[ref18] Sirois L. E. Investigations of [5 + 2] and other cycloadditions: new catalysts, regioselectivity, novel serial processes, and applications i synthesis. Ph.D., Stanford University, 2011.

[ref19] Melcher M. C.; Ivsic T.; Olagnon C.; Tenten C.; Lutzen A.; Strand D. Control of Enantioselectivity in Rhodium(I) Catalysis by Planar Chiral Dibenzo[a,e]cyclooctatetraenes. Chem. - Eur. J. 2018, 24 (10), 2344–2348. 10.1002/chem.201704816. 29131428

[ref20] Mehta G.; Khan T. B. Model studies toward a synthesis of asperaculin A: exploration of iterative intramolecular Pauson-Khand reaction based strategies to access the dioxa[5.5.5.6]fenestrane framework. Tetrahedron Lett. 2012, 53 (34), 4558–4561. 10.1016/j.tetlet.2012.06.059.

[ref21] Das D.; Chakraborty T. K. Radical Approach to the Chiral Quaternary Center in Asperaculin A: Synthesis of 9-Deoxyasperaculin A. Org. Lett. 2017, 19 (3), 682–685. 10.1021/acs.orglett.6b03854. 28105814

[ref22] Cornelisse J. The Meta Photocycloaddition of Arenes to Alkenes. Chem. Rev. 1993, 93 (2), 615–669. 10.1021/cr00018a002.

[ref23] Karkas M. D.; Porco J. A. Jr.; Stephenson C. R. Photochemical Approaches to Complex Chemotypes: Applications in Natural Product Synthesis. Chem. Rev. 2016, 116 (17), 9683–9747. 10.1021/acs.chemrev.5b00760. 27120289 PMC5025835

[ref24] Wender P. A.; Howbert J. J. Synthetic studies on arene-olefin cycloadditions -III- total synthesis of (±)-hirsutene. Tetrahedron Lett. 1982, 23 (39), 3983–3986. 10.1016/S0040-4039(00)88675-X.

[ref25] Wender P. A.; Ternansky R. J. Synthetic studies on arene-olepin cycloadditions-VII:1 a three-step total synthesis of (±)-silphinene. Tetrahedron Lett. 1985, 26 (22), 2625–2628. 10.1016/S0040-4039(00)98120-6.

[ref26] Wegmann M.; Bach T. Influence of the -CH2X substituent on the regioselectivity of intramolecular meta-photocycloaddition reactions. J. Org. Chem. 2015, 80 (3), 2017–2023. 10.1021/jo5028613. 25587911

[ref27] Wender a. P. A.; deLong M. A. Synthetic studies on arene-olefin cycloadditions. XII. Total synthesis of (±)-subergorgic acid. Tetrahedron Lett. 1990, 31 (38), 5429–5432. 10.1016/S0040-4039(00)97864-X.

[ref28] Dhatrak N. R. Birch and Benkeser Reductions Application of Electride Salts in Organic Chemistry. Resonance 2019, 24 (7), 735–740. 10.1007/s12045-019-0835-z.

[ref29] Evans D. A.; Carroll G. L.; Truesdale L. K. Synthetic Applications of Trimethylsilyl Cyanide - Efficient Synthesis of Beta-Aminomethyl Alcohols. J. Org. Chem. 1974, 39 (7), 914–917. 10.1021/jo00921a012.

[ref30] Cadierno V. Synthetic Applications of the Parkins Nitrile Hydration Catalyst [PtH{(PMe2O)2H}(PMe2OH)]: A Review. Appl. Sci. 2015, 5 (3), 380–401. 10.3390/app5030380.

[ref31] Lee J.; Kim M.; Chang S.; Lee H. Y. Anhydrous hydration of nitriles to amides using aldoximes as the water source. Org. Lett. 2009, 11 (24), 5598–5601. 10.1021/ol902309z. 19911824

[ref32] ten Brink G. J.; Arends I. W.; Sheldon R. A. The Baeyer-Villiger reaction: new developments toward greener procedures. Chem. Rev. 2004, 104 (9), 4105–4124. 10.1021/cr030011l. 15352787

[ref33] Uyanik M.; Ishihara K. Baeyer-Villiger Oxidation Using Hydrogen Peroxide. ACS Catal. 2013, 3 (4), 513–520. 10.1021/cs300821u.

[ref34] Bach R. D. The role of acid catalysis in the Baeyer-Villiger reaction. A theoretical study. J. Org. Chem. 2012, 77 (16), 6801–6815. 10.1021/jo300727w. 22849715

[ref35] Alvarez-Idaboy J. R.; Reyes L.; Mora-Diez N. The mechanism of the Baeyer-Villiger rearrangement: quantum chemistry and TST study supported by experimental kinetic data. Org. Biomol. Chem. 2007, 5 (22), 3682–3689. 10.1039/b712608e. 17971998

[ref36] Kanda Y.; Ishihara Y.; Wilde N. C.; Baran P. S. Two-Phase Total Synthesis of Taxanes: Tactics and Strategies. J. Org. Chem. 2020, 85 (16), 10293–10320. 10.1021/acs.joc.0c01287. 32663002

