# Supplementary material for: A Unifying Bioinspired Synthesis of (−)-Asperaculin A and (−)-Penifulvin D
Source: Org Lett. 2021 Apr 8;23(9):3536–40. doi: 10.1021/acs.orglett.1c00955 (PMC8155558; doi:10.1021/acs.orglett.1c00955)

Supporting Information

A Unifying Bio-inspired Synthesis of (–)-Asperaculin A and (–)-Penifulvin D

Ian R. George, Miguel López-Tena, Anders P. Sundin, and Daniel Strand*

Centre for Analysis and Synthesis, Department of Chemistry, Lund university Box 124, 221 00 Lund, Sweden.

Index

I.	*General procedures*	*S2*
II.	*Experimental procedures and analytical data for **6**, **8**, **9a**, **9b**, **10a**, **S3**, **11-15**, **1b**, **2**, and **3***	*S5*
III.	*Comparison of synthetic and natural **2** and **3***	*S23*
IV.	*Single crystal X-ray diffraction (scXRD) analysis of **2** and **3***	*S25*
V.	*Optimization and kinetics of meta-photocycloaddition of **8***	*S27*
VI.	*Photoisomerization of **9b** to **9a***	*S29*
VII.	*Density functional theory calculations*	*S30*
VII.	*Comparison of heat of formation between model acetal **S4** and bis-lactone **S5***	*S35*
VIII.	Copies of *^{1}H and ^{13}C NMR spectra for compounds **6**, **8**, **9a**, **10a**, **10b**, **S3**, **11-15**, **1b**, **2**, and **3***	*S36*

SAFETY: No unusual safety issues or concerns were encountered in this work. Ethyl-oxy-methyl chloride is a suspected carcinogen and we encourage a detailed risk assessment and additional training before handling. Baeyer-Villiger oxidations with H_2O_2 and TfOH were conducted on a small scale. We recommend that application of these conditions on a larger scale is preceded by a detailed risk assessment with respect to potentially explosive intermediates.

I. General Procedures.

Equipment: All reactions were conducted in dry glassware. Drying was accomplished by evacuation of the vessel followed by heating with a hot air gun for >5 minutes. The vessels were then backfilled with dry nitrogen or argon gas, and allowed to cool to ambient

temperature. Unless otherwise stated, all reactions were conducted under an inert atmosphere. Dry argon was supplied from a balloon and dry nitrogen from a manifold equipped with a bubbler. Elevated temperatures were achieved using either silicon oil baths or aluminum heating blocks on a thermostat-controlled heating plate. Unless otherwise stated, large scale reactions were conducted in round bottom flasks equipped with a magnetic stir bar. Reactions of volumes below 20 mL were conducted in Biotage microwave vessels equipped with a magnetic stir bar. Analytical HPLC was performed using a Shimadzu Prominance-i LC-2030C 3D system.

Reagents and Solvents: All reagents and solvents were bought from commercial suppliers and used as received unless otherwise stated. Tetrahydrofuran was distilled from sodium benzophenone ketyl radical under an atmosphere of nitrogen. Acetonitrile was distilled from calcium hydride under an atmosphere of nitrogen. Dichloromethane, hexane and toluene were obtained from an MBraun MB-SPS 800 solvent purification system.

Photochemistry: Photochemical reactions were performed in a Rayonet RPR-100 photoreactor equipped with sixteen 254 nm or 300 nm lamps. Preparative scale reactions were performed in a Rayonet RQV-218 660 mL GE quartz 214 quartz vessel stoppered with a rubber septum. Small scale (<150 mg) reactions were performed in custom made quartz tubes (Herasil) sealed with Biotage microwave vessel caps. Reactions with a medium pressure mercury lamp were performed using a Heraeus Noble light TNN 15/32-1721, HNG Germany lamp in a custom built Herasil quartz reactor.

NMR Spectroscopy: ^{1}H and ^{13}C (^{1}H decoupled) data were collected on a Bruker Avance II 400 MHz (^{1}H 400 MHz; ^{13}C 101 MHz) and/or Bruker Avance III HD 500 MHz (^{1}H 500 MHz, ^{13}C 126 MHz) spectrometers, both equipped with a 5 mm BBOF Z-gradient probe. Chemical shifts are reported in parts per million (ppm) and coupling constants (*J*) are given in hertz. All

data was recorded in $CDCl_3$ and referenced to residual $CHCl_3$ (δ 7.26 ^{1}H; 77.16 ^{13}C). Multiplicities are represented by singlet (s), doublet (d), triplet (t), quartet (q), multiplet (m) and broad peaks noted (br.). $CDCl_3$ was passed through activated basic aluminum oxide (Brockmann I) before use to remove trace deuterium chloride.

IR Spectroscopy: IR spectra were recorded on a Bruker Alpha II spectrometer as thin films using the ATR attachment. Frequencies are reported in wavenumbers (cm^{-1}) and peaks denoted as strong (s) and broad (br.) where applicable.

UV-Vis Spectroscopy: UV-Vis spectra were recorded with an Agilent Cary 60 UV/Vis spectrometer using quartz cuvettes.

Specific Rotation: Optical rotations $[\alpha]_D$ were recorded at ambient temperature using Perkin Elmer model 341 polarimeter, where D represents the sodium D line (589 nm). Concentrations (*c*) are reported in g/100mL.

Mass Spectrometry: HRMS data was obtained using an ESI-QTOF mass spectrometer (Waters Xevo-G2) in positive mode between m/z 50-1200, employing lockmass correction according to the manufacturer's instructions.

Chromatography: Thin layer chromatography (TLC) was performed using Merck 60 F_{254} silica gel bound to aluminum plates. TLC plates were cut to 30 mm x 50 mm. For consistent calculation of retention factors (R_f) the solvent was eluted to ~27 mm above the baseline. The plates were visualized using UV light (254 nm), and cerium ammonium molybdate stain (CAM) (ceric ammonium sulfate dihydrate (0.50 g, 790 μmol), ammonium molybdate tetrahydrate (12 g, 970 μmol), conc. H_2SO_4 (15 mL), H_2O (235 mL)). Purification by flash column chromatography was performed using Merck 60 Å (40-63 μm particle size) silica. 'Deactivated silica' refers to silica which has been slurried with triethylamine added to the

eluent. After packing, ~5 column volumes of neutral eluent were passed through before compound is loaded.

*II. Experimental procedures and analytical data for **6, 8, 9a, 9b, 10a, S3, 11-15, 1b, 2, and 3***

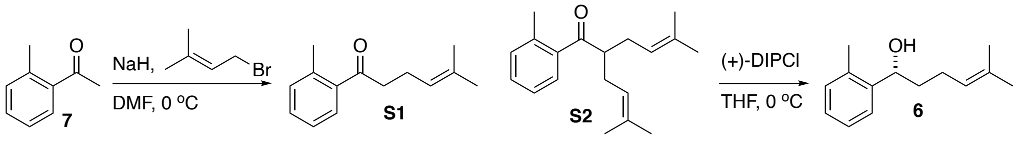

(*R*)-5-Methyl-1-(*o*-tolyl)hex-4-en-1-ol (6).[1] To a stirred suspension of sodium hydride 60% mineral oil dispersion (2.2 g, 59 mmol) in DMF (60 mL) was added 2′-methylacetophenone (6.7 mL, 50 mmol) dropwise via syringe at 0 ºC. After 1 h, the deep orange solution was transferred via canula to a stirred solution of 3,3-dimethylallylbromide (9.1 mL, 75 mmol) in DMF (25 mL), over 20 min, at 0 ºC, then rinsed with a further 40 mL of DMF. Immediately after completion of the addition, the reaction was quenched with water. The aqueous phase was extracted with pentane (x 3) and the combined organic layers were washed with brine, dried (Na_2SO_4, then filtered), and the filtrate concentrated under reduced pressure to give a 34 : 45 : 21 mixture of acetophenone **7** : **S1**[2] : **S2** as a yellow oil (12.0 g). A solution of this mixture (9.50 g) in THF (10 mL) was added via syringe, drop-wise over 30 min, to a stirred solution of (+)-DIP-Cl (19.0 g, 60 mmol) in THF (200 mL) at 0 ºC. The solution was warmed to room temperature over 22 h, then a solution of diethanolamine (9.30 mL, 88 mmol) in ethyl acetate (60 mL) was added. After 5 minutes, the mixture was filtered through a plug of cotton wool,

[1] The absolute configuration of the major enantiomer was assigned following the model of Brown. For details, see: Brown, H; Ramachandran, V. Asymmetric Reduction with Chiral Organoboranes Based on α-Pinene, *Acc. Chem. Res.* **1992**, *25*, 16-24.

[2] Known compound. For an alternative preparation, see: Tu, J; Liu, J; Tang, W; Su, M; Liu, F. Radical Aza-Cyclization of α-Imino-oxy Acids for Synthesis of Alkene-Containing N-Heterocycles via Dual Cobaloxime and Photoredox Catalysis. *Org. Lett.* **2020**, 22, 1222-1226.

and the filtrate concentrated under reduced pressure. The resulting oil was diluted water and pentane. The aqueous phase was extracted with pentane (x 3) and the combined organic layers were washed with brine, dried (Na_2SO_4, then filtered) and the filtrate concentrated under reduced pressure to give a yellow oil, which was purified by flash chromatography (2 – 6 % ethyl acetate/heptane) to give alcohol **6**.

Yield: 2.74 g (37%). Isolated as a pale yellow oil containing 60 wt% ethyl acetate (4.54 g total mass). To avoid mass-loss during further concentration, the mixture was typically used directly in the subsequent step. Analytical data is given for a >95% homogeneous sample.

R*f*: 0.19 in 10% diethyl ether in pentane. UV active and stains blue with CAM.

$[\alpha]_D$: + 35 (*c* = 1.00, CH_2Cl_2).

FT IR (Neat oil): 3359 (br), 3023, 2964, 2915, 2857, 1741, 1725, 1487, 1449, 1375, 1330, 1240, 1181, 1158, 1104, 1061, 1049, 1005, 943, 9118, 867, 833, 805, 755 (s), 726, 663, 629, 614, 562, 510, 455, 422 cm^{-1}.

^{1}H NMR ($CDCl_3$, 400 MHz): δ 7.47 (dd, *J* = 7.6, 1.4 Hz, 1H), 7.23 (ddd, *J* = 7.6, 7.4, 1.4 Hz, 1H), 7.17 (ddd, *J* = 7.6, 7.4, 1.2 Hz, 1H), 7.13 (ddd, *J* = 7.4, 1.4, 0.6 Hz, 1H), 5.18 (tqq, *J* = 7.2, 1.4, 1.2 Hz, 1H), 4.93 (dt, *J* = 8.1, 4.7 Hz, 1H), 2.33 (s, 3H), 2.08 - 2.21 (m, 2H), 1.65 - 1.90 (m, 3H), 1.71 (d, *J* = 1.2 Hz, 3H), 1.62 (br. s, 3H) ppm.

^{13}C NMR ($CDCl_3$, 101 MHz): δ 143.1, 134.5, 132.6, 130.5, 127.2, 126.4, 125.2, 123.9, 70.5, 38.1, 25.8, 24.8, 19.1, 17.8 ppm.

HRMS (ESI-QTOF) (*m/z*): $[M + Na]^+$ Calcd for $C_{14}H_{20}NaO$ 227.1412; Found 227.1406.

HPLC (AD-H, *n*-hexane/2-propanol = 97/3, flow rate = 1.0 mL/min, λ = 268 nm): t_R = 7.5 min (major), 8.1 min (minor).

Enantiomeric ratio: 97.5 : 2.5

HPLC trace for (*R*)-**6**:

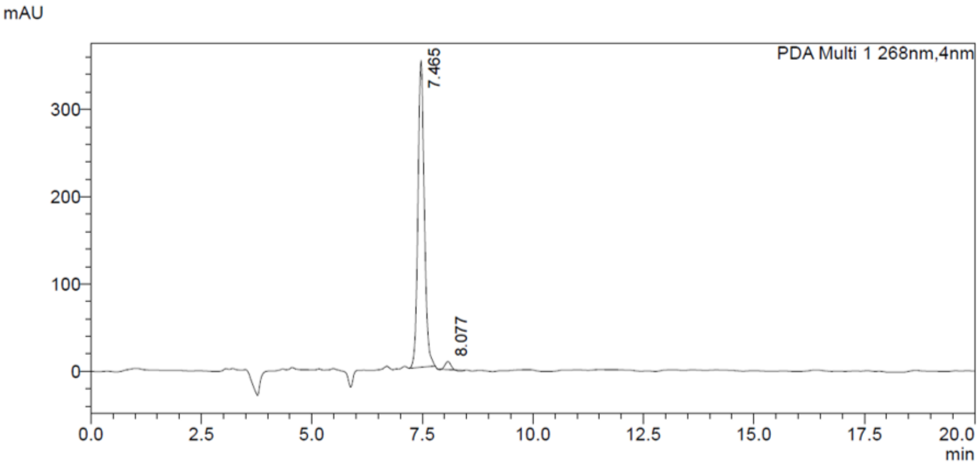

Peak	Retention time (min)	Area	Height
1	7.465	3152389	349856
2	8.077	68245	9116

HPLC trace for *rac*-**6**:

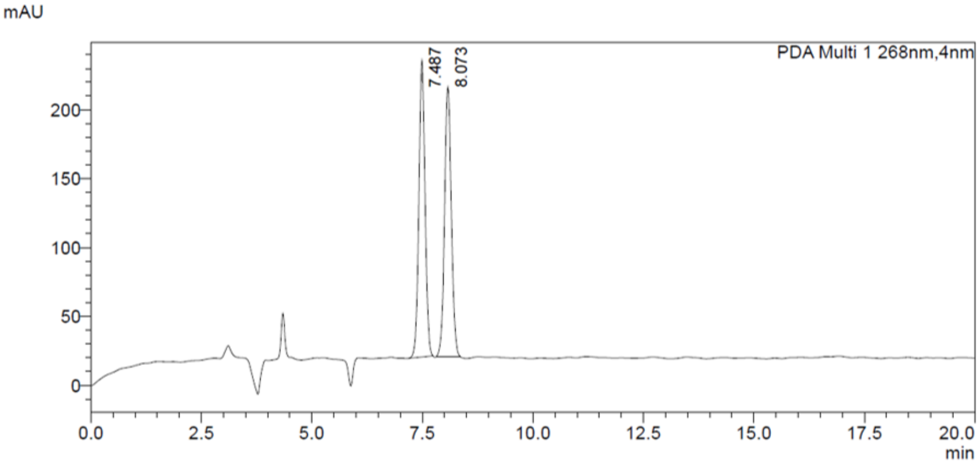

Peak	Retention time (min)	Area	Height
1	7.487	2093136	214997
2	8.073	2085623	195750

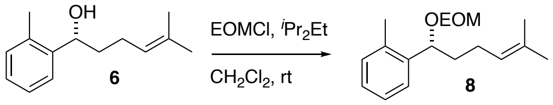

(*R*)-1-(1-(Ethoxymethoxy)-5-methylhex-4-en-1-yl)-2-methylbenzene (8). To a solution of alcohol **6** (4.50 g, 22.1 mmol) in dichloromethane (220 mL) at 0 °C was added Hünig's base

(24.0 mL, 140 mmol) in one portion via syringe. Ethyl-oxy-methyl chloride (4.0 mL, 43.0 mmol) was then added dropwise via syringe over 5 minutes and the resulting mixture was stirred at room temperature for 24 h. After this time, the reaction mixture was quenched by addition of ammonium chloride (sat. aq.) after which the aqueous phase was extracted with dichloromethane (x 3). The combined organic layers were washed with ammonium chloride (sat. aq.), brine, dried (Na_2SO_4, then filtered) and the filtrate concentrated under reduced pressure to give an orange oil. The oil was purified by flash chromatography (3% ethyl acetate/pentane) to give the ether **8**.

Yield: 5.07 g (88%). Isolated as a pale yellow oil, >95% pure by NMR spectroscopy and a single spot by TLC.

R_f: 0.22 in 3% Ethyl acetate in pentane. UV active and stains blue with CAM.

$[\alpha]_D$: + 133 (c = 1.00, CH_2Cl_2).

UV-Vis (Pentane): λ_{Max} 264 nm (logε = 3.14), λ_{Min} 269 nm (logε = 2.96), λ_{Max} 272 nm (logε = 3.04).

FT IR (Neat oil): 2972, 2928, 2879, 1488, 1447, 1377, 1180, 1147, 1097, 1074, 1027 (s), 937, 897, 845, 756, 727, 451 cm^{-1}.

^{1}H NMR ($CDCl_3$, 400 MHz): δ 7.37 (dd, J = 7.6, 1.3 Hz, 1H), 7.19 (ddd, J = 7.6, 7.4, 1.3 Hz, 1H), 7.14 (td, J = 7.4, 1.7 Hz, 1H), 7.10 (dd, J = 7.4, 1.7 Hz, 1H), 5.14 (tqq, J = 7.0, 1.4, 1.2 Hz, 1H), 4.85 (dd, J = 8.5, 4.7 Hz, 1H), 4.61 (d, J = 6.8 Hz, 1H), 4.49 (d, J = 6.8 Hz, 1H), 3.73 (dq, J = 9.5, 7.1 Hz, 1H), 3.46 (dq, J = 9.5, 7.1 Hz, 1H), 2.33 (s, 3H), 2.00 - 2.21 (m, 2H), 1.82 (dtd, J = 14.0, 8.5, 5.6 Hz, 1H), 1.68 (d, J = 1.2 Hz, 3H), 1.65 (dddd, J = 14.0, 9.4, 6.8, 4.7 Hz, 3H), 1.61 (br.s, 3H), 1.19 (t, J = 7.1 Hz, 3H) ppm.

^{13}C NMR ($CDCl_3$, 101 MHz): δ 140.7, 135.5, 132.2, 130.3, 127.1, 126.3, 126.2, 124.1, 93.0, 74.1, 63.5, 37.4, 25.8, 24.7, 19.2, 17.8, 15.2 ppm.

HRMS (ESI-QTOF) (*m/z*): $[M + Na]^+$ Calcd for $C_{17}H_{26}NaO_2$ 285.1830; Found 285.1829.

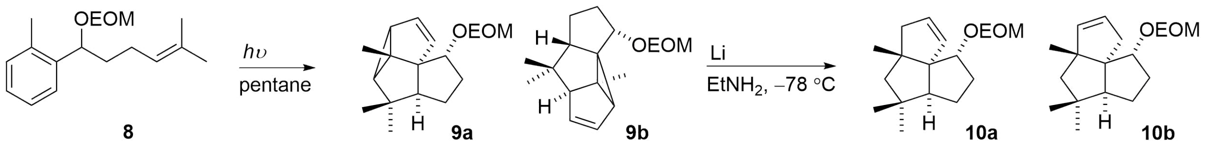

(4*S*,7*S*,8*S*,9*R*)-9-(Ethoxymethoxy)-6,6,4-trimethyl-9,10,11,7,6,5,4,3-octahydrocyclopenta [c]pentalene (10a). Arene-olefin **8** (5.00 g, 19 mmol) was dissolved in pentane (660 mL) in a quartz glass vessel. The solution was degassed by evacuation until gas evolution was seen and then back-filled with nitrogen for a total of six cycles. The reaction mixture was then irradiated at 300 nm in a Rayonet RPR-100 photoreactor for five days. The progress of the reaction was monitored daily by ^{1}H NMR spectroscopy (irradiation was stopped, an aliquot was drawn, and the mixture was degassed as described before irradiation was reinitiated). At about 75% conversion irradiation was ceased and the mixture was concentrated under reduced pressure to give a yellow oil (7.29 g) which was used directly in the next reaction without further purification. The amount of product and starting material in this sample was measured by ^{1}H NMR spectroscopy using 1-methoxynaphthalene as an internal standard. In a typical experiment the mixture to contained angular cycloadduct **9a** 1.20 g (24%), linear cycloadduct **9b** 1.20 g (24%) along with starting material **8** 1.14 g (23%).

Angular cycloadduct 9a[3]

^{1}H NMR ($CDCl_3$, 400 MHz): δ 5.66 (dd, *J* = 5.4, 2.2 Hz, 1 H), 5.53 (dt, *J* = 5.4, 0.8 Hz, 1 H), 4.67 (d, *J* = 6.8 Hz, 1 H), 4.62 (d, *J* = 6.8 Hz, 1 H), 4.23 - 4.30 (m, 1 H), 3.63 (dt, *J* = 9.7, 7.1

[3] Despite repeated attempts of chromatographic purification, **9a** was obtained as a mixture with arene-olefin **8.** Full characterization of a homogeneous sample was not achieved.

Hz, 1 H), 3.54 (dt, *J* = 9.7, 7.1 Hz, 1 H), 2.20 - 2.27 (m, 1 H), 2.00 - 2.08 (m, 1 H), 1.48 - 1.67 (m, 3 H), 1.45 (dd, *J* = 7.4, 2.2 Hz, 1 H), 1.34 (s, 3 H), 1.29 (d, *J* = 7.4 Hz, 1 H), 1.20 (t, *J* = 7.1 Hz, 3 H), 1.12 (s, 3 H), 1.01 (s, 3 H) ppm

^{13}C NMR ($CDCl_3$, 101 MHz): δ 134.5, 127.9, 94.0, 77.0*, 72.4, 69.0, 63.1, 49.9, 48.5, 41.3, 38.4, 35.0, 29.3, 28.9, 26.3, 18.6, 15.1 ppm.

*Peak identified through DEPT135 and ^{13}C-HMBC spectroscopy.

Linear cycloadduct 9b

Yield : 166 mg (19%). Isolated as a pale yellow oil, >95% pure by NMR spectroscopy and a single spot by TLC.

R*f*: 0.70 in 5% EtOAc/pentane. UV inactive but stains blue with CAM.

$[\alpha]_D$: + 31 (*c* = 1.00, CH_2Cl_2).

FT IR (Neat oil): 3055, 2952, 2091, 2874, 1469, 1449, 1390, 1379, 1361, 1186, 1147, 1098, 1040 (s), 1011, 953, 918, 889, 847, 804, 785, 748 cm^{-1}.

UV/Vis (Pentane): λ_{Max} 200 nm (logε = 3.48), λ_{Max} 234 nm (logε = 3.51).

^{1}H NMR ($CDCl_3$, 400 MHz): δ 5.78 (dd, *J* = 5.5, 2.2 Hz, 1 H), 5.51 (ddd, *J* = 5.5, 2.4, 0.8 Hz, 1 H), 4.72 (d, *J* = 7.1 Hz, 1 H), 4.65 (d, *J* = 7.1 Hz, 1 H), 3.79 (dd, *J* = 3.7, 2.0 Hz, 1 H), 3.65 (dt, *J* = 9.5, 7.2 Hz, 1 H), 3.55 (dt, *J* = 9.5, 7.2 Hz, 1 H), 2.42 (d, *J* = 2.4 Hz, 1 H), 2.07 (dd, *J* = 9.3, 2.3 Hz, 1 H), 1.93 - 2.01 (m, 1 H), 1.76 - 1.89 (m, 2 H), 1.66 - 1.74 (m, 1 H), 1.50 (dt, *J* = 5.6, 2.8 Hz, 1 H), 1.33 (s, 3 H), 1.21 (t, *J* = 7.2 Hz, 3 H), 0.93 (s, 3 H), 0.79 (s, 3 H) ppm.

^{13}C NMR ($CDCl_3$, 101 MHz): δ 131.9, 130.1, 93.4, 78.3, 68.2, 63.0, 55.8, 53.2, 49.5, 48.4, 39.3, 34.9, 24.0, 22.9, 22.6, 16.6, 15.3 ppm.

HRMS (ESI-QTOF) (*m/z*): $[M + Na]^+$ Calcd for $C_{17}H_{26}NaO_2$ 285.1830; Found 285.1826.

Methylamine (~ 40 mL) was condensed into a three-necked flask held at −78 °C. A solution of the crude product from the *meta*-photocycloaddition (7.29 g) in THF (10 mL) was then added in one portion via syringe under a nitrogen atmosphere. Small pieces of lithium metal (total 1.3 g, 190 mmol) were cut directly into the stirred solution against a positive flow of nitrogen. The resulting mixture first turned red, then purple, and finally a deep persistent blue color emerged. The mixture was then stirred at –78 °C for 3 h after which ammonium chloride (sat. aq.) and ether were added carefully until the blue coloration faded and the mixture was warmed to 0 °C under a positive flow of nitrogen. Ammonium chloride (sat. aq) was then added in small portions until the remaining pieces of lithium metal were completely consumed. The aqueous phase was extracted with ether (x 3) and the combined organic layers were washed with ammonium chloride (sat. aq), brine, dried (Na_2SO_4, then filtered), and the filtrate concentrated under reduced pressure. The resulting crude oil (5.68 g) contained a mixture of angular product **10a** along with its double bond isomer **10b**[4] in a 77 : 23 ratio as determined by ^{1}H NMR spectroscopy. Purification by flash chromatography (0 – 5% ether/pentane) gave olefin **10a** as a single isomer.

Yield: 919 mg (18% from arene-olefin **8**). Isolated as a colorless oil, >95% pure by ^{1}H NMR, and a single spot by TLC.

R*f*: 0.33 in 5% diethyl ether in pentane. Faintly UV active at very high concentrations and stains blue with CAM.

[α]D: − 13 (*c* = 1.00, CH_2Cl_2).

---

[4] **NMR spectroscopy data for 10b: ^{1}H NMR ($CDCl_3$, 400 MHz):** 5.50 (ddd, *J* 5.7, 2.2, 2.0 Hz, 1 H), 5.45 (ddd, *J* 5.7, 2.2, 2.0 Hz, 1 H), 4.75 (d, *J* 6.9 Hz, 1 H), 4.66 (d, *J* 6.9 Hz, 1 H), 3.86 (dd, *J* 8.1, 5.3 Hz, 1 H), 3.62 (dq, *J* = 9.4, 7.0, 1H), 3.57 (dq, *J* = 9.4, 7.0, 1H), 3.04 (dt, *J* 17.3, 2.2 Hz, 1 H), 2.16 (dt, *J* 17.2, 2.0 Hz, 1 H), 1.83 - 2.01 (m, 1H), 1.92 (dd, *J* 10.5, 6.6 Hz, 1 H), 1.66 - 1.73 (m, 1 H), 1.63 (d, *J* 13.1 Hz, 1 H), 1.49 - 1.58 (m, 1 H), 1.45 (d, *J* 13.1 Hz, 1 H), 1.31 - 1.40 (m, 1 H), 1.20 (t, *J* = 7.0 Hz, 3 H), 1.01 (3 H, s), 0.99 (3 H, s), 0.88 (3 H, s) ppm; **^{13}C NMR ($CDCl_3$, 101 MHz):** δ 141.9, 126.3, 94.5, 81.4, 65.2, 64.5, 63.4, 57.9, 54.0, 41.8, 39.4, 33.2, 31.9, 27.6, 24.2, 23.8, 15.2 ppm.

FT IR (Neat oil): 3048, 2949, 2931, 2869, 1449, 1385, 1364, 1290, 1177, 1148, 1110, 1098, 1039 (s), 1014, 920, 846, 734, 711, 642 cm^{-1}.

^{1}H NMR ($CDCl_3$, 400 MHz): δ 5.72 (ddd, *J* = 5.7, 2.3, 2.2, 1H), 5.51 (ddd, *J* = 5.7, 2.3, 2.2 Hz, 1H), 4.70 (d, *J* = 7.0 Hz, 1 H), 4.62 (d, *J* = 7.0 Hz, 1 H), 4.04 (dd, *J* = 8.2, 5.7 Hz, 1H), 3.55 (dq, *J* = 9.4, 7.1, 1H), 3.53 (dq, *J* = 9.4, 7.1 Hz, 1H), 2.53 (dt, *J* = 17.2, 2.3 Hz, 1H), 2.26 (dt, *J* = 17.2, 2.2 Hz, 1H), 2.01 - 2.12 (m, 1H), 1.93 (t, *J* = 8.7 Hz, 1H), 1.69 (d, *J* = 13.1 Hz, 1H), 1.59 (d, *J* = 13.1 Hz, 1H), 1.54 - 1.72 (m, 1H), 1.23 - 1.34 (m, 2H), 1.19 (t, *J* = 7.1 Hz, 3 H), 1.12 (s, 3H), 1.00 (s, 3H), 0.90 (s, 3H) ppm.

^{13}C NMR ($CDCl_3$, 101 MHz): δ 137.4, 126.5, 94.2, 81.0, 73.0, 63.2, 61.2, 57.6, 51.3, 49.6, 40.2, 34.0, 30.6, 27.4, 27.3, 24.8, 15.2 ppm.

HRMS (ESI-QTOF) (*m/z*): A mass ion corresponding to the expected *m/z* could not be found.

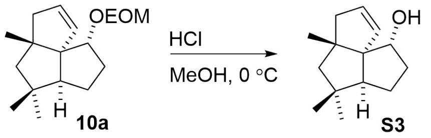

(4*S*,7*S*,8*S*,9*R*)-6,6,4-Trimethyl-9,10,11,7,6,5,4,3-octahydrocyclopenta[c]pentalen-9-ol (S3). Methanolic hydrogen chloride was prepared by drop-wise addition of acetyl chloride (1.40 mL, 20 mmol) to dry methanol (14 mL) at 0 °C, over 5 min, and the resulting solution was stirred for a further 30 min. A solution of **10a** (370 mg, 1.40 mmol) in methanol (1.0 mL) was then added drop-wise via syringe and the mixture was stirred at room temperature for 3 h. The mixture was then neutralized with solid $NaHCO_3$. Water was added, and the aqueous phase was extracted with dichloromethane (x 3) and the combined organic layers were washed with brine, dried (Na_2SO_4, then filtered) and the filtrate concentrated under reduced pressure to give

an amber oil. This oil was purified by flash chromatography (7 – 10% ethyl acetate/pentane) and carefully concentrated to give the volatile alcohol **S3**.[5]

Yield: 227 mg (95%). Isolated as a yellow oil as a solution in pentane (529 mg total, 52 wt%). To avoid mass-loss during further concentration, the mixture was typically used directly in the subsequent step. Analytical data are given for a >95% homogeneous sample.

R*f*: 0.33 in 10% ethyl acetate in pentane. Faintly UV active at very high concentrations and stains blue with CAM.

[α]D: − 26 (*c* = 1.00, CH_2Cl_2).

FT IR (Neat oil): 3381 (br.), 3045, 2949, 2936, 2656, 1452, 1375, 1363m 1323m 1288, 1188, 1155, 1094, 945, 730 cm^{-1}.

^{1}H NMR ($CDCl_3$, 400 MHz): δ 5.68 (dt, *J* = 5.8, 2.0 Hz, 1H), 5.65 (dt, *J* = 5.9, 2.0 Hz, 1H), 4.13 (dt, *J* = 8.6, 5.3 Hz, 1H), 2.53 (dt, *J* = 17.4, 2.0 Hz, 1H), 2.25 (dt, *J* = 17.4, 1.9 Hz, 1H), 2.03 - 2.10 (m, 1H), 1.99 (t, *J* = 8.9 Hz, 1H), 1.70 (d, *J* = 13.2 Hz, 1H), 1.62 (d, *J* = 13.2 Hz, 1H), 1.61 - 1.69 (m, 1H), 1.25 - 1.37 (m, 3H), 1.17 (s, 3H), 1.01 (s, 3H), 0.95 (s, 3H) ppm.

^{13}C NMR ($CDCl_3$, 101 MHz): δ 135.4, 129.6, 75.7, 73.7, 61.5, 58.0, 51.3, 49.1, 40.0, 36.5, 30.9, 27.2, 26.8, 24.0 ppm.

HRMS (ESI-QTOF) (*m/z*): A mass ion corresponding to the expected *m/z* could not be found. A dehydration adduct was observed: $[M + H - H_2O]^+$ Calcd for $C_{14}H_{21}$ 189.1643; Found: 189.1642.

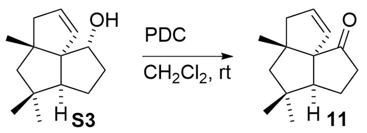

[5] It is recommended that the compound is concentrated carefully to near dryness after chromatography and used directly in the next step to avoid unnecessary loss of mass.

(4*S*,7*S*,8*S*,9*R*)-6,6,4-Trimethyl-9,10,11,7,6,5,4,3-hexahydrocyclopenta[c]pentalen-9-(2H)-one (11). To a stirred solution of **S3** (120 mg, 580 μmol) in dichloromethane (5.8 mL) was added pyridinium dichromate in one portion (1.10 g, 2.90 mmol) and the mixture was stirred in a sealed microwave tube for 20 h. The mixture was filtered through a plug of silica, then washed with 20% ether in pentane (100 mL) and concentrated to near dryness to give a yellow oil. The compound was purified by flash chromatography (2 – 30% ether/pentane) to give the highly volatile ketone **11**.[6]

Yield: 122 mg, (quant.). Isolated as a colourless solution in pentane (409 mg total, 30 wt%). To avoid mass-loss during further concentration, the mixture was typically used directly in the subsequent step. Analytical data are given for a >95% homogeneous sample.

R_f: 0.30 in 3% ethyl acetate in pentane. Faintly UV active and stains blue with CAM.

$[\alpha]_D$: −160 (c = 1.00 CH_2Cl_2)

FT IR (Neat oil): 3046, 2953, 2926, 2868, 1730 (s), 1463, 1408, 1385, 1376, 1366, 1294, 1189, 1157, 1135, 1022, 979, 913, 795, 732 cm^{-1}.

^{1}H NMR ($CDCl_3$, 400MHz): δ 5.81 (ddd, J = 5.7, 2.3, 2.2 Hz, 1H), 5.35 (ddd, J = 5.7, 2.3, 2.2 Hz, 1H), 2.52 (dt, J = 17.4, 2.3 Hz, 1H), 2.39 (dt, J = 17.4, 2.2 Hz, 1H), 2.28 - 2.36 (m, 1H), 2.24 (dd, J = 12.5, 8.6 Hz, 1H), 2.16 - 2.23 (m, 1H), 1.98 (dddd, J = 13.0, 9.0, 7.7, 1.6 Hz, 1H), 1.83 (d, J = 13.6 Hz, 1H), 1.73 (d, J = 13.6 Hz, 1H), 1.48 - 1.62 (m, 1H), 1.11 (s, 3H), 1.08 (s, 3H), 1.04 (s, 3H) ppm.

^{13}C NMR ($CDCl_3$, 101 MHz): δ 223.2, 134.7, 133.5, 78.2, 58.4, 56.8, 53.9, 52.4, 42.4, 39.7, 29.5, 29.0, 26.2, 23.5 ppm.

HRMS (ESI-QTOF) (*m/z*): $[M + H]^+$ Calcd for $C_{14}H_{21}O$ 205.1592; Found 205.1588.

[6]Ketone **11** is surprisingly volatile and partial concentration is recommended to avoid mass-loss during isolation.

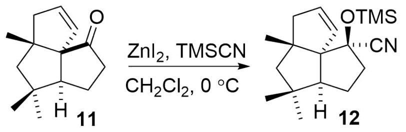

(4*S*,7*S*,8*S*,9*R*)-6,6,4-Trimethyl-9-((trimethylsilyl)oxy)-9,10,11,7,6,5,4,3-octahydrocyclo penta[c]pentalen-9-carbonitrile (12). To a stirred solution of **11** (150 mg, 750 µmol) in dichloromethane (7.5 mL) was added zinc iodide in one portion (179 mg, 561 µmol) at 0 °C. TMS cyanide (0.14 mL, 560 µmol) was added drop-wise via syringe and the mixture was stirred under an argon atmosphere for 1 h. Brine (4 mL) was added and the mixture was shaken vigorously until the pink coloration faded from the organic phase, then filtered immediately through a phase separator, the aqueous phase washed with dichloromethane (20 mL) and the solution concentrated to partial dryness to give a yellow oil. The compound was purified by flash chromatography on 'deactivated silica' (3% ether/pentane) to give the cyanohydrin **12**.

Yield: 135 mg (59%). Isolated as a viscous yellow oil. >95% pure by NMR spectroscopy and one spot by TLC.

R*f*: 0.33 in 3% Et_2O in pentane. UV inactive and stains blue with CAM

[α]D: − 60 (*c* = 1.00 CH_2Cl_2).

FT IR (Neat oil): 3050, 2954, 2912, 2869, 1459, 1446, 1364, 1301, 1252, 1181, 1150, 1083, 1062, 1052, 999, 976, 938, 901, 867, 841, 755, 705, 634 cm^{-1}.

^{1}H NMR ($CDCl_3$, 400 MHz): δ 5.71 (dt, *J* = 5.9, 2.3 Hz, 1H), 5.66 (dt, *J* = 6.0, 2.0 Hz, 1H), 2.42 (ddd, *J* = 16.7, 2.3, 2.0 Hz, 1H), 2.24 (ddd, *J* = 16.8, 2.3, 2.0 Hz, 1H), 2.17 (t, *J* = 7.6 Hz, 3H), 1.77 - 1.89 (m, 2H), 1.75 (d, *J* = 13.2 Hz, 1H), 1.69 (d, *J* = 13.2 Hz, 1H), 1.32 (s, 3H), 1.05 (s, 3H), 1.00 (s, 3H), 0.24 (s, 9H) ppm.

^{13}C NMR ($CDCl_3$, 101 MHz): δ 137.3, 130.2, 121.8, 80.1, 77.4, 59.5, 59.4, 52.5, 50.7, 41.0, 39.9, 33.4, 27.6, 25.5, 23.2, 1.3 ppm.

HRMS (ESI-QTOF) (*m/z*): $[M + Na]^+$ Calcd for $C_{18}H_{29}NNaOSi$ 326.1916; Found 326.1919.

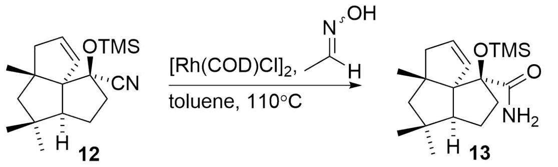

(4*S*,7*S*,8*S*,9*R*)-6,6,4-Trimethyl-9-((trimethylsilyl)oxy)-9,10,11,7,6,5,4,3-octahydrocyclo penta[c]pentalen-9-carboxamide (**13**). To a solution of chloro(1,5-cyclooctadiene)rhodium (I) dimer (35 mg, 71 μmol) in toluene (70 mL) was added a solution of cyanohydrin **12** (210 mg, 700 μmol) in toluene (2 mL) in one portion via syringe and the resulting solution heated to 100 °C. A solution of acetaldehyde oxime (0.43 mL, 7.10 mmol) in toluene (20 mL) was added via syringe pump over 24 h. After cooling, water was added, the aqueous phase extracted with dichloromethane (x 3), and the combined organic layers were dried (Na_2SO_4, then filtered). The filtrate was concentrated under reduced pressure to give a red/brown oil which was purified by flash chromatography (5% ether/pentane to 100% ethyl acetate) to give alpha-hydroxyamide **13** along with a second fraction contacting cyanohydrin **12** (85 mg, 79% total yield BRSM).

Yield: 107 mg (47%). Isolated as a viscous yellow oil, >95% pure by NMR spectroscopy and one spot by TLC.

R*f*: 0.11 in 30% Et_2O/pentane. UV inactive and stains blue with CAM.

[α]D: − 66 (*c* = 1.00, CH_2Cl_2).

FT IR (Neat oil): 3495, 3332, 3277, 3206, 3156, 3049, 2952, 2923, 2868, 2843, 1683 (s), 1583, 1450, 1373, 1308, 125, 1207, 1179, 1168, 1152, 1140, 1120, 1096, 1040, 1012, 994, 974, 942, 905, 877, 841 (s), 775, 724, 688, 630 cm^{-1}.

^{1}H NMR ($CDCl_3$, 400 MHz): δ 6.06 (br. s, 1H), 5.57 (ddd, *J* = 5.9, 2.6, 1.9 Hz, 1H), 5.55 (br.s, 1H), 5.47 (ddd, *J* = 5.9, 2.3, 1.4 Hz, 1H), 2.44 (dt, *J* = 13.0, 8.6 Hz, 1H), 2.29 (ddd, *J* = 16.2,

2.3, 1.9 Hz, 1H), 2.19 (t, *J* = 9.4 Hz, 1H), 2.17 (ddd, *J* = 16.2, 2.6, 1.4 Hz, 1H) 1.95 (ddd, *J* = 13.6, 8.8, 4.6 Hz, 1H), 1.77 - 1.86 (m, 2H), 1.72 (d, *J* = 13.3 Hz, 1H), 1.68 (d, *J* = 13.3 Hz, 1H), 1.43 (s, 3H), 1.03 (s, 3H), 0.95 (s, 3H), 0.13 - 0.19 (s, 9 H) ppm.

^{13}C NMR ($CDCl_3$, 101 MHz): δ 178.6, 139.0, 128.1, 91.1, 75.7, 61.5, 59.5, 52.9, 50.6, 38.6, 36.1, 32.8, 27.6, 27.3, 24.8, 1.9.

HRMS (ESI-QTOF) (*m/z*): $[M + H]^+$ Calcd for $C_{18}H_{32}NO_2Si$ 322.2202; Found 322.2209.

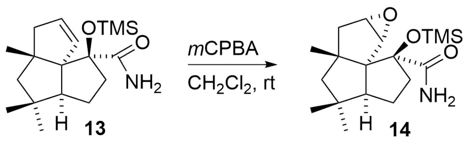

(1*S*,2*S*,4*R*,7*S*,8*S*,9*R*)-6,6,4-Trimethyl-9-((trimethylsilyl)oxy)decahydrocyclopenta[3,2] pentaleno[1,2-b]-9-carboxamide (14). To a stirred solution of amide **13** (54 mg, 170 μmol) in dichloromethane (4.2 mL) was added *meta*-chloroperbenzoic acid (290 mg, 1.70 mmol) in one portion and the was mixture stirred at room temperature for 5 h. Sodium bisulfite (sat. aq.) was then added and the aqueous phase extracted with dichloromethane (x 3). The combined organic layers were washed with $NaHCO_3$ (sat. aq.), brine, dried (Na_2SO_4, then filtered) and the filtrate concentrated under reduced pressure. The resulting yellow oil was purified by flash chromatography (50% ether/pentane to 100% ethyl acetate) to give the epoxide **14** as a single detected diastereomer.

Yield: 43 mg (75%). Isolated as a colorless solid, >95% pure by NMR spectroscopy and one spot by TLC.

R_f: 0.07 in 60% Et_2O/pentane. UV inactive and stains blue with CAM

$[\alpha]_D$: − 19 (*c* = 0.23, CH_2Cl_2).

FT IR (Neat solid): 3505, 3344, 4215, 2951, 2875, 1681 (s), 1587, 1456, 1402, 1383, 1364, 1327, 1298, 1253, 1216, 1183, 1142, 1122, 1072, 1061, 1001, 977, 933, 906, 878 (s), 842, 754, 682, 625 cm^{-1}.

^{1}H NMR ($CDCl_3$, 400 MHz): δ 6.30 (br. s, 1H), 5.63 (br. s. 1H), 3.41 (dd, *J* = 2.6, 1.8 Hz, 1H), 3.39 (d, *J* = 2.6 Hz, 1H), 2.56 - 2.68 (m, 1H), 2.16 (dd, *J* = 15.1, 1.8 Hz, 1H), 2.11 (d, *J* = 9.0 Hz, 1H), 1.90 - 1.96 (m, 1H), 1.88 (d, *J* = 15.1 Hz, 1H), 1.76 - 1.84 (m, 2H), 1.74 (d, *J* = 12.9 Hz, 1H), 1.54 (d, *J* = 12.9 Hz, 1H), 1.32 (s, 3H), 1.13 (s, 3H), 0.98 (s, 3H), 0.23 (s, 9H) ppm.

^{13}C NMR ($CDCl_3$, 101 MHz): δ 176.9, 89.9, 73.2, 66.2, 59.6, 59.3, 56.4, 50.4, 44.7, 39.1, 38.7, 34.2, 28.9, 28.9, 25.1, 2.3 ppm.

HRMS (ESI-QTOF) (*m/z*): $[M + Na]^+$ Calcd for $C_{18}H_{31}NNaO_3Si$ 360.1971; Found 350.1966.

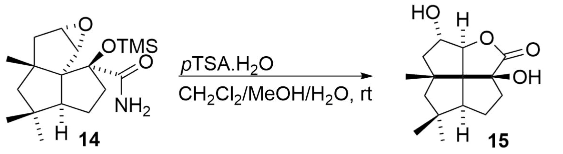

(1*S*,2*S*,4*R*,7*S*,8*S*,9*S*)-2,9-Dihydroxy-6,6,4-trimethyldecahydro-2H-1-oxapentaleno[1,6-cd]pentalen-15-one (15). To a solution of epoxide **14** (42 mg, 120 μmol) in a mixture of methanol (4.0 mL), dichloromethane (4.0 mL), and water (0.3 mL) was added *para*-toluene sulfonic acid monohydrate (240 mg, 1.30 mmol) in one portion. The resulting mixture was stirred for 3 days and then neutralized with $NaHCO_3$ (sat. aq.). The aqueous phase was extracted with dichloromethane (x 3) and then the combined organic layers were dried (Na_2SO_4), passed through a phase separator, and the filtrate concentrated under reduced pressure to give a viscous yellow oil that was purified by flash chromatography (50% ether/pentane to 100% ethyl acetate) to give the alcohol **15**.

Yield: 24 mg (72%). Isolated as a semi-amorphous solid, 95% pure by NMR spectroscopy and a single spot by TLC.

R*f*: 0.04 in 50% Et_2O/pentane. UV inactive and stains blue with CAM

[α]D: − 16 (c = 0.27, CH_2Cl_2).

FT IR (Neat solid): 3450 (br), 2950, 2868, 1772(s), 1732, 1459, 1446, 1435, 1387, 1365, 1278, 1235, 1208, 1178, 1113, 1080, 1059, 1001, 955, 026, 899, 858, 814, 758, 732, 726, 668, 627, 590 cm^{-1}.

^{1}H NMR ($CDCl_3$, 400 MHz): δ 4.34 - 4.41 (m, 2H), 3.01 (s, 1H), 2.25 (ddd, J = 13.5, 11.3, 7.8 Hz, 1H), 2.25 (t, J = 8.3 Hz, 1H), 2.10 (ddd, J = 13.4, 7.1, 2.6, 1H),, 2.00 - 2.06 (m, 1H), 1.92 - 2.00 (m, 1H), 1.78 - 1.85 (m, 1H), 1.77 (s, 1H), 1.75 (s, 1H), 1.70 (dd, J = 13.6, 7.8 Hz, 1H), 1.38 (s, 3H), 1.09 (s, 3H), 1.01 (s, 3H) ppm. A chemical shift corresponding to the rapidly exchanging tertiary alcohol was not found.

^{13}C NMR ($CDCl_3$, 101 MHz): δ 179.0, 90.9, 85.2, 72.4, 71.7, 62.8, 60.8, 48.9, 48.6, 42.0, 40.8, 31.0, 28.6, 25.6, 24.9 ppm.

HRMS (ESI-QTOF) (*m/z*): $[M + Na]^+$ Calcd for $C_{15}H_{22}NaO_4$ 289.1415; Found 289.1411.

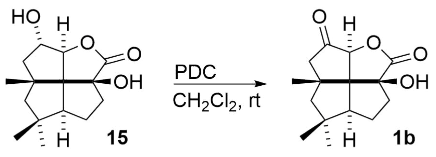

(*9S,8S,7S,4R,1S*)-9-Hydroxy-6,6,4-trimethyloctahydro-2H-1-oxapentaleno[1,6-cd] pentalene-2,15(1H)-dione (1b). To a stirred solution of alcohol **15** (21 mg, 79 µmol) in dichloromethane (4.0 mL) was added pyridinium dichromate (300 mg, 780 µmol) in one portion and the mixture was stirred for 17 h. The mixture was filtered through a plug of silica, which was washed with ether (50 mL). The filtrate was concentrated under reduced pressure to give ketone **1b**.

Yield: 21 mg (quant.). Isolated as a colorless semi-crystalline solid, >95% pure by NMR spectroscopy and a single spot by TLC.

MP: 96-98 °C.

R_f: 0.04 in 50% Et_2O/pentane. UV inactive and stains blue with CAM.

$[\alpha]_D$: − 30 (c = 0.47, CH_2Cl_2).

FT IR (Neat solid): 3544 (br.), 2953, 2924, 2869, 2854, 1774 (s), 1759 (s), 1459, 1379, 1367, 1282, 1260, 1234, 1205, 1171, 1148, 1123, 1089, 1003, 972, 890, 844, 802, 743 cm^{-1}.

^{1}H NMR ($CDCl_3$, 400 MHz): δ 4.26 (dd, J = 0.9, 0.6 Hz, 1H), 2.67 (dd, J = 17.5, 0.6 Hz, 1H), 2.66 (br. S, 1H), 2.49 - 2.58 (m, 1H), 2.39 (dd, J = 17.6, 0.9 Hz, 1H), 2.13 - 2.30 (m, 3H), 1.96 - 2.04 (m, 1H), 1.92 (d, J = 13.8 Hz, 1H), 1.84 (d, J = 13.8 Hz, 1H), 1.35 (s, 3H), 1.16 (s, 3H), 1.15 (s, 3H) ppm.

^{13}C NMR ($CDCl_3$, 101 MHz): δ 208.5, 177.8, 85.5, 85.0, 71.5, 61.8, 57.9, 53.7, 46.1, 43.0, 39.8, 33.9, 27.3, 26.6, 25.1 ppm.

HRMS (ESI-QTOF) (*m/z*): $[M + H]^+$ Calcd for $C_{15}H_{21}O_4$ 265.1440; Found 265.1443.

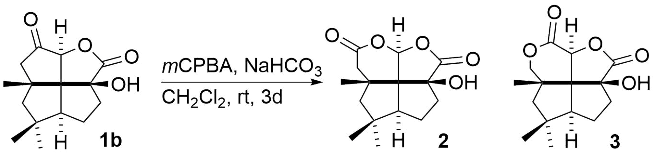

Penifulvin D (2). To a solution of ketone **1b** (5 mg, 20 μmol) and sodium bicarbonate (38 mg, 450 μmol) in dichloromethane (4 mL) was added *meta*-chloroperbenzoic acid (25 mg, 150 μmol) in one portion and the mixture was stirred for 3 days. Sodium bisulfite (sat. aq.) was then added and the aqueous phase extracted with dichloromethane (x 3) and the combined organic layers were washed with $NaHCO_3$ (sat. aq), dried (Na_2SO_4, then filtered), and the filtrate concentrated under reduced pressure to give a 95 : 5 mixture of the penifulvin D (**2**) and

asperaculin A (**3**) as shown by ^{1}H NMR spectroscopy of the reaction crude. The mixture was purified with flash chromatography (40 – 100% ether/pentane) to give penifulvin D (**2**).

Yield: 2.3 mg (46%). Isolated as an amorphous colorless solid, >95% pure by NMR spectroscopy and a single spot by TLC.

R*f*: 0.26 in 60% Et_2O/pentane. UV inactive and stains blue with CAM.

[α]D: − 89 (*c* = 0.14, CH_2Cl_2).

FT IR (Neat Solid): 3461 (br.), 2960, 2934, 2874, 1799 (s), 1770 (s), 1465, 1390, 1368, 1307, 1231, 1205, 1187, 1123, 1078, 1034, 1012, 989, 933(s) cm^{-1}.

^{1}H NMR ($CDCl_3$, 500 MHz): δ 5.94 (s, 1H), 2.74 (s, 1H), 2.67 (dq, *J* = 15.4, 0.8 Hz, 1H), 2.45 (d, *J* = 15.4 Hz, 1H), 2.38 - 2.48 (m, 2H), 2.15 (ddd, *J* = 14.4, 10.2, 7.1, 1H), 2.04 (d, *J* = 13.8 Hz, 1H), 1.97 - 2.08 (m, 1H), 1.81 (dtd, *J* = 14.4, 9.6, 7.1 Hz, 1H), 1.74 (d, *J* = 13.8 Hz, 1H), 1.36 (d, *J* = 0.8 Hz, 3H), 1.20 (s, 3H), 1.10 (s, 3H) ppm.

^{13}C NMR ($CDCl_3$, 125 MHz): δ 176.8, 167.7, 103.1, 84.7, 66.4, 59.8, 56.7, 44.5, 42.8, 39.7, 39.2, 33.2, 27.1, 25.1, 24.2 ppm.

HRMS (ESI-QTOF) (*m/z*): $[M + H]^+$ Calcd for $C_{15}H_{21}O_5$ 303.1208; Found 303.1205.

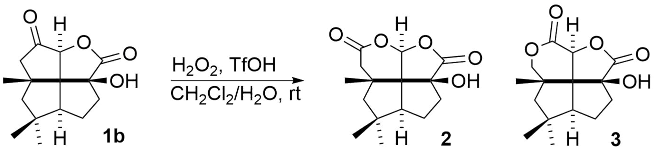

Asperaculin A (3). To a solution of ketone **1b** (5 mg, 20 μmol) in dichloromethane (5.0 mL) was added 50% aqueous hydrogen peroxide (0.10 mL, 1.80 mmol), followed by a solution of triflic acid in dichloromethane (3.8 M, 0.50 mL). The resulting mixture was stirred for 2 min, after which sodium bisulfite was added. The aqueous phase was extracted with dichloromethane (x 3) and the combined organic layers were washed with $NaHCO_3$ (sat. aq.),

dried (Na_2SO_4 then filtered) and the filtrate concentrated under reduced pressure to give a yellow oil containing a 77 : 23 mixture of asperaculin A (**3**) and penifulvin D (**2**) as measured by ^{1}H NMR spectroscopy. The mixture was purified by flash chromatography (40 – 100% Et_2O/pentane) to give asperaculin A (**3**).

Yield: 2.6 mg (52%). Isolated as a semi-amorphous colorless solid, >95% purity by NMR spectroscopy and a single spot by TLC.

R*f*: 0.19 in 60% Et_2O/pentane. UV inactive but stains blue with CAM.

[α]D: − 4 (*c* = 0.17, MeOH).

FT IR (Neat Solid): 3460 (br.), 2967, 2928, 2873, 1781, 1661, 1649, 1454, 1378, 1353, 1289, 1209, 1177, 1152, 1139, 1121, 1076, 1051, 1026, 963, 872, 829, 757 cm^{-1}.

^{1}H NMR ($CDCl_3$, 500 MHz): δ 4.92 (s, 1H), 4.44 (d, *J* = 11.7 Hz, 1H), 3.95 (d, *J* = 11.7 Hz, 1H), 2.57 (s, 1H), 2.47 (ddd, *J* = 14.3, 9.3, 4.9 Hz, 1H), 2.30 (dd, *J* = 9.5, 7.1 Hz, 1H), 2.00 - 2.08 (m, 1H), 1.95 (d, *J* = 14.3 Hz, 2H), 1.89 - 1.99 (m, 1H), 1.75 - 1.86 (m, 1H), 1.64 (d, *J* = 14.3 Hz, 1H), 1.40 (s, 3H), 1.23 (s, 3H), 1.08 (s, 3H) ppm.

^{13}C NMR ($CDCl_3$, 125 MHz): δ 177.3, 167.9, 84.4, 76.7, 75.8, 65.6, 60.7, 52.6, 45.3, 40.5, 35.7, 32.3, 27.2, 24.3, 23.6 ppm.

HRMS (ESI-QTOF) (*m/z*): $[M + Na]^+$ Calcd for $C_{15}H_{20}NaO_5$ 303.1208; Found 303.1201.

*III. Comparison of synthetic and natural **2** and **3**.*

Penifulvin D

	Synthetic sample	*Natural sample*[7]
Specific rotation $[\alpha]_D$:	− 89 (c = 0.14, CH_2Cl_2)	− 60 (c = 0.3, CH_2Cl_2)

NMR spectroscopy: Chemical shifts are referenced to residual $CHCl_3$, ^1H δ = 7.24; ^{13}C δ = 77.23 for both samples.

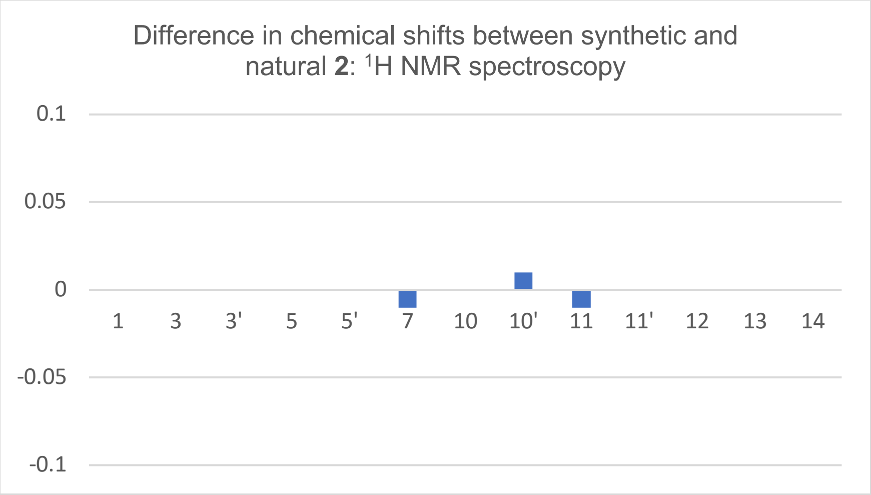

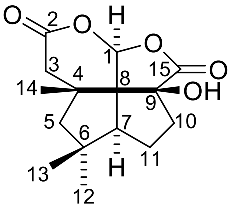

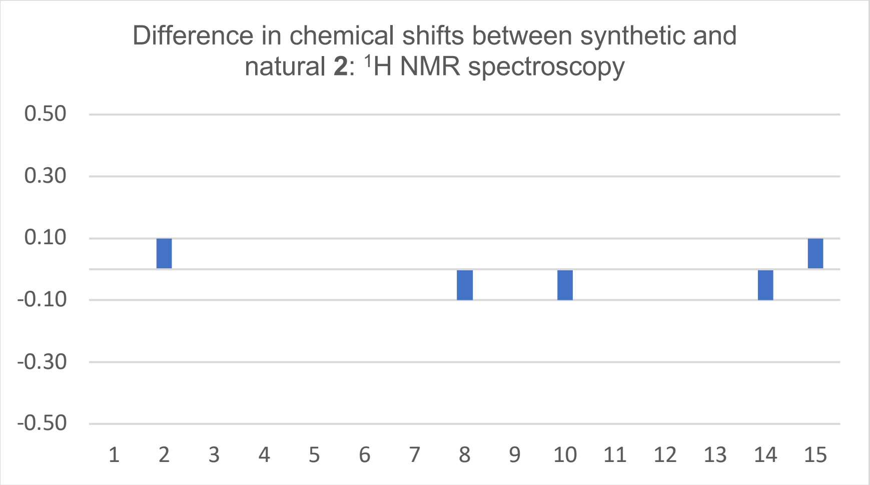

Figure S6. Differences in chemical shifts between synthetic and natural penifulvin D (**2**)

[7] Shim, A; Gloer, J; Wicklow, D.; "Penifulvins B-E and a Silphinene Analogue: Sesquiterpenoids from a Fungicolous Isolate of Penicillium griseofulvum" *J. Nat. Prod.* **2006**, 69, 1601-1605.

Asperaculin A

	Synthetic sample	*Natural sample*[8]
Specific rotation $[\alpha]_D$:	− 4 (*c* = 0.14, MeOH)	− 10 (*c* = 0.23, MeOH)

NMR spectroscopy data: No references were given for the chemical shifts of residual $CDCl_3$ in the line listing of natural material.[9] To facilitate comparison, chemical shifts were normalized to have a zero difference for *H*(C1) and C1 in the ^{1}H NMR and ^{13}C NMR spectroscopy data respectively.

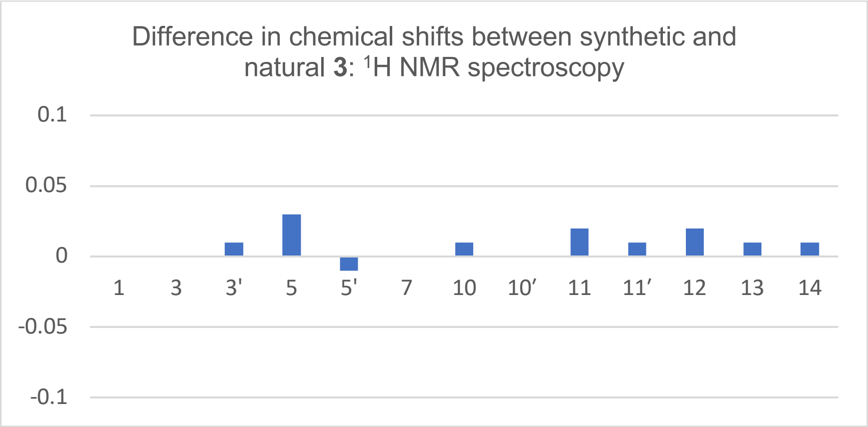

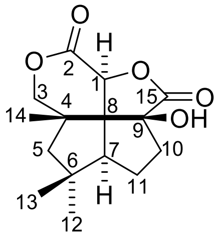

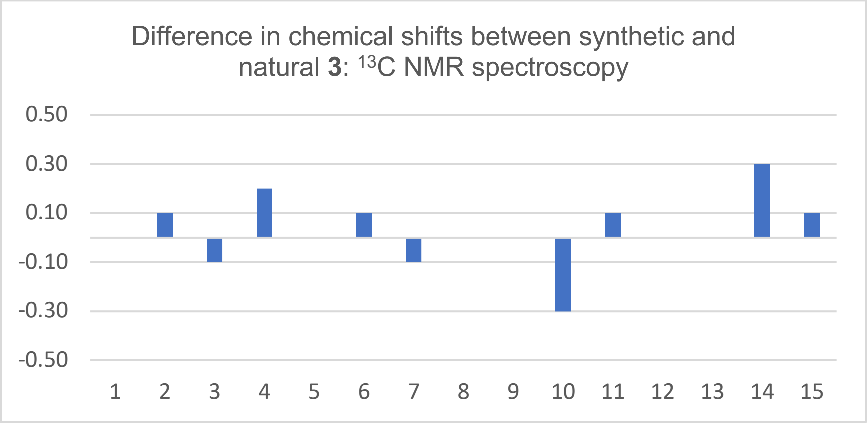

Figure S7. Difference in chemical shifts between synthetic and natural asperaculin A (**3**)

---

[8] Shim, A; Gloer, J; Wicklow, D. Penifulvins B-E and a Silphinene Analogue: Sesquiterpenoids from a Fungicolous Isolate of Penicillium griseofulvum. *J. Nat. Prod.* **2006**, 69, 1601-1605.

[9] Ingavat, N.; Mahidol, C; Ruchirawat, S; Kittakoop, P. Asperaculin A, a Sesquiterpenoid form a Marine-Derived Fungus, Aspergillus aceleatus. *J. Nat. Prod.* **2011**, 74, 1650-1652.

IV. *Single crystal X-ray diffraction (scXRD) analysis of **2** and **3***

Single crystals covered in paratone oil were cut to size and mounted on a MiTeGen micro-mount loop. For low temperature data-collection, the mounted crystals were rapidly transferred to the nitrogen cold stream of the diffractometer. Data collection was performed on an Agilent Xcalibur Sapphire3 or an Agilent Enhance diffractometer equipped with a MoKα high-brilliance IμS radiation source (λ = 0.71073 Å) and an Oxford Cryosystems low temperature device. Absorption was corrected for using multi-scan empirical absorption correction with spherical harmonics as implemented in the SCALE3 ABSPACK scaling algorithm.[10] The structures were solved in WinGX[11] using SUPERFLIP[12] or SHELXL 2016/4[13] and refined using SHELXL 2016/4. Non-hydrogen atoms were refined anisotropically.

scXRD data for (–)-penifulvin D. *Crystallization:* A solution of **2** (<1 mg in Et_2O ~0.2 mL) was placed in a sealed diffusion chamber filled with hexane (~2.0 mL) at room temperature. After 12 hours single crystals suitable for X-ray analysis had formed.

Table S1. Crystal data for (–)-penifulvin D[a]

Chemical formula	$C_{15}H_{20}O_5$
Formula weight	280.31
Collection temperature /K	110(2)
Crystal size /mm^3	0.3 x 0.2 x 0.1
Crystal habit	colorless, irregular
Wavelength /Å	0.71073
Crystal system	orthorhombic
Space group	$P2_12_12_1$
Unit cell dimensions:	a = 8.5687(3) Å α = 90 °
	b = 9.4746(3) Å β = 90 °
	c = 17.1908(6) Å γ = 90 °
Unit cell volume /Å^3	1395.64(8)
Z, Calculated density /Mg/m^3	4, 1.334
Absorption coefficient /mm^{-1}	0.100

[10] CrysAlis PRO. Agilent Technologies 2011.
[11] Farrugia, L. J. WinGX and ORTEP for Windows : An Update. *J. Appl. Crystallogr.* **2012**, *45* (4), 849–854. https://doi.org/10.1107/S0021889812029111.
[12] Palatinus, L.; Chapuis, G. SUPERFLIP – a Computer Program for the Solution of Crystal Structures by Charge Flipping in Arbitrary Dimensions. *J. Appl. Crystallogr.* **2007**, *40* (4), 786–790. https://doi.org/10.1107/S0021889807029238.
[13] Sheldrick, G. M. A Short History of SHELX. *Acta Crystallogr. Sect. A Found. Crystallogr.* **2008**, *64* (1), 112–122. https://doi.org/10.1107/S0108767307043930.

Radiation type	MoKα
Theta range for data collection /º	3.418 to 29.277
Limiting indices	-11 ≤ h ≤ 11, -12 ≤ k ≤ 12, -22 ≤ l ≤ 22
Reflections collected / unique	15532 / 3264
R_{int}	0.0480
Completeness to theta =	25.000 /% 99.6
Refinement method	Full-matrix least-squares on F^2
Data / restraints / parameters	3264 / 0 / 185
Goodness of fit on F^2	1.031
Final R indices $(I > 2\sigma(I))$	R_1 = 0.0441, wR_2 = 0.0928
R indices *(all data)*	R_1 = 0.0531, wR_2 = 0.0980
Largest diff. peak and hole /e$^-$/Å^3	0.257 and -0.193
CCDC	2042599

[a]The absolute configuration was assigned by reference to unchanging stereogenic centers in the synthetic sequence.

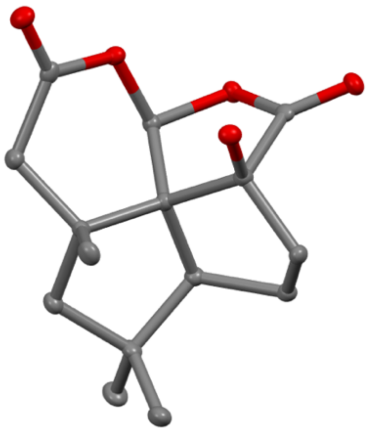

Figure S1. The asymmetric unit of (–)-penifulvin D (**2**). Black = carbon atom; red = oxygen atom. Thermal ellipsoids shown at 30% probability. Hydrogen atoms are omitted for clarity.

scXRD data for (–)-asperaculin A. *Crystallization:* A solution of **3** (<1 mg in Et_2O ~0.2 mL) was placed in a sealed diffusion chamber filled with hexane (~2.0 mL) at room temperature. After 12 hours single crystals suitable for X-ray analysis had formed.

Table S2. Crystal data for (–)-asperaculin A (**3**)[a]

Chemical formula	$C_{15}H_{20}O_5$
Formula weight	280.31
Collection temperature /K	293(2)
Crystal size /mm^3	0.15 x 0.15 x 0.05
Crystal habit	colorless, irregular
Wavelength /Å	0.71073
Crystal system	orthorhombic
Space group	$P2_12_12_1$
Unit cell dimensions:	a = 9.0556(8) Å α = 90 °
	b = 10.5449(9) Å β = 90 °
	c = 14.3884(13) Å γ = 90 °
Unit cell volume /Å^3	1374.0(2)
Z, Calculated density /Mg/m^3	4, 1.355

Absorption coefficient /mm^{-1}	0.101
Radiation type	MoKα
Theta range for data collection /°	3.428 to 29.721
Limiting indices	$-11 \le h \le 11$, $-13 \le k \le 12$, $-18 \le l \le 19$
Reflections collected / unique	6599 / 3204
R_{int}	0.0412
Completeness to theta =	25.000 /% 99.6
Refinement method	Full-matrix least-squares on F^2
Data / restraints / parameters	3204 / 0 / 185
Goodness of fit on F^2	1.027
Final R indices $(I > 2\sigma(I))$	$R_1 = 0.0722$, $wR_2 = 0.0984$
R indices *(all data)*	$R_1 = 0.1418$, $wR_2 = 0.1183$
Largest diff. peak and hole /e$^-$/Å^3	0.170 and -0.153
CCDC	2042600

[a]The absolute configuration was assigned by reference to unchanging stereogenic centers in the synthetic sequence.

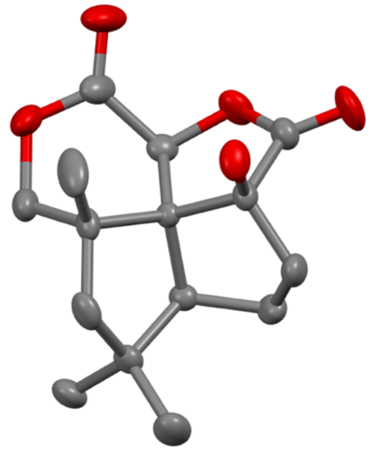

Figure S2. The asymmetric unit of (–)-asperaculin A (**3**). Black = carbon atom; red = oxygen atom. Thermal ellipsoids shown at 30% probability. Hydrogen atoms are omitted for clarity.

*V. Optimization and kinetics of meta-photocycloaddition of **8***

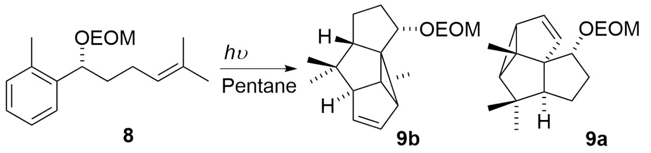

Arene-olefin **8** (80 mg, 310 μmol) was dissolved in pentane (10 mL) in a Herasil quartz vessel. The vessel was capped and degassed as described above. The sample was irradiated in a Rayonet RPR-100 photoreactor at the specified frequency. At the stated time points the tube was removed and an aliquot (0.50 mL) was drawn. The remaining mixture was degassed, and irradiation continued. The aliquot was carefully concentrated (700 mbar at 40 °C) and then 1-

methoxynaphthalene (25 µL, 170 µmol) was added as an internal standard together with $CDCl_3$. The sample was homogenized and the amounts of **8**, **9a**, and **9b** were quantified by ^{1}H NMR spectroscopy.

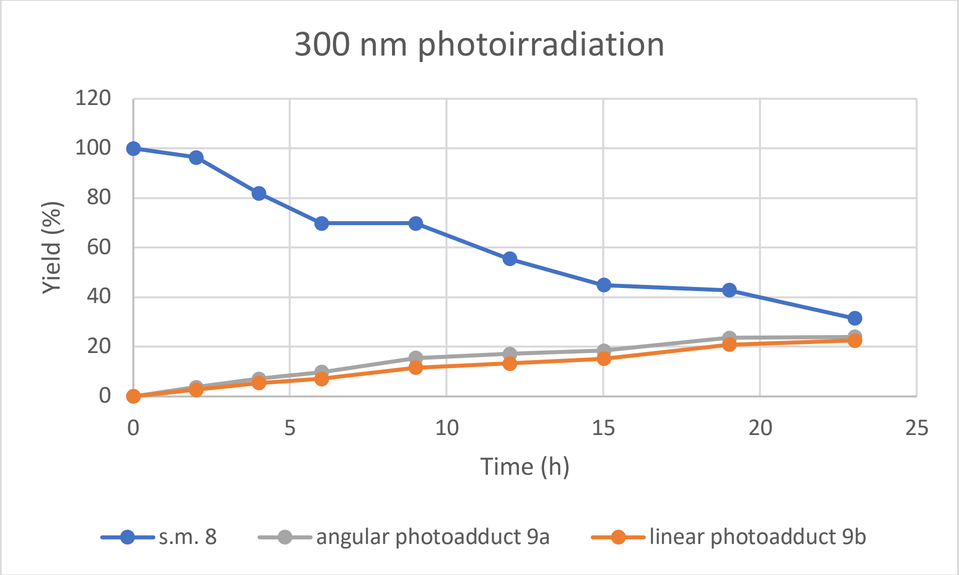

Figure S3. Photoirradiation of **8** at 254 nm. Yields are expressed in % of theoretical.

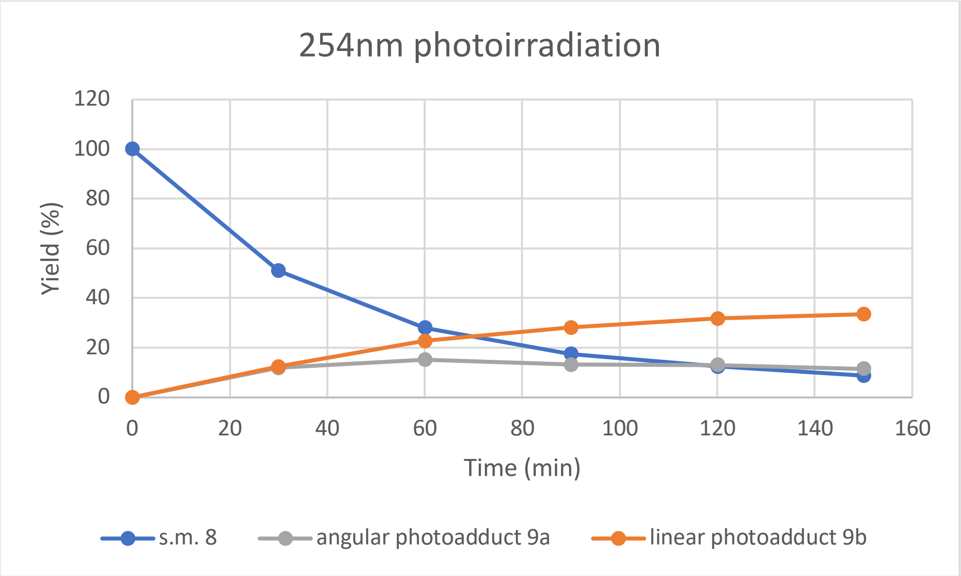

Figure S4. Photoirradiation of **8** at 300nm. Yields are expressed in % of theoretical.

VI. *Photoisomerization of **9b** to **9a***

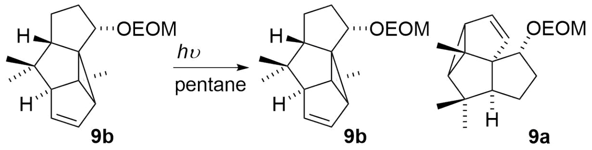

Linear cyclopropane **9b** (20 mg, 76 μmol) was dissolved in pentane (2.5 mL) in a quartz vessel. The sample was irradiated in a Rayonet RPR-100 photoreactor at the specified frequency. At the stated time points the mixture was concentrated and analysed by ^{1}H NMR spectroscopy. The mixture was then re-dissolved in pentane, degassed, and subjected to irradiation. Further irradiation beyond 72h at 300nm or 2h at 254 nm led to significant decomposition/by-product formation.

Table S3. Isomerization of **9b** to **9a**

Frequency	Time (h)	9b : 9a
254 nm	1	86 : 14
254 nm	2	83 : 17
300 nm	72	76 : 24

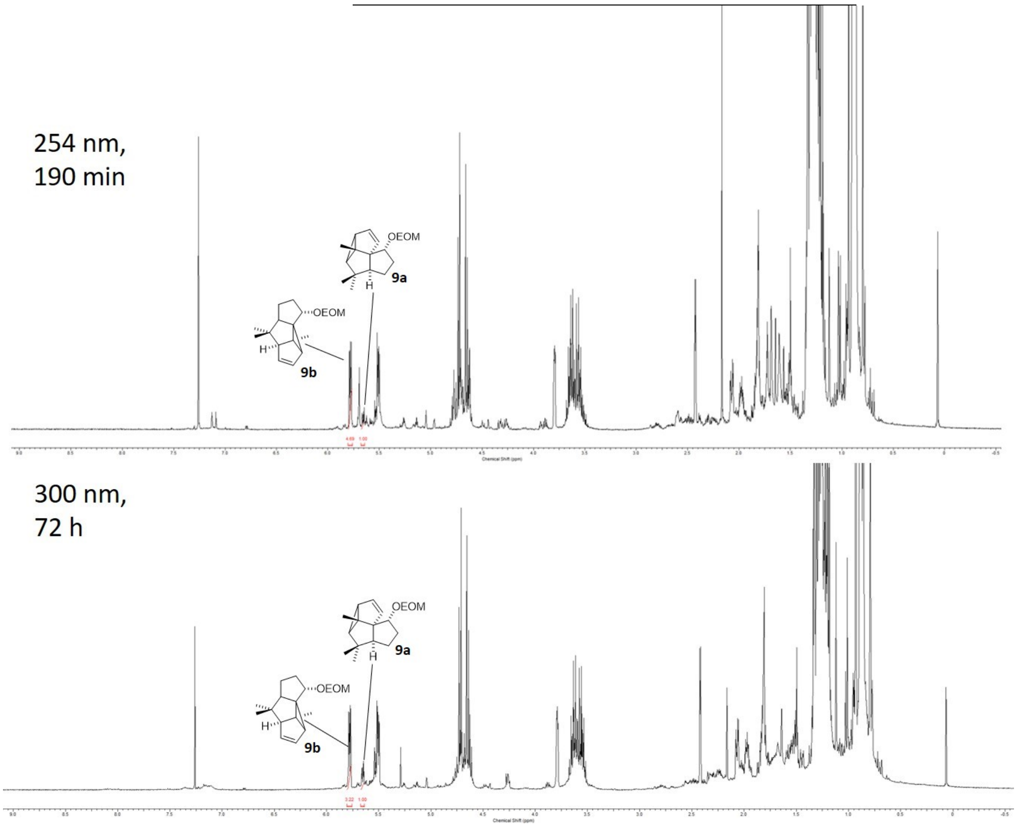

Figure S5. Photoisomerization studies of linear cyclopropane **9b**.

VII. Density functional theory calculations

All QM calculations were performed with Jaguar as implemented in Schrödinger release 2020-1,[14] using default settings except that the SCF accuracy level was set to accurate with a switch to analytical integrals near convergence. Solution phase energy minima and transition state geometries were determined employing density functional theory (DFT) with the m06-2x-d3 functional and the 6-31G** basis set using the Poisson-Boltzmann solvation model (PBF) with chloroform solvent parameters.[15] Vibrational frequencies were calculated for all stationary points to obtain the Gibbs Free Energies. No negative eigenvalues were found for minima and only one for saddle points when any negative frequencies smaller than -83 cm^{-1} were ignored. The relative Gibbs Free Energies at 298.15 K were calculated for all stationary points by application of isodesmic reactions. Total free energies are given at 298.15 K and 1.0 atm. Thick dashed bonds signify bonds that are formed / broken in TSs. Structure are visualized using CYLview 2.0.[16] Hydrogen atoms attached to carbon atoms are omitted for clarity. Dotted bonds signify weaker interactions. Atom coordinates of each calculated species are given in the calc_structures.pdb file available for download as supporting information.

[14] Schrödinger Release 2020-1: Schrödinger, LLC, New York, NY, 2020.

[15] Bochevarov, A. D.; Harder, E.; Hughes, T. F.; Greenwood, Jeremy R.; Braden, D. A.; Philipp, D. M.; Rinaldo, D.; Halls, M. D.; Zhang, J.; Friesner, R. A. Jaguar: A high-performance quantum chemistry software program with strengths in life and materials sciences. *Int. J. Quantum Chem.* **2013**, *113*, 2110-2142.

[16] CYLview20; Legault, C. Y., Université de Sherbrooke, 2020 (http://www.cylview.org).

Structures for the Baeyer-Villager reaction: peracid pathway:

AcOO–Criegee intermediate towards penifulvin D (**CI$_{pen}$**)

Gibbs free energy: -1187.994479 a.u.

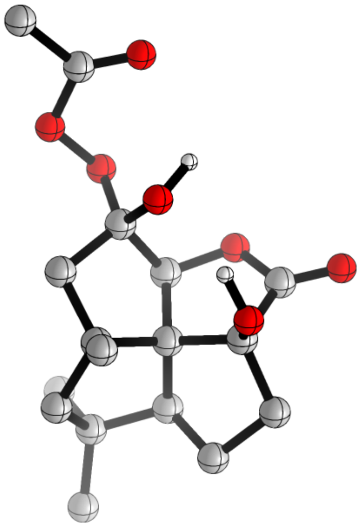

Transition state of the AcOOH reaction to penifulvin D (**TS$_{pen}$**)

Gibbs free energy: -1187.948678 a.u.

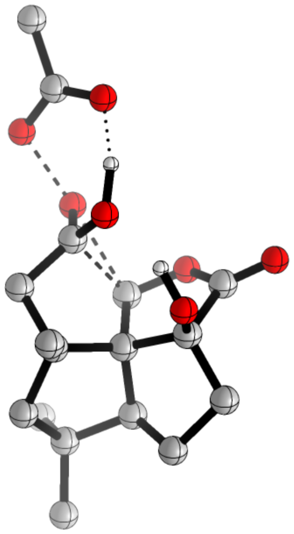

Penifulvin D – AcOH

Gibbs free energy: -1188.10343 a.u.

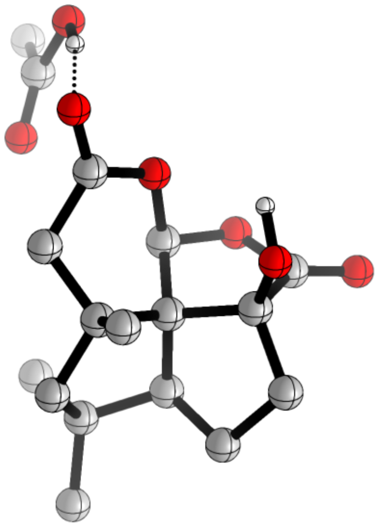

AcOO–Criegee intermediate towards asperaculin A (**CI$_{asp}$**)

Gibbs free energy: -1187.995611 a.u.

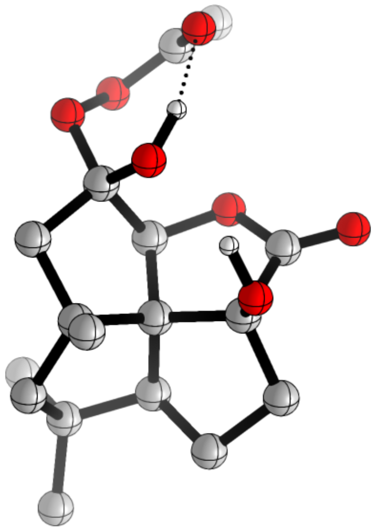

Transition state of the AcOOH reaction to asperaculin A (**TS$_{asp}$**)

Gibbs free energy: -1187.94558 a.u.

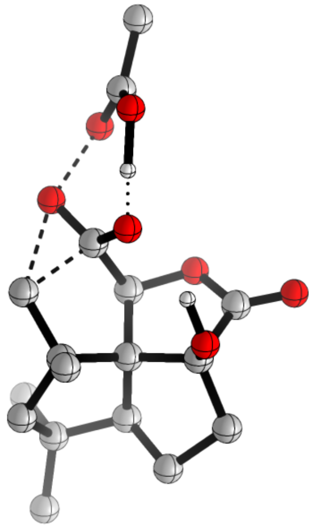

Asperaculin A – AcOH

Gibbs free energy: -1188.093872 a.u.

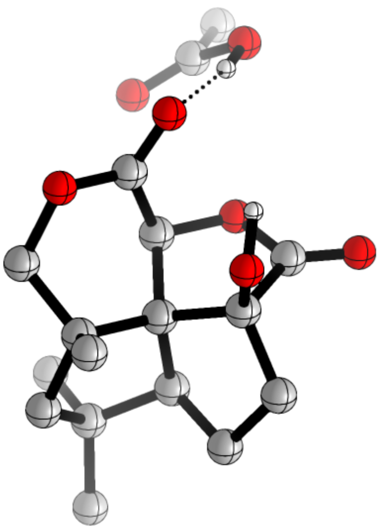

Structures for the Baeyer-Villager reaction: hydrogen peroxide pathway,

H_2O_2/H_3O^+–Criegee intermediate towards penifulvin D (**CI_{pen}**)

Gibbs free energy: -1112.196569 a.u.

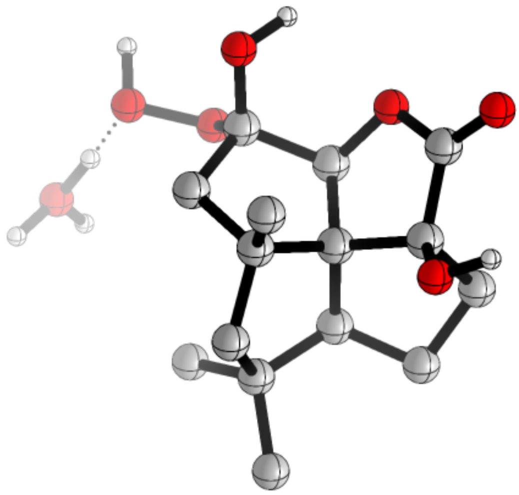

Transition state of the H_2O_2/H_3O^+ reaction to penifulvin D (**TS_{pen}**)

Gibbs free energy: -1112.162904 a.u.

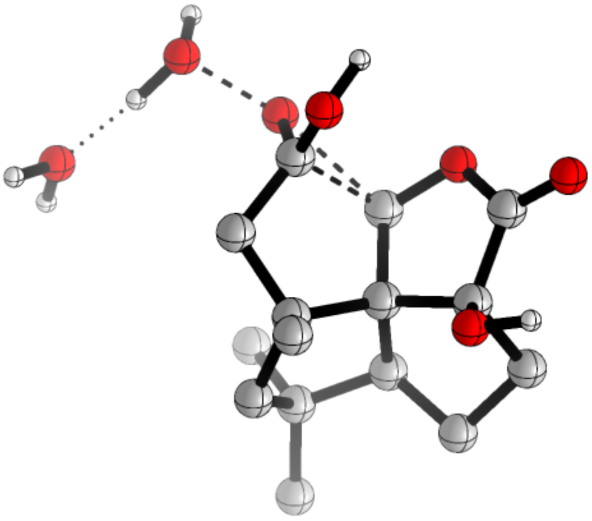

Penifulvin D

Gibbs free energy: -959.145155 a.u.

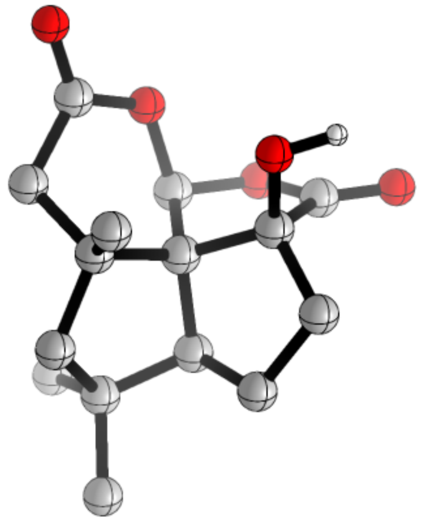

H_2O_2/H_3O^+

Gibbs free energy: -153.188822 a.u.

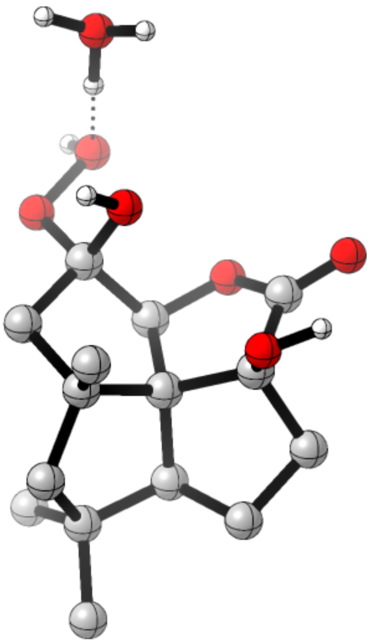

H_2O_2/H_3O^+–Criegee intermediate towards asperaculin A (**CI_{asp}**)

Gibbs free energy: -1112.198447 a.u.

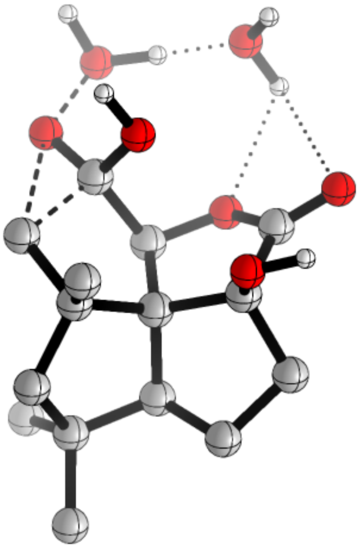

Transition state of the H_2O_2/H_3O^+ reaction to asperaculin A (**TS_{asp}**)

Gibbs free energy: -1112.167261 a.u.

Asperaculin A

Gibbs free energy: -959.137457 a.u.

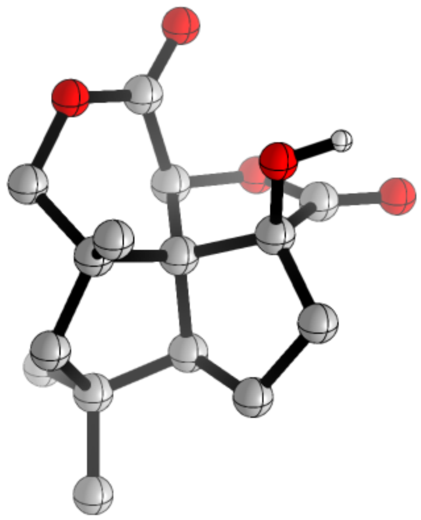

*VII. Comparison of heat of formation between model acetal **S4** and bis-lactone **S5***

To gain further insight into the origin of the difference in free energy between asperaculin A and penifulvin D we used 1,4-dioxane-2,5-dione (**S4**) and 1,3-dioxane-4,6-dione (**S5**) as simplified models capturing differences between the key acetal/bis-lactone motif. The gas phase energies were calculated using the Jaguar workflow for Heat of Formation, employing the DFT/M06-2x functional and the 6-311g-3df-3pd++ basis set, both for geometry optimizations and for energy calculations. The results show that the acetal is thermodynamically favored over the corresponding bis-lactone motif by 9.8 kcal/mol at 298K. For atomic coordinates of **S4** and **S5** in .xyz format, see the accompanying file.

Table S4. Comparison of gas phase energy between isomers **S4** and **S5**

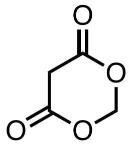

S4

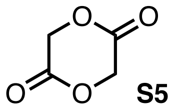

S5

Atomization energy (0K):	1287.75 kcal/mol	Atomization energy (0K):	1277.74 kcal/mol
Atomization energy (298K):	1292.34 kcal/mol	Atomization energy (298K):	1282.51 kcal/mol
ΔH of formation (0K):	-165.35 kcal/mol	ΔH of formation (0K):	-155.34 kcal/mol
ΔH of formation (298K):	-169.94 kcal/mol	ΔH of formation (298K):	-160.11 kcal/mol

*VIII. Copies of ^{1}H and ^{13}C NMR spectra for compounds **6**, **8**, **9a**, **10a**, **10b**, **S3**, **11-15**, **1b**, **2**, and **3***

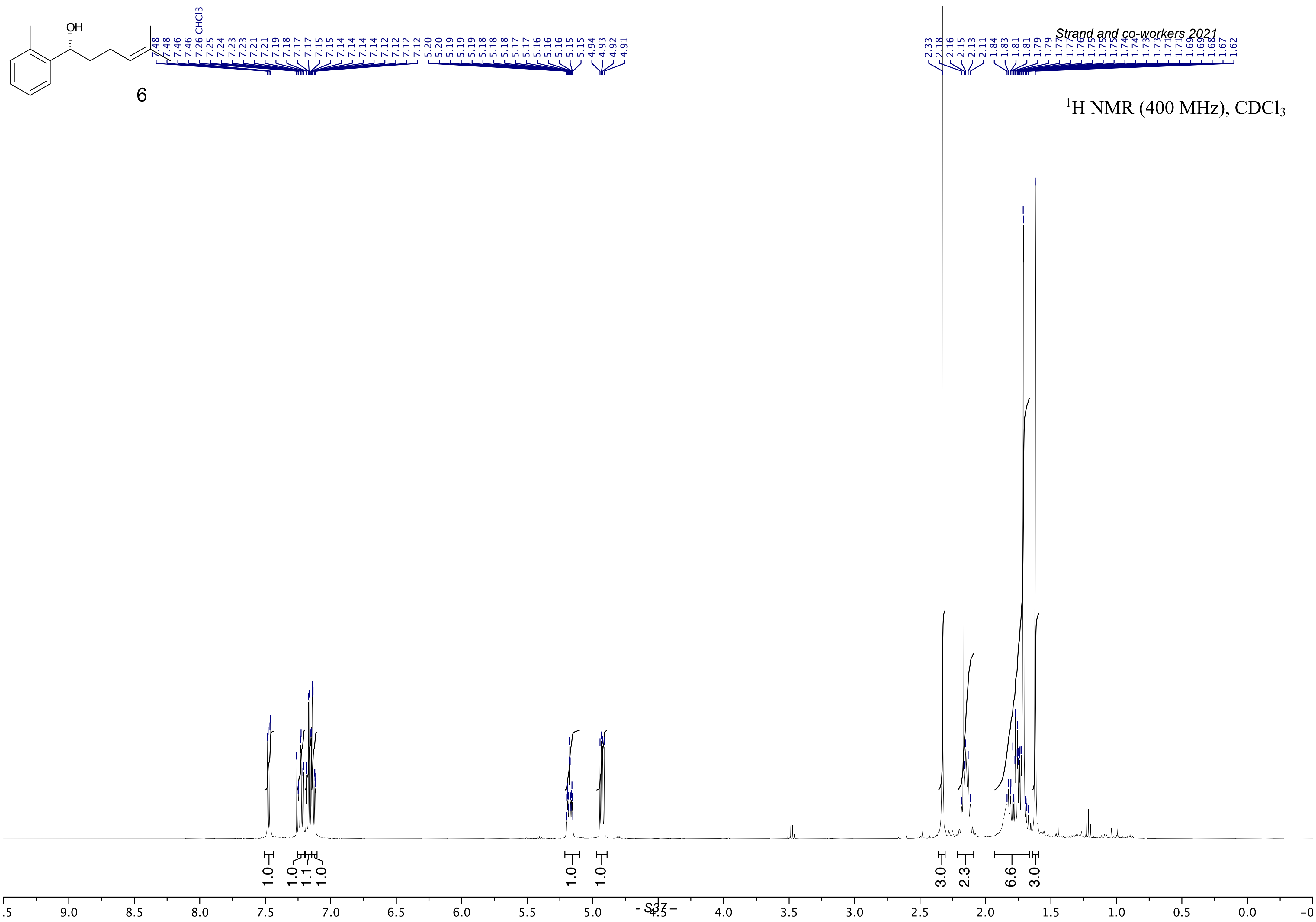

OH
6
7.48 7.48 7.46 7.46 7.26 CHCl3 7.25 7.24 7.23 7.23 7.21 7.21 7.19 7.18 7.17 7.17 7.15 7.15 7.14 7.14 7.14 7.14 7.12 7.12 7.12 7.12
5.20 5.20 5.19 5.19 5.19 5.18 5.18 5.18 5.17 5.17 5.16 5.16 5.16 5.15 5.15
4.94 4.93 4.92 4.91
2.33 2.18 2.16 2.15 2.13 2.11
1.84 1.83 1.81 1.81 1.79 1.79 1.77 1.77 1.76 1.75 1.75 1.75 1.74 1.74 1.73 1.73 1.71 1.71 1.69 1.69 1.68 1.67 1.62
Strand and co-workers 2021
^{1}H NMR (400 MHz), CDCl$_3$
1.0 1.0 1.1 1.0 1.0 1.0 3.0 2.3 6.6 3.0
.5 9.0 8.5 8.0 7.5 7.0 6.5 6.0 5.5 5.0 4.5 4.0 3.5 3.0 2.5 2.0 1.5 1.0 0.5 0.0 -0

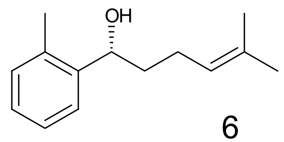

6

Strand and co-workers 2021

^{13}C NMR (101 MHz), $CDCl_3$

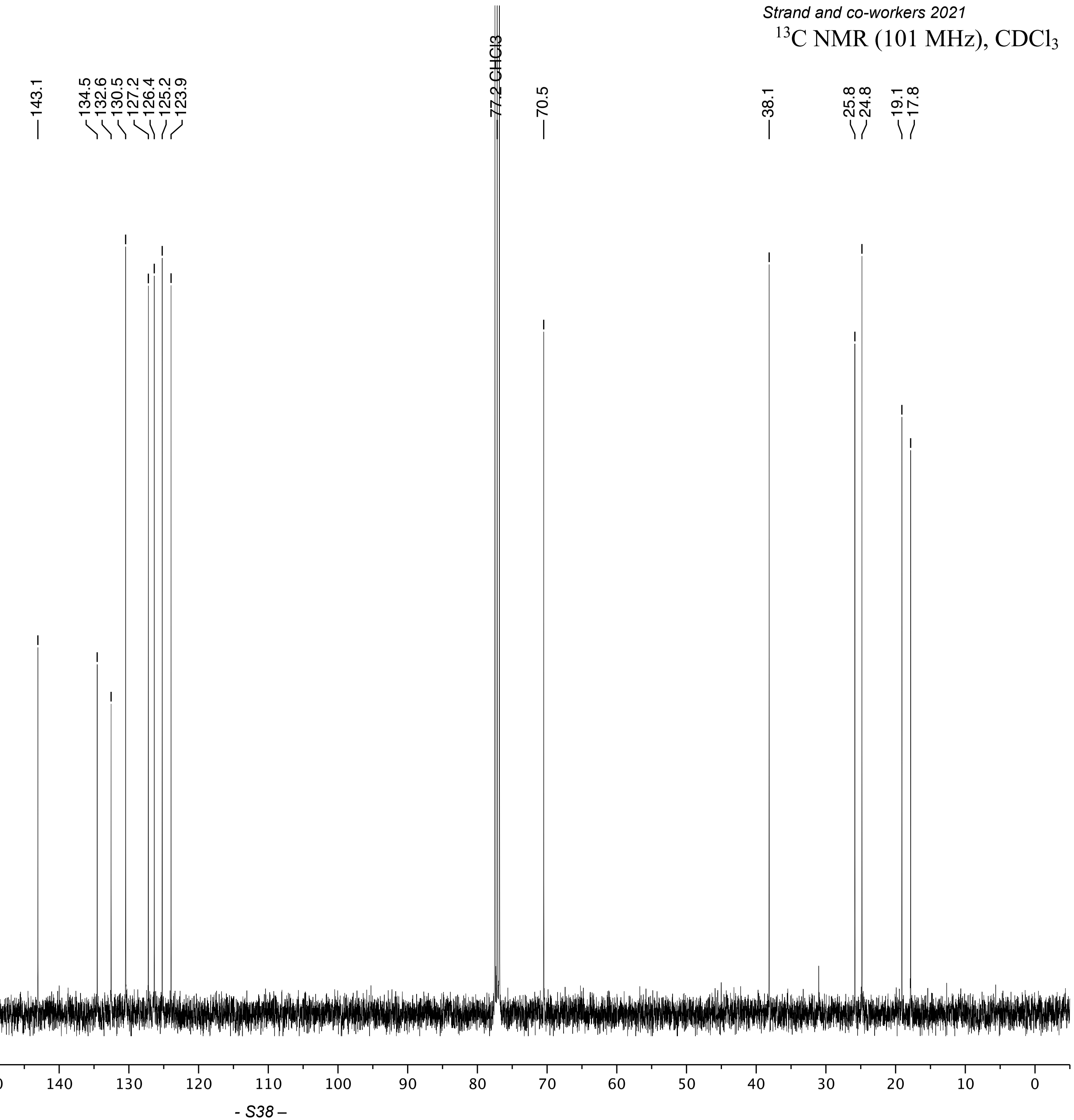

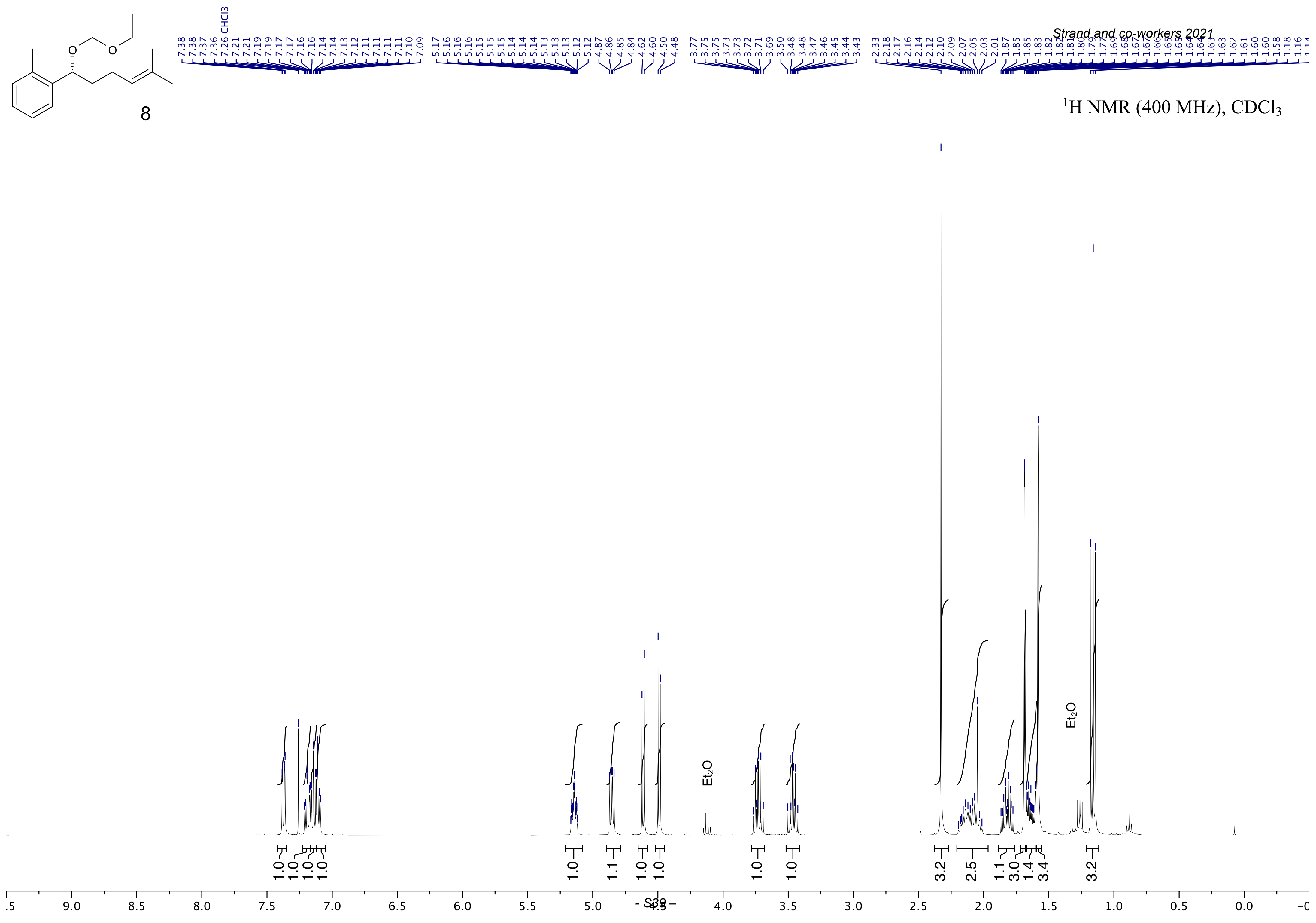

8
Strand and co-workers 2021
1H NMR (400 MHz), CDCl3
7.38
7.38
7.37
7.36
7.26 CHCl3
7.21
7.21
7.19
7.19
7.17
7.17
7.16
7.16
7.14
7.14
7.13
7.12
7.11
7.11
7.11
7.11
7.10
7.09
5.17
5.16
5.16
5.16
5.15
5.15
5.15
5.14
5.14
5.14
5.13
5.13
5.13
5.12
5.12
4.87
4.86
4.85
4.84
4.62
4.60
4.50
4.48
3.77
3.75
3.75
3.73
3.73
3.72
3.71
3.69
3.50
3.48
3.48
3.47
3.46
3.45
3.44
3.43
2.33
2.18
2.17
2.16
2.14
2.12
2.10
2.09
2.07
2.05
2.03
2.01
1.87
1.85
1.85
1.83
1.82
1.82
1.81
1.80
1.79
1.77
1.69
1.68
1.67
1.67
1.66
1.65
1.65
1.64
1.64
1.63
1.63
1.62
1.61
1.60
1.60
1.58
1.18
1.16
Et2O
Et2O
1.0
1.0
1.0
1.0
1.0
1.1
1.0
1.0
1.0
1.0
3.2
2.5
1.1
3.0
1.4
3.4
3.2
9.0
8.5
8.0
7.5
7.0
6.5
6.0
5.5
5.0
4.5
4.0
3.5
3.0
2.5
2.0
1.5
1.0
0.5
0.0

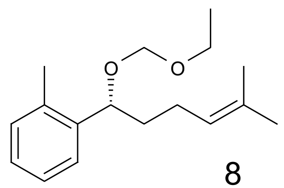

8

Strand and co-workers 2021

^{13}C NMR (101 MHz), $CDCl_3$

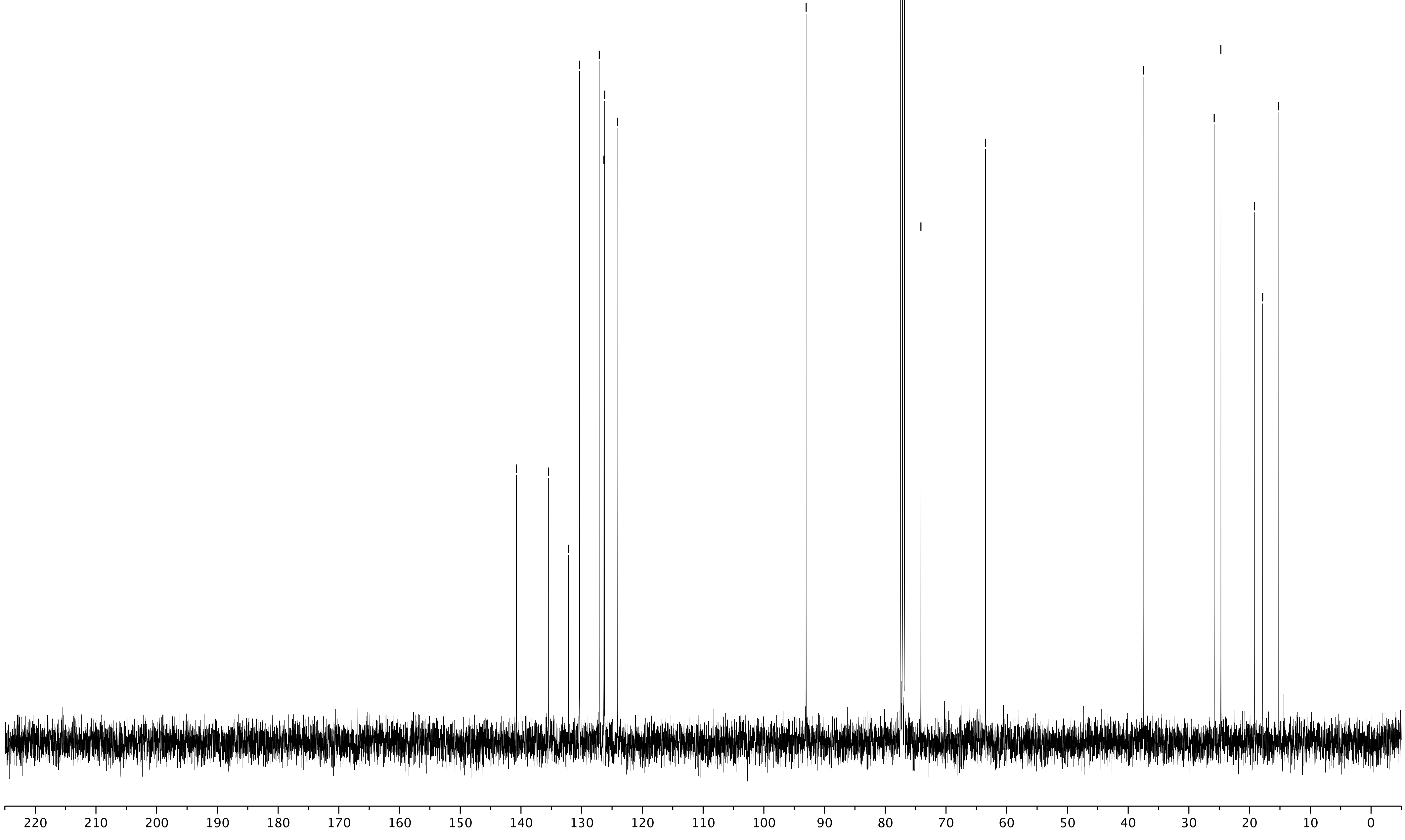

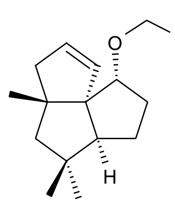

10a

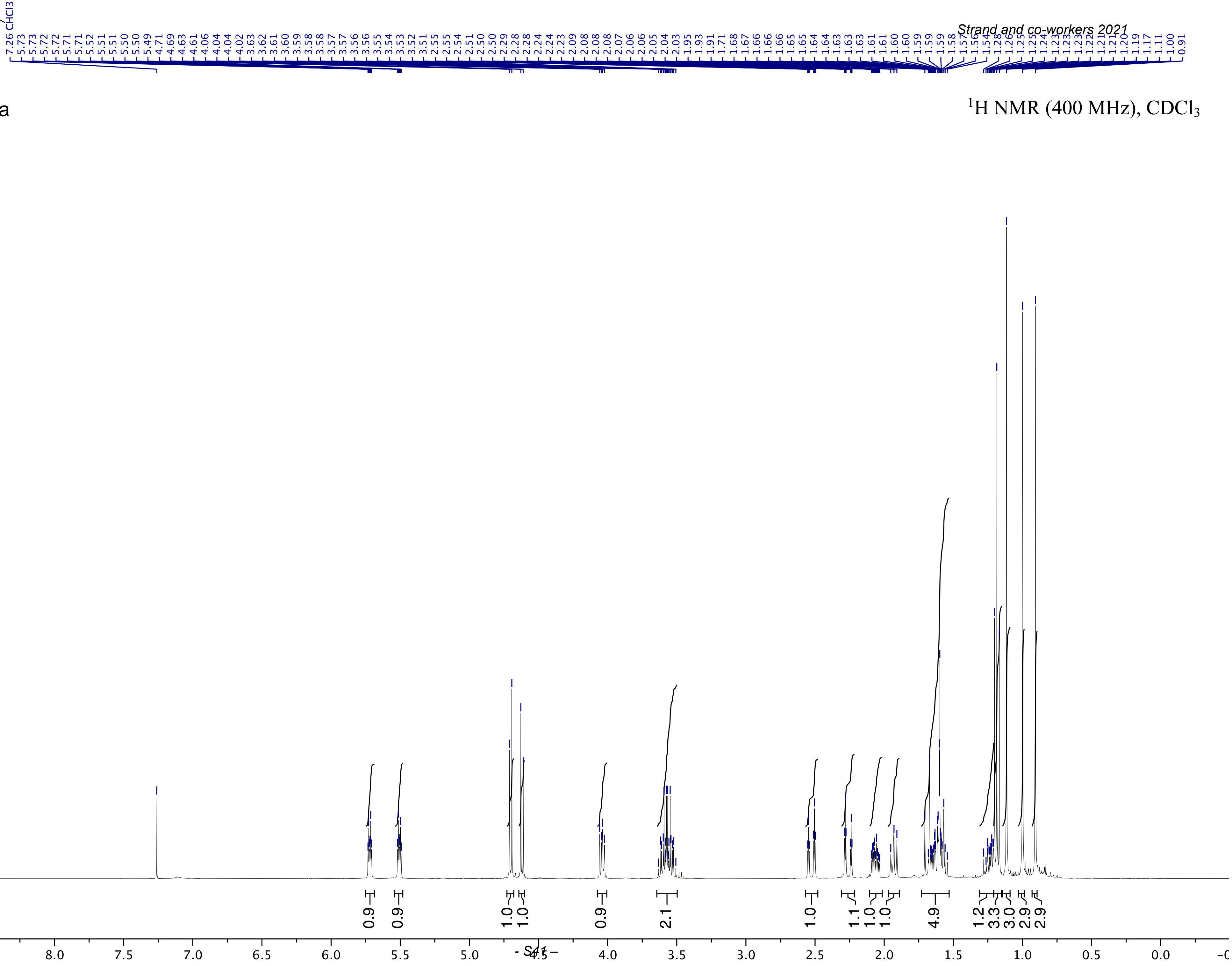

^{1}H NMR (400 MHz), $CDCl_3$

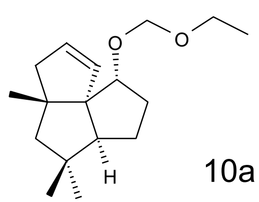

10a

Strand and co-workers 2021

^{1}H NMR (400 MHz), $CDCl_3$

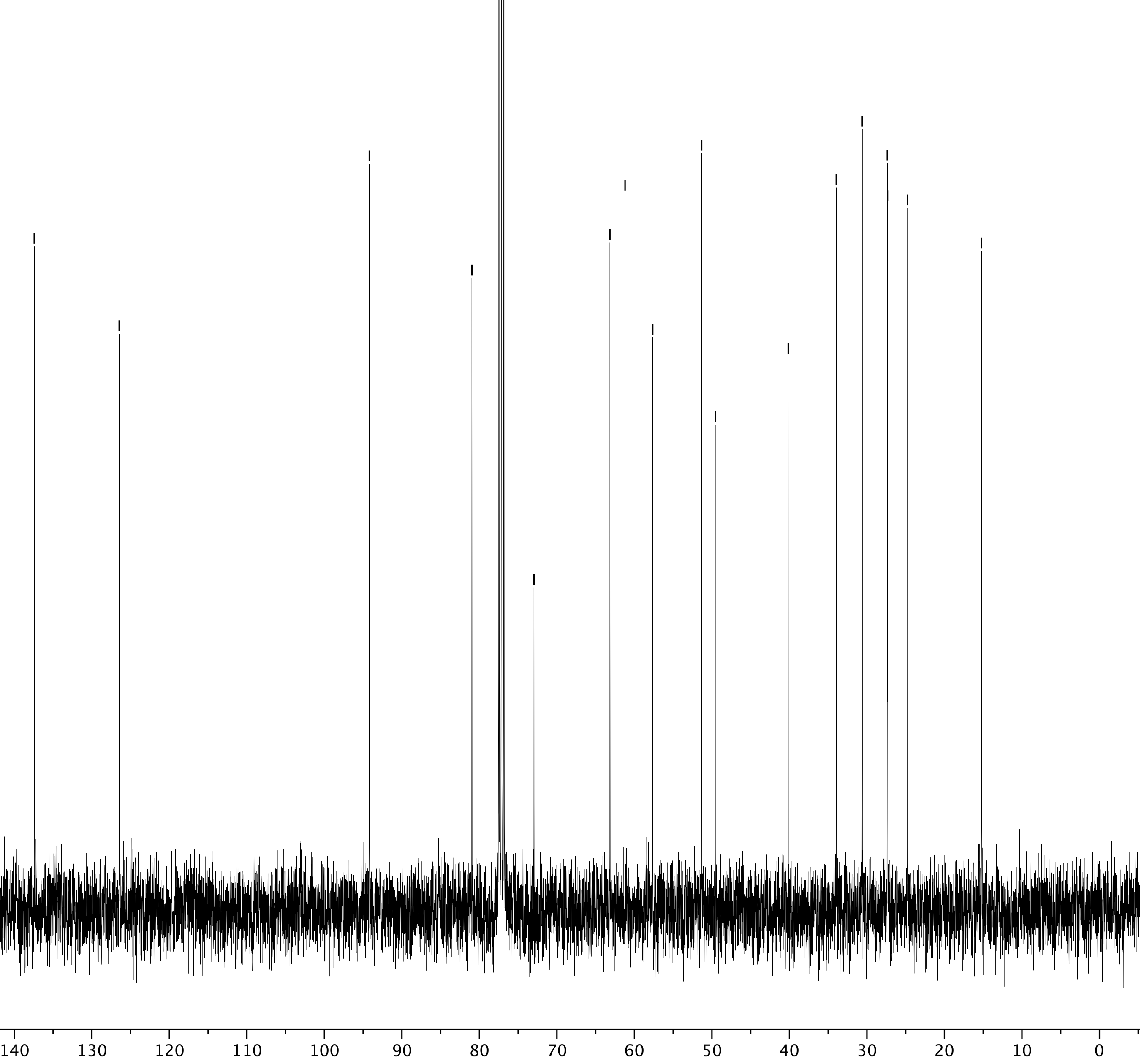

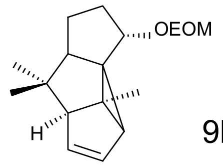

9b

^{1}H NMR (400 MHz), $CDCl_3$

Strand and co-workers 2021

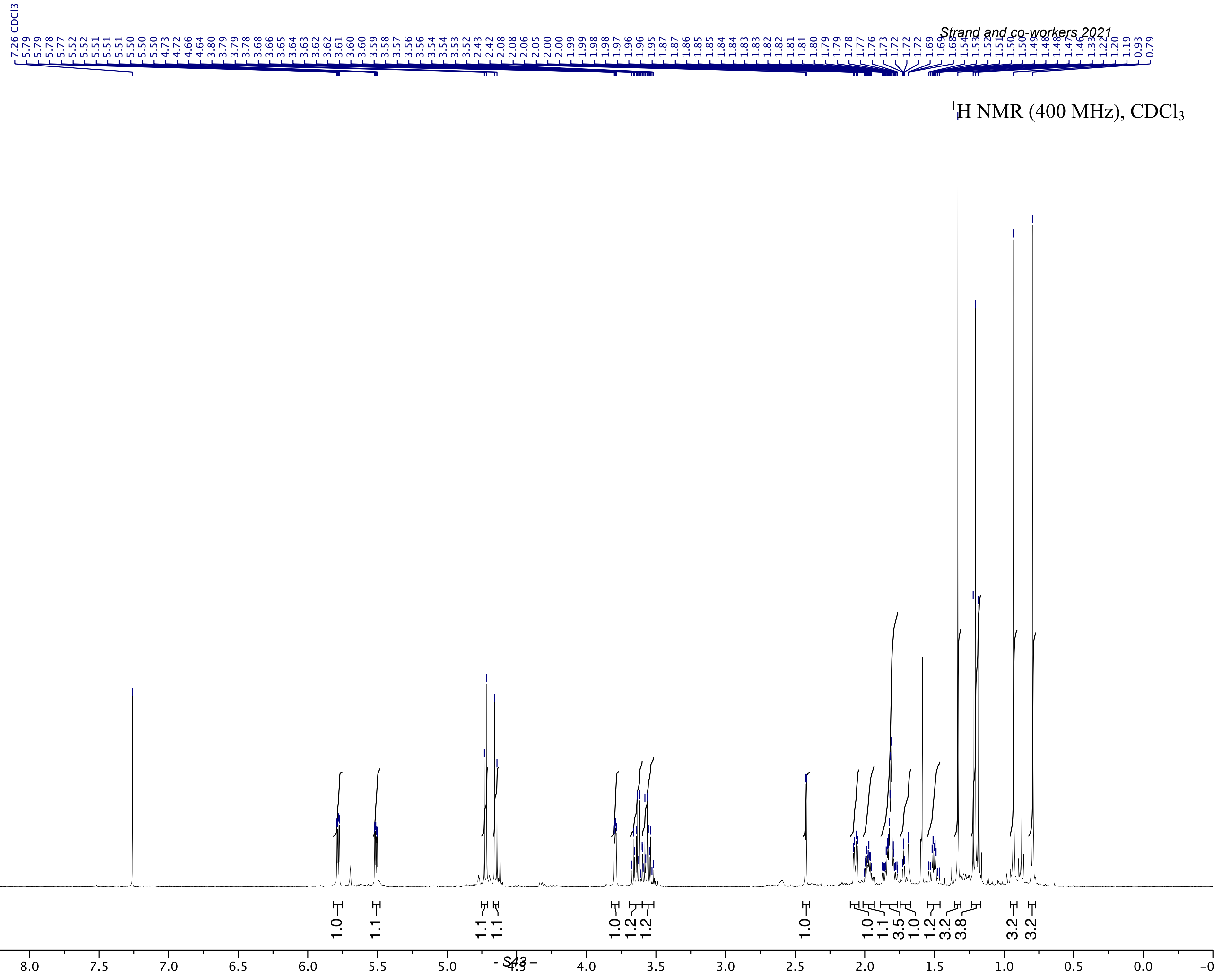

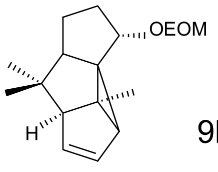

9b

Strand and co-workers 2021

^{13}C NMR (101 MHz), $CDCl_3$

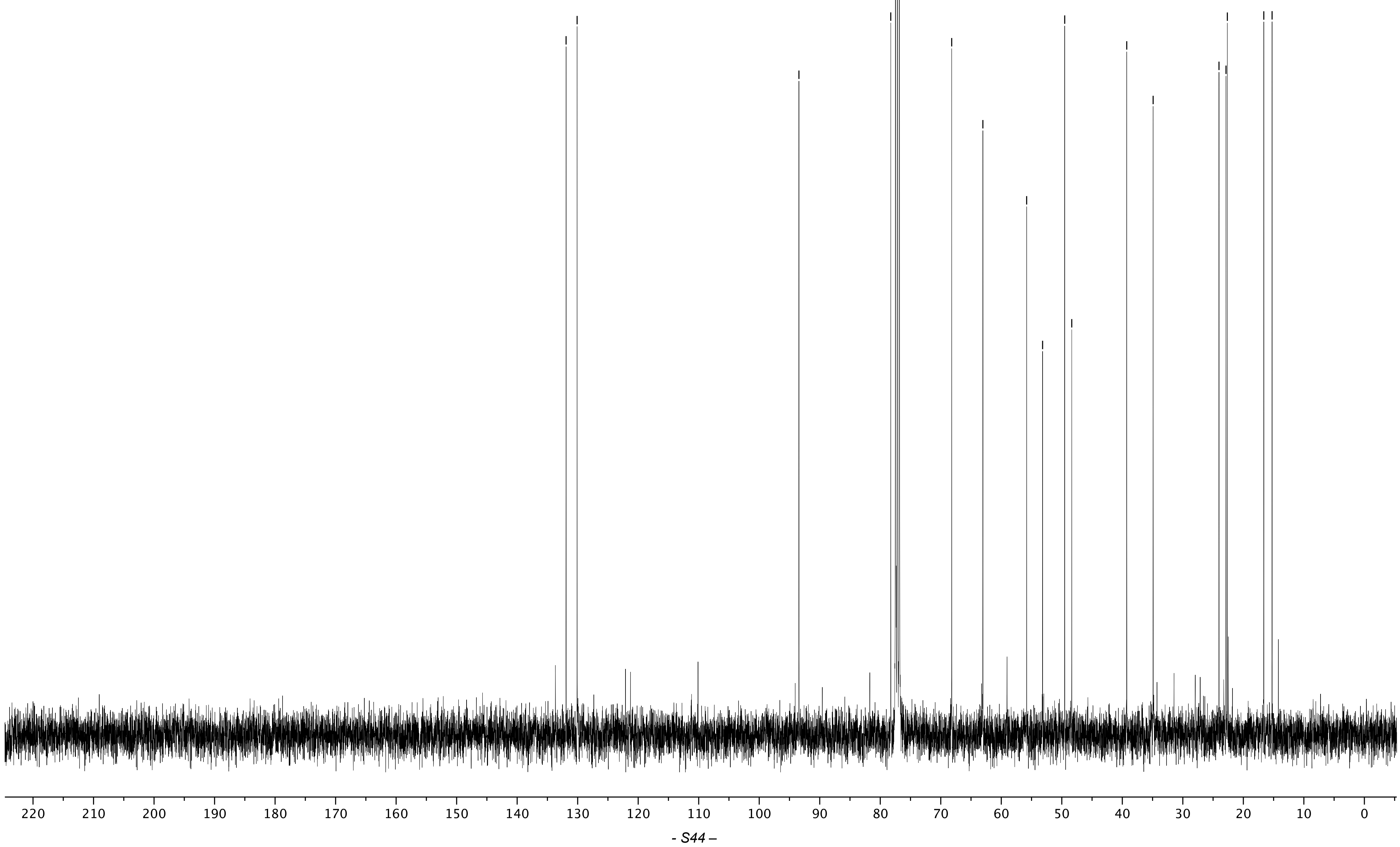

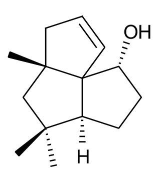

S3

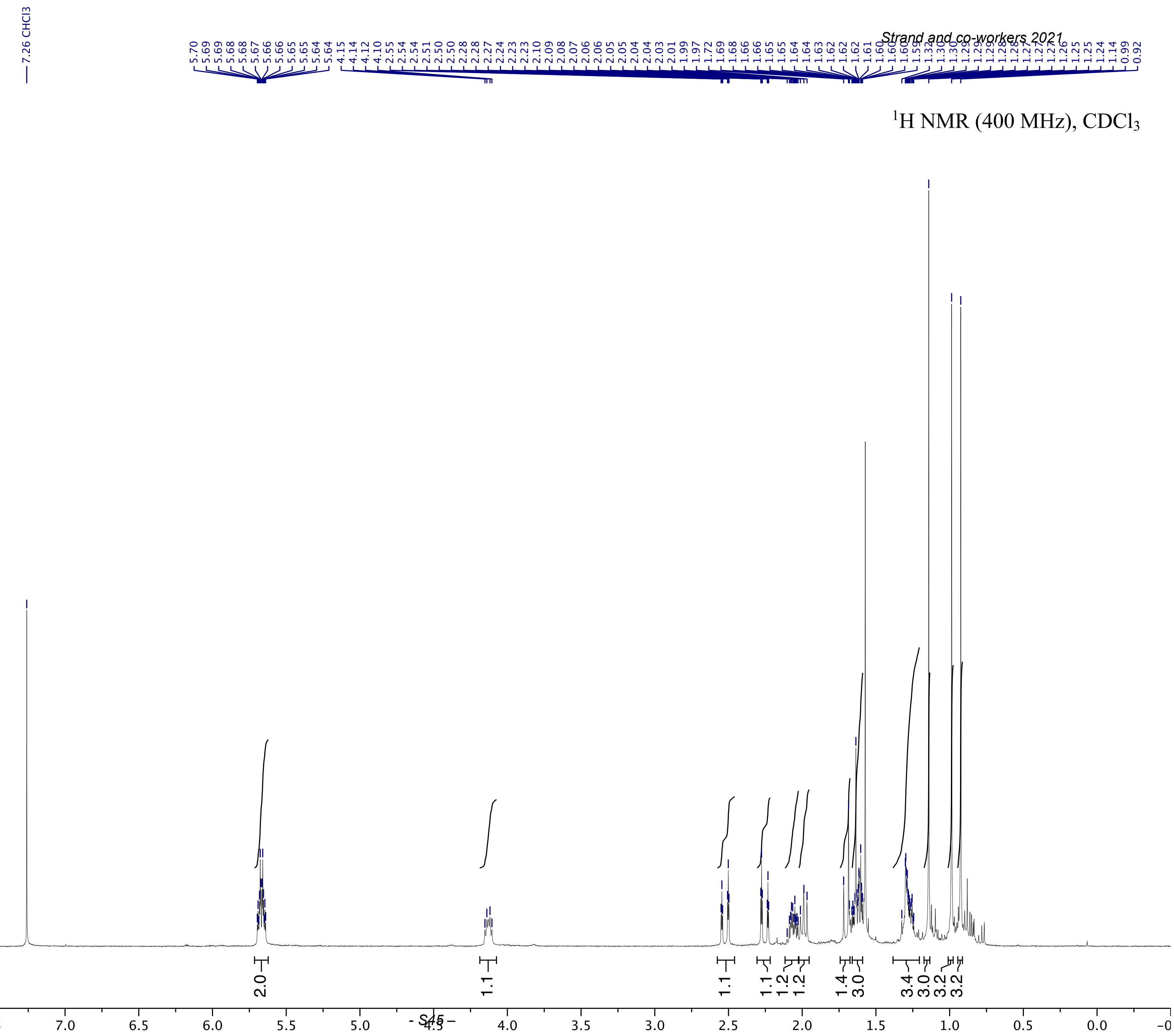

7.26 CHCl3
5.70 5.69 5.69 5.68 5.68 5.67 5.66 5.66 5.65 5.65 5.64 5.64
4.15 4.14 4.12 4.10 2.55 2.54 2.54 2.51 2.50 2.50 2.28 2.28 2.27 2.24 2.23 2.23 2.10 2.09 2.08 2.07 2.06 2.06 2.05 2.05 2.04 2.04 2.03 2.01 1.99 1.97 1.72 1.69 1.68 1.66 1.66 1.65 1.65 1.64 1.64 1.63 1.62 1.62 1.62 1.61 1.60 1.60 1.60 1.59 1.32 1.30 1.30 1.29 1.29 1.29 1.28 1.28 1.27 1.27 1.27 1.26 1.25 1.25 1.24 1.14 0.99 0.92
Strand and co-workers 2021
^{1}H NMR (400 MHz), $CDCl_3$
2.0
1.1
1.1
1.1
1.2
1.2
1.4
3.0
3.4
3.0
3.2
3.2
.5 9.0 8.5 8.0 7.5 7.0 6.5 6.0 5.5 5.0 4.5 4.0 3.5 3.0 2.5 2.0 1.5 1.0 0.5 0.0 -0

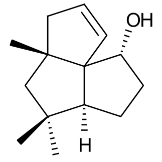

S3

Strand and co-workers 2021

^{13}C NMR (101 MHz), $CDCl_3$

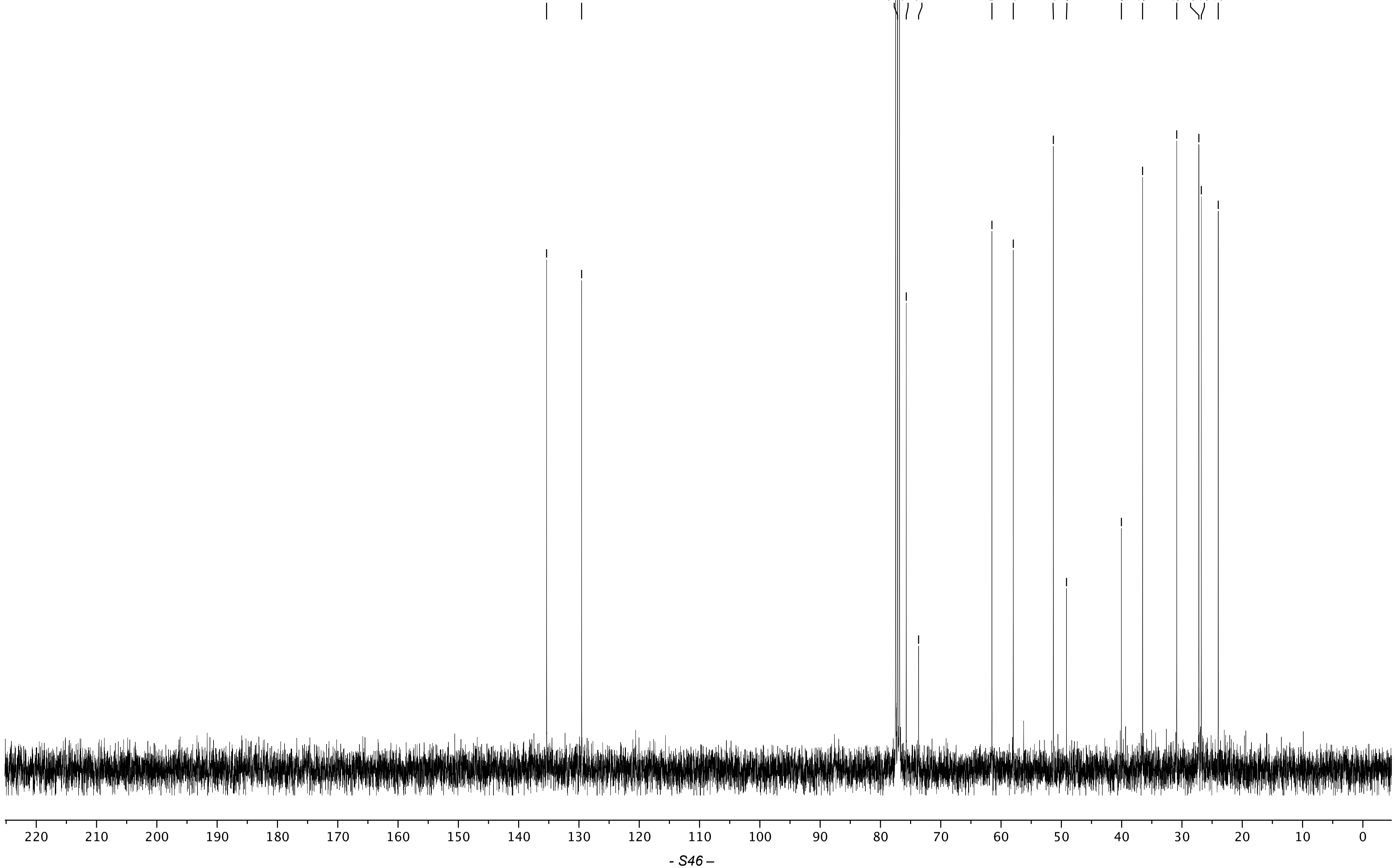

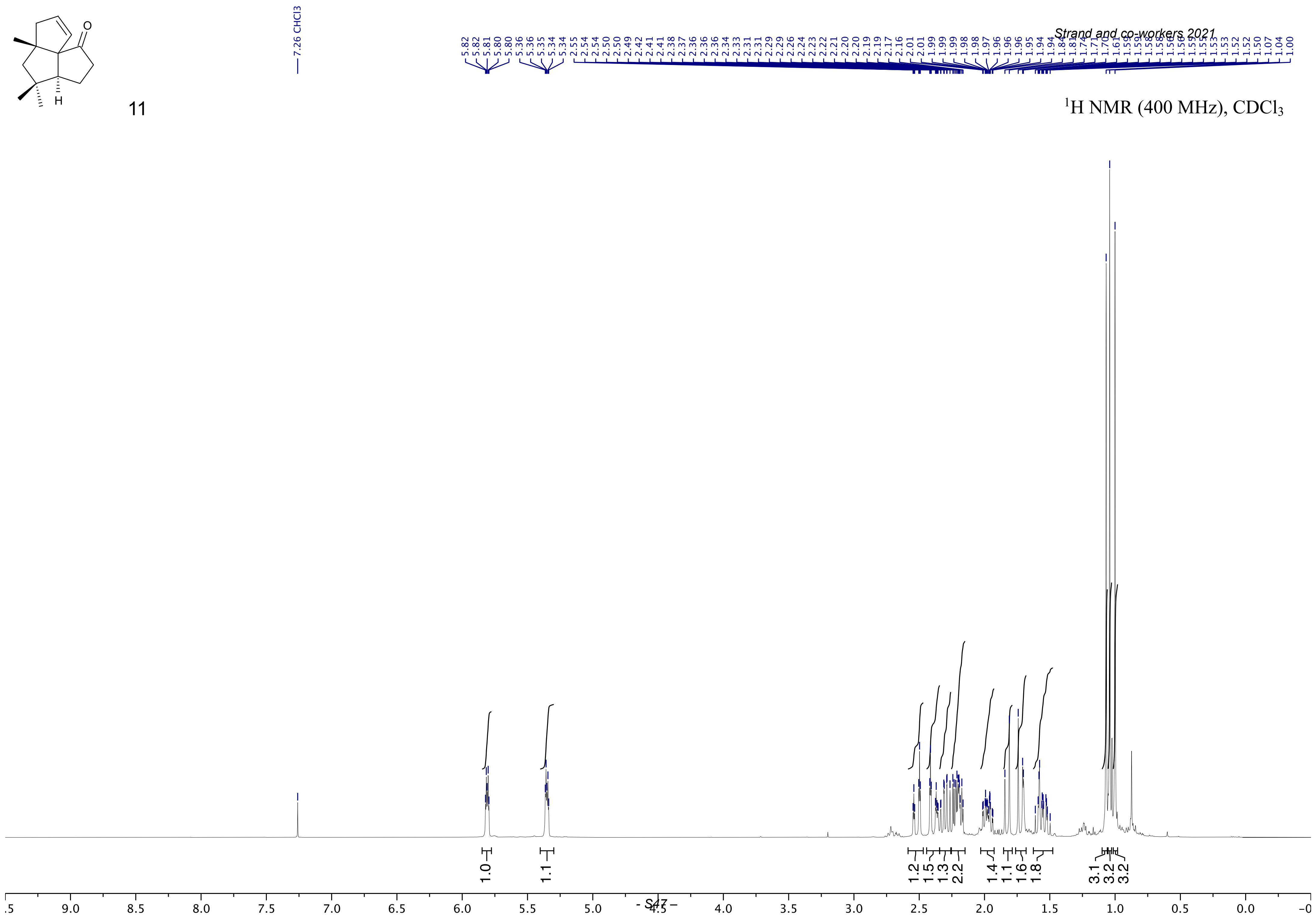

11
¹H NMR (400 MHz), CDCl3
Strand and co-workers 2021
7.26 CHCl3
1.0
1.1
1.2
1.5
1.3
2.2
1.4
1.1
1.6
1.8
3.1
3.2
3.2

Strand and co-workers 2021

^{13}C NMR (101 MHz), $CDCl_3$

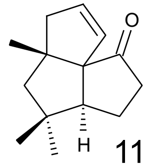

11

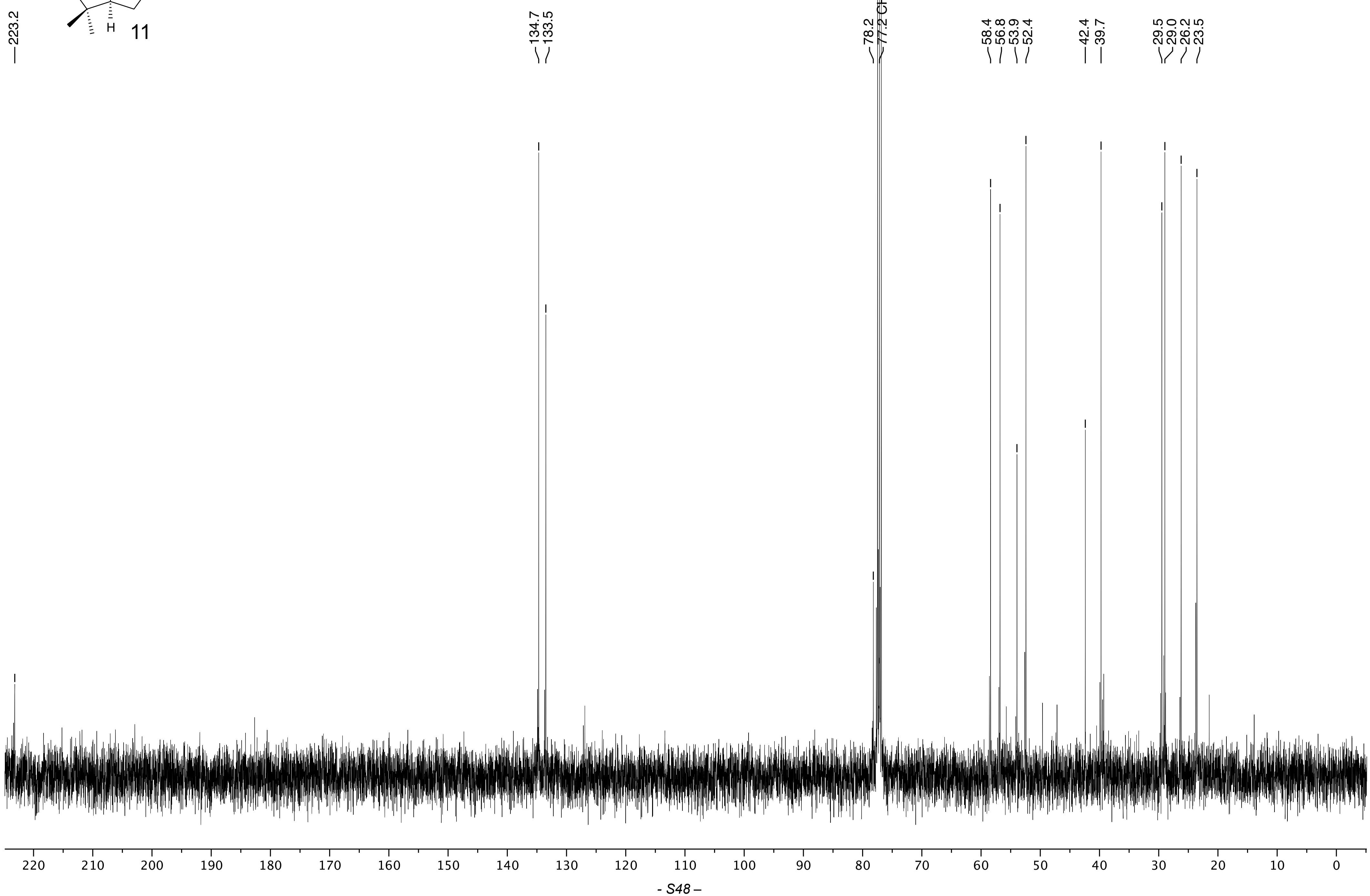

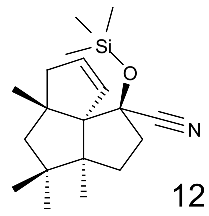

12

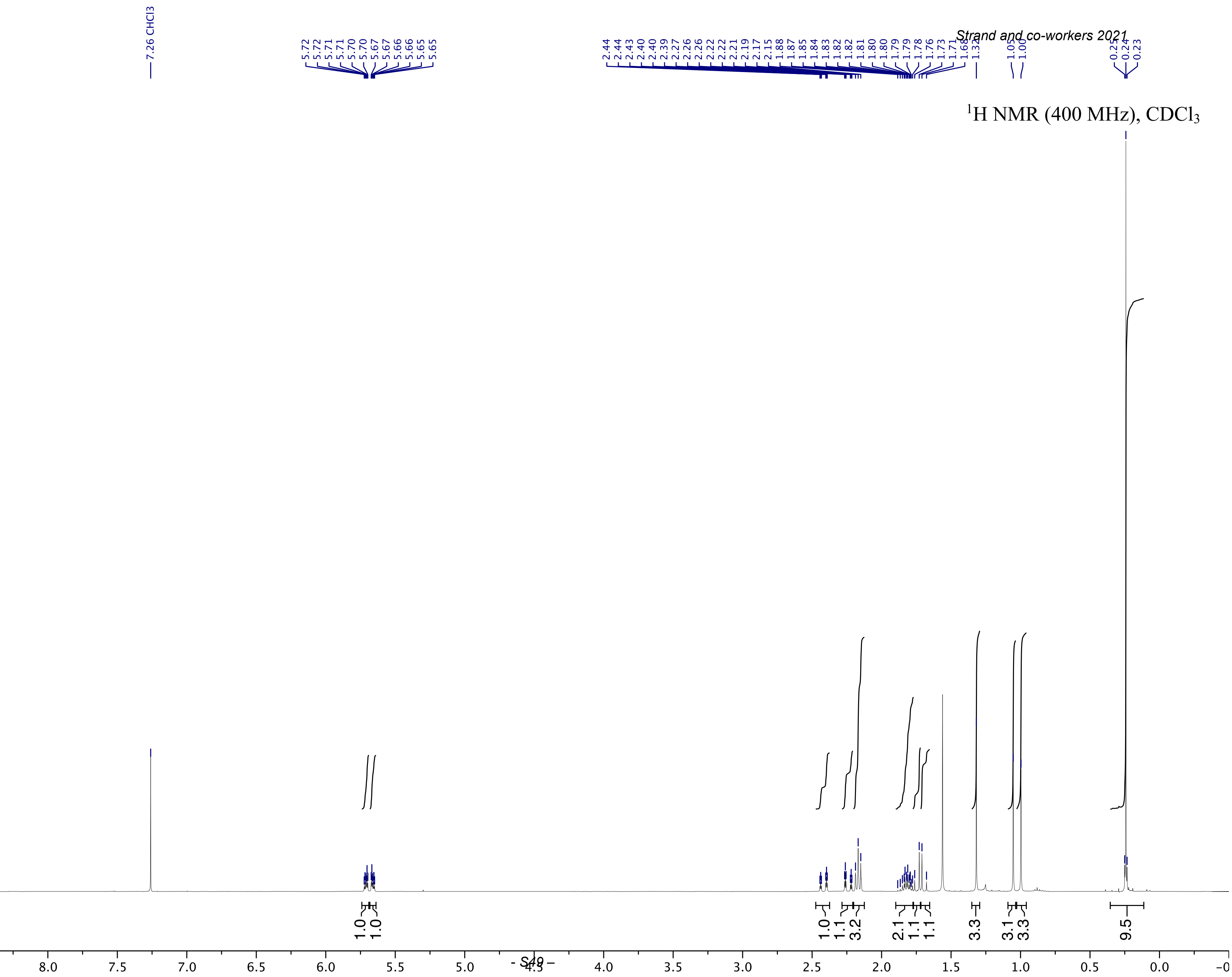

^{1}H NMR (400 MHz), $CDCl_3$

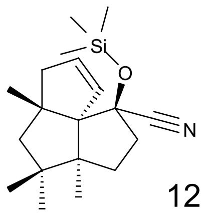

12

Strand and co-workers 2021

^{13}C NMR (101 MHz), $CDCl_3$

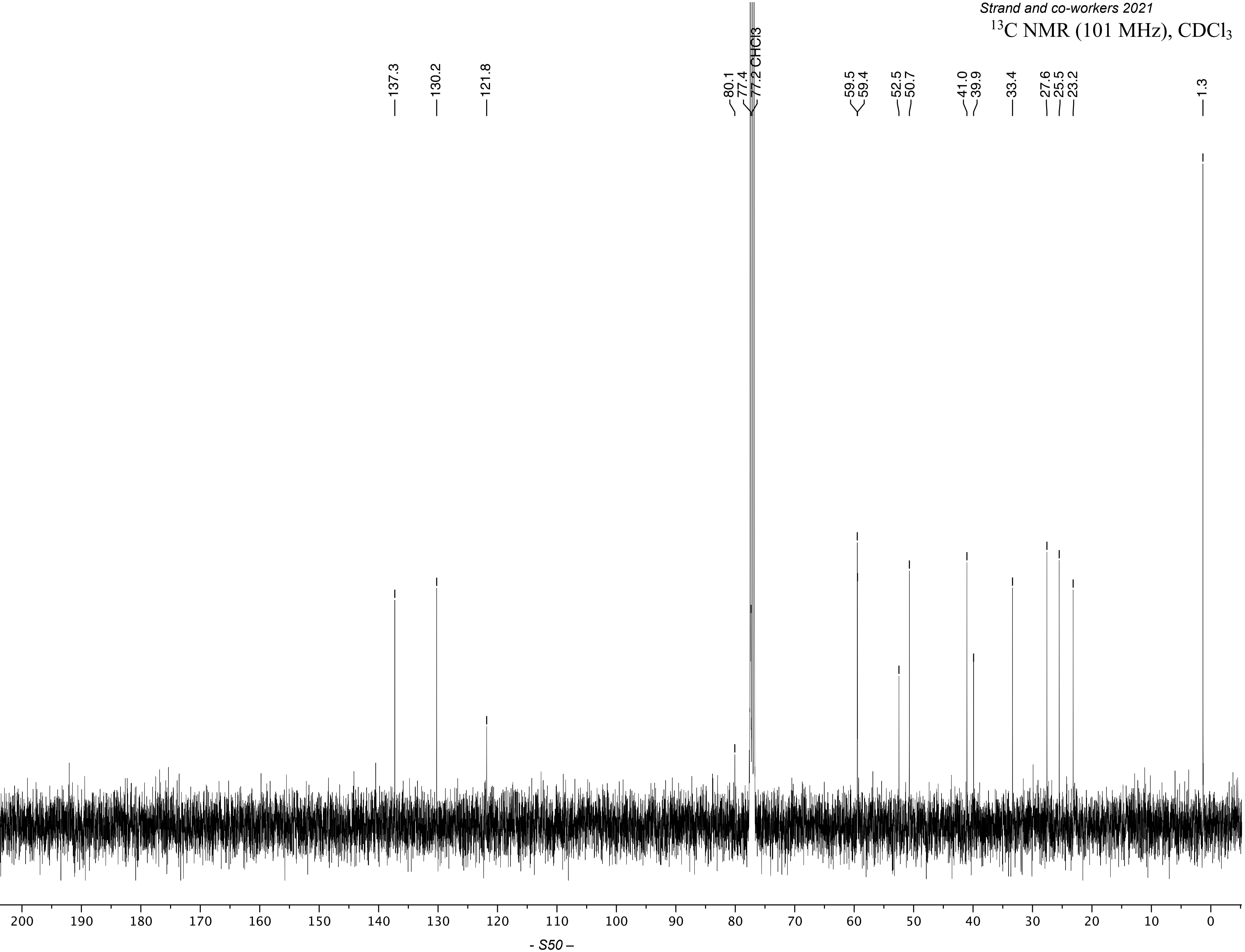

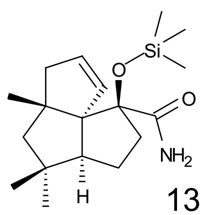
13

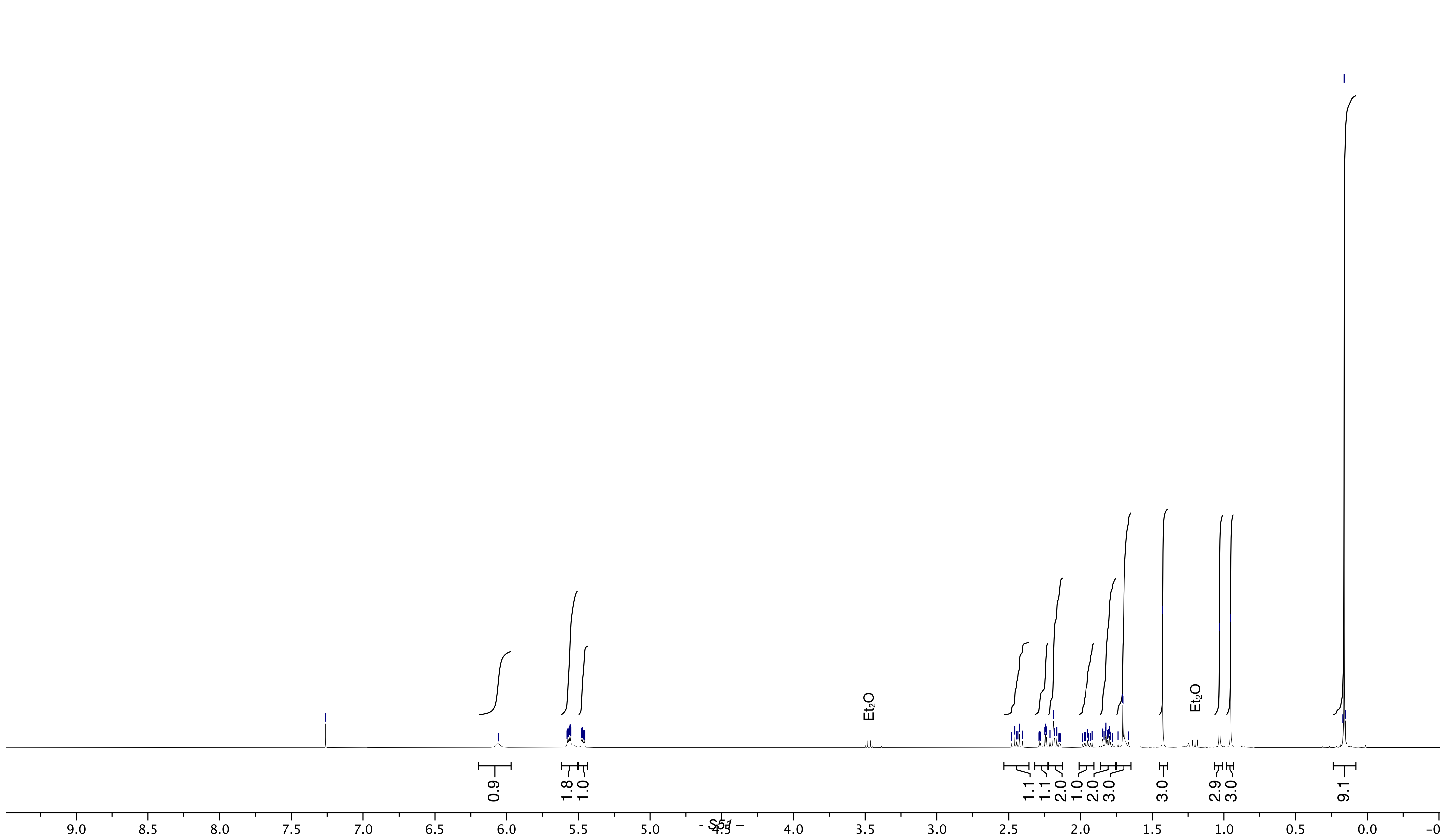

Strand and co-workers 2021
^1H NMR (400 MHz), $CDCl_3$
7.26 CHCl3
6.06 5.58 5.57 5.57 5.57 5.56 5.56 5.56 5.55 5.48 5.48 5.47 5.47 5.47 5.46 5.46 5.46
2.48 2.46 2.45 2.43 2.42 2.40 2.29 2.28 2.28 2.28 2.25 2.24 2.24 2.24 2.21 2.19 2.18 2.16 2.15 2.15 2.14 2.14 1.99 1.97 1.96 1.95 1.94 1.93 1.92 1.85 1.84 1.84 1.83 1.82 1.81 1.81 1.80 1.80 1.79 1.78 1.74 1.71 1.70 1.66
1.43
1.03 0.95
0.17 0.16 0.15
Et2O
Et2O
0.9
1.8
1.0
1.1
1.1
2.0
1.0
2.0
3.0
3.0
2.9
3.0
9.1
9.0 8.5 8.0 7.5 7.0 6.5 6.0 5.5 5.0 4.5 4.0 3.5 3.0 2.5 2.0 1.5 1.0 0.5 0.0 -0

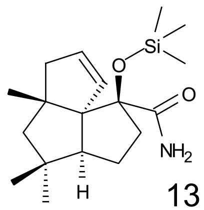

13

Strand and co-workers 2021

^{13}C NMR (101 MHz), $CDCl_3$

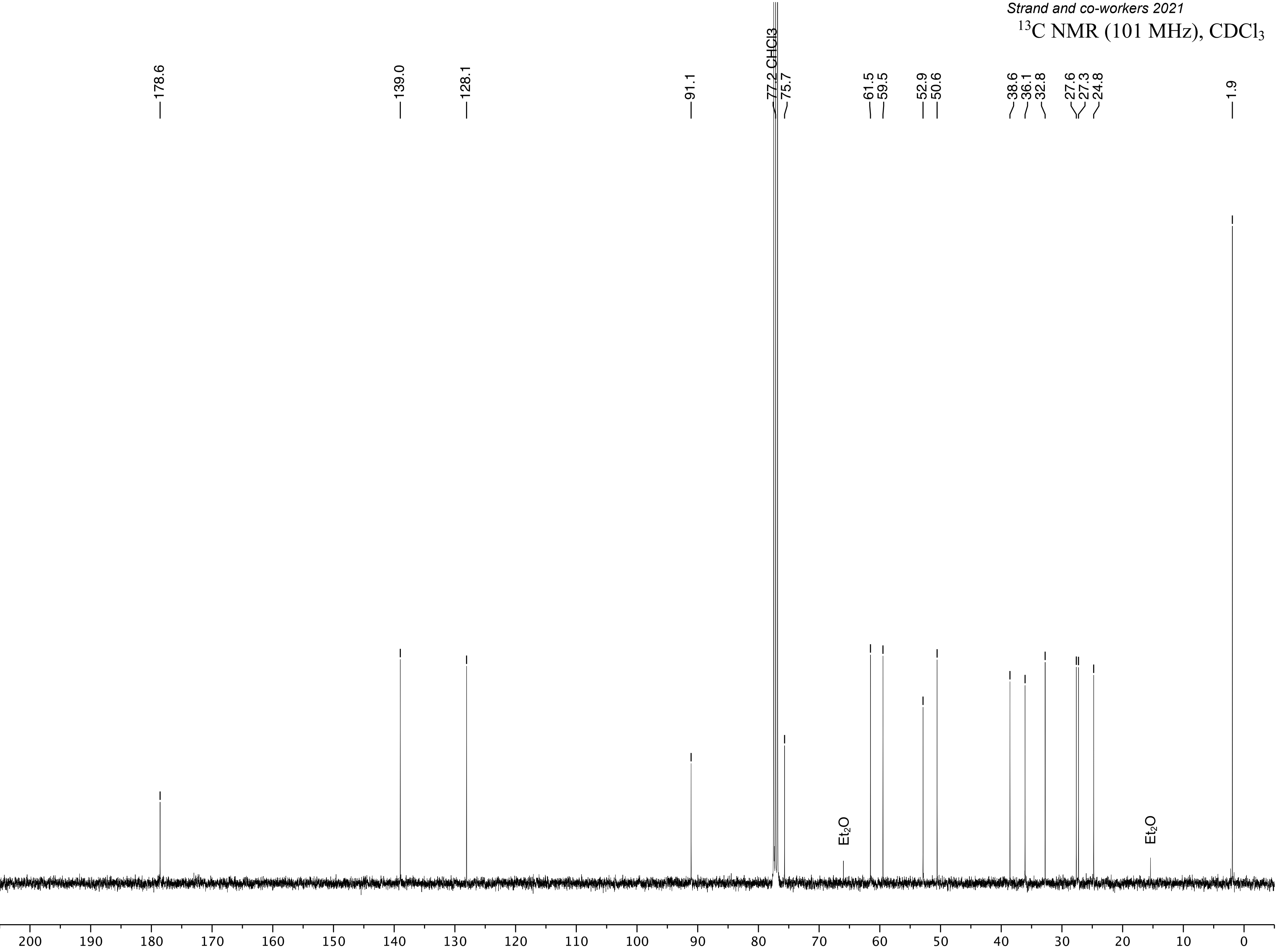

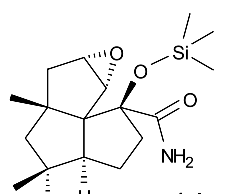

14

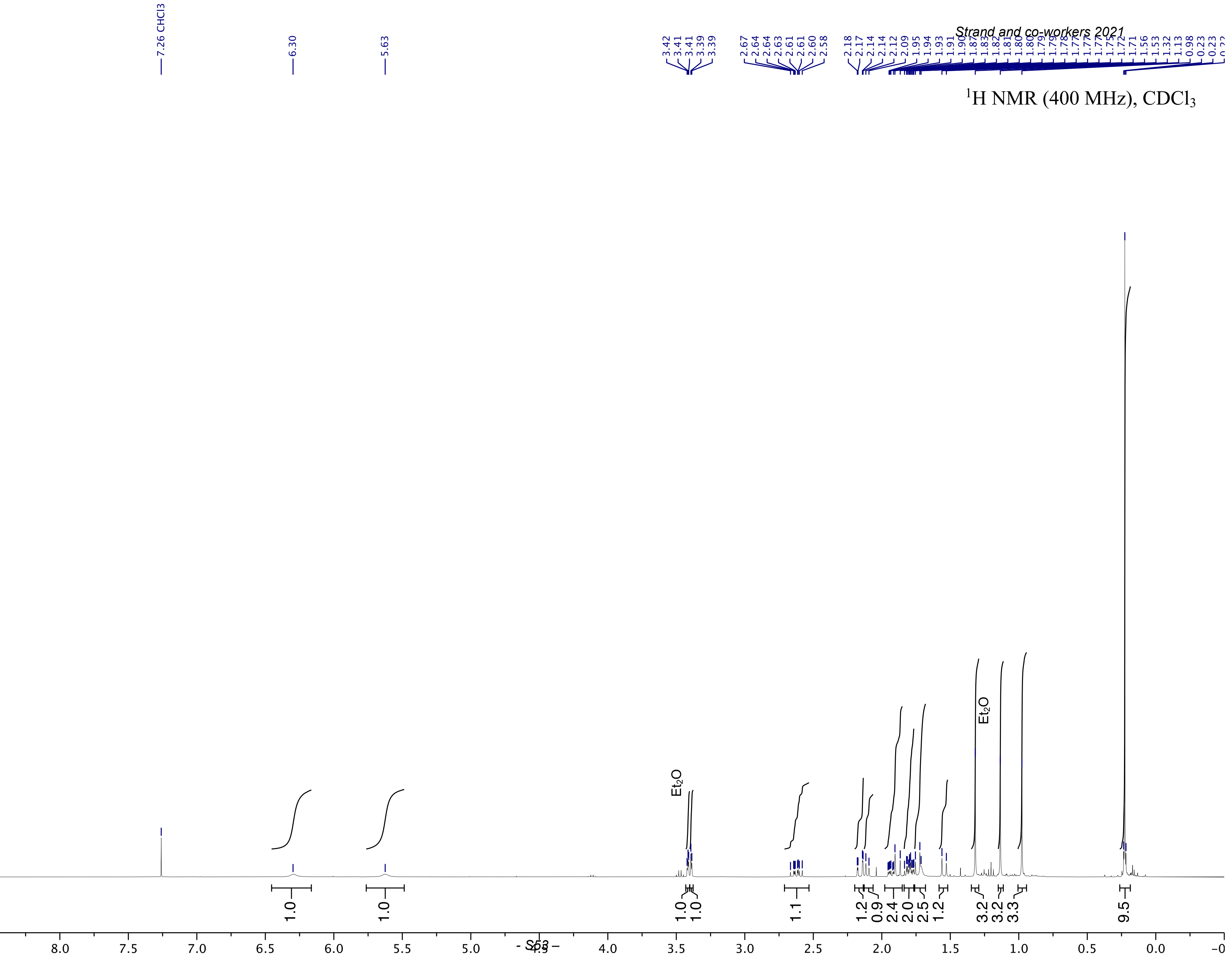

Strand and co-workers 2021
^{1}H NMR (400 MHz), $CDCl_3$
7.26 CHCl3
6.30
5.63
3.42
3.41
3.41
3.39
3.39
2.67
2.64
2.64
2.63
2.61
2.61
2.60
2.58
2.18
2.17
2.14
2.14
2.12
2.09
1.95
1.94
1.93
1.91
1.71
1.56
1.53
1.32
1.13
0.98
0.23
0.23
Et2O
Et2O
1.0
1.0
1.0
1.0
1.1
1.2
0.9
2.4
2.0
2.5
1.2
3.2
3.2
3.3
9.5
.5
9.0
8.5
8.0
7.5
7.0
6.5
6.0
5.5
5.0
4.0
3.5
3.0
2.5
2.0
1.5
1.0
0.5
0.0
–0

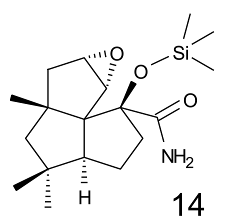

14

Strand and co-workers 2021

^{13}C NMR (101 MHz), $CDCl_3$

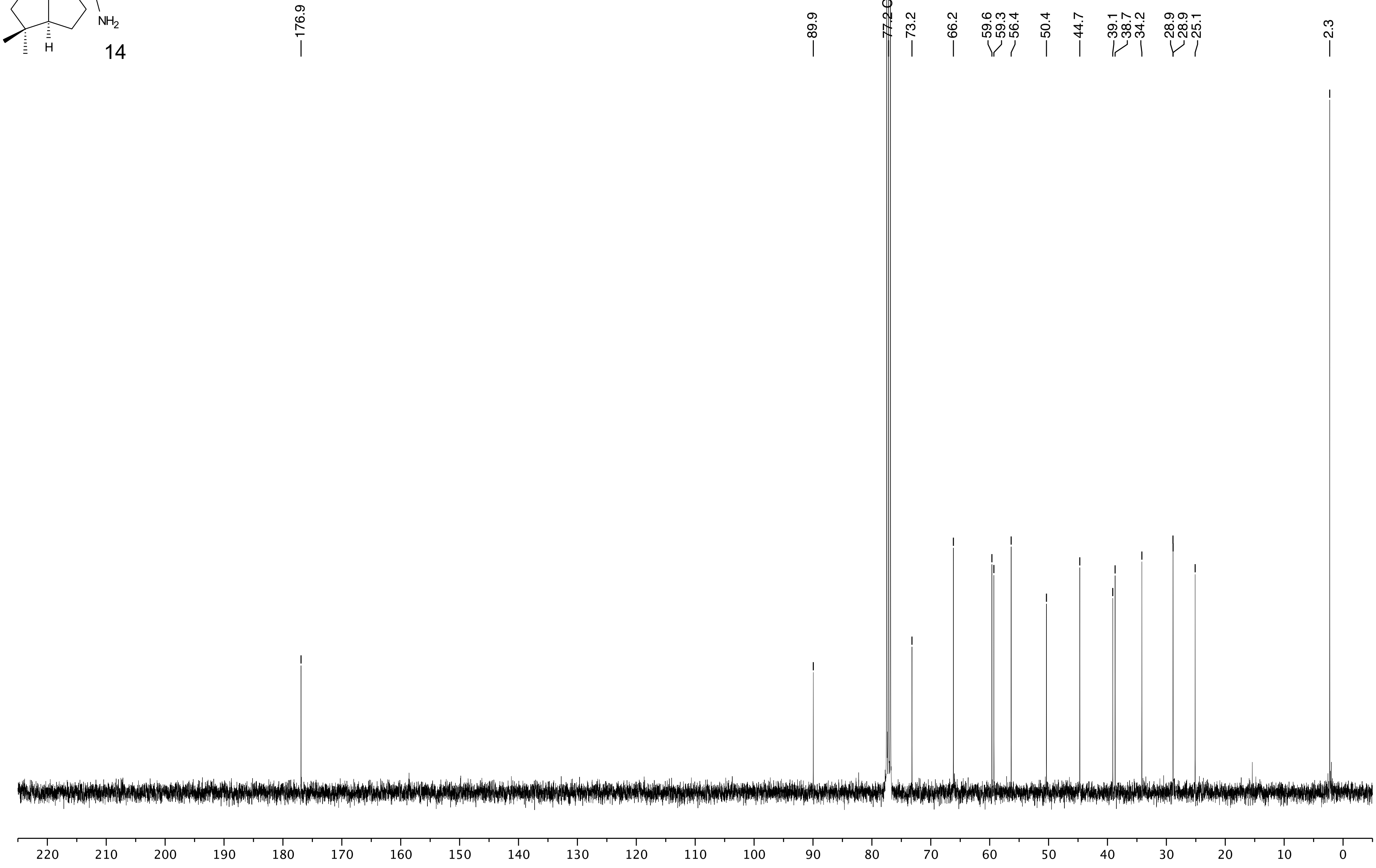

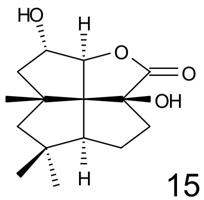

15

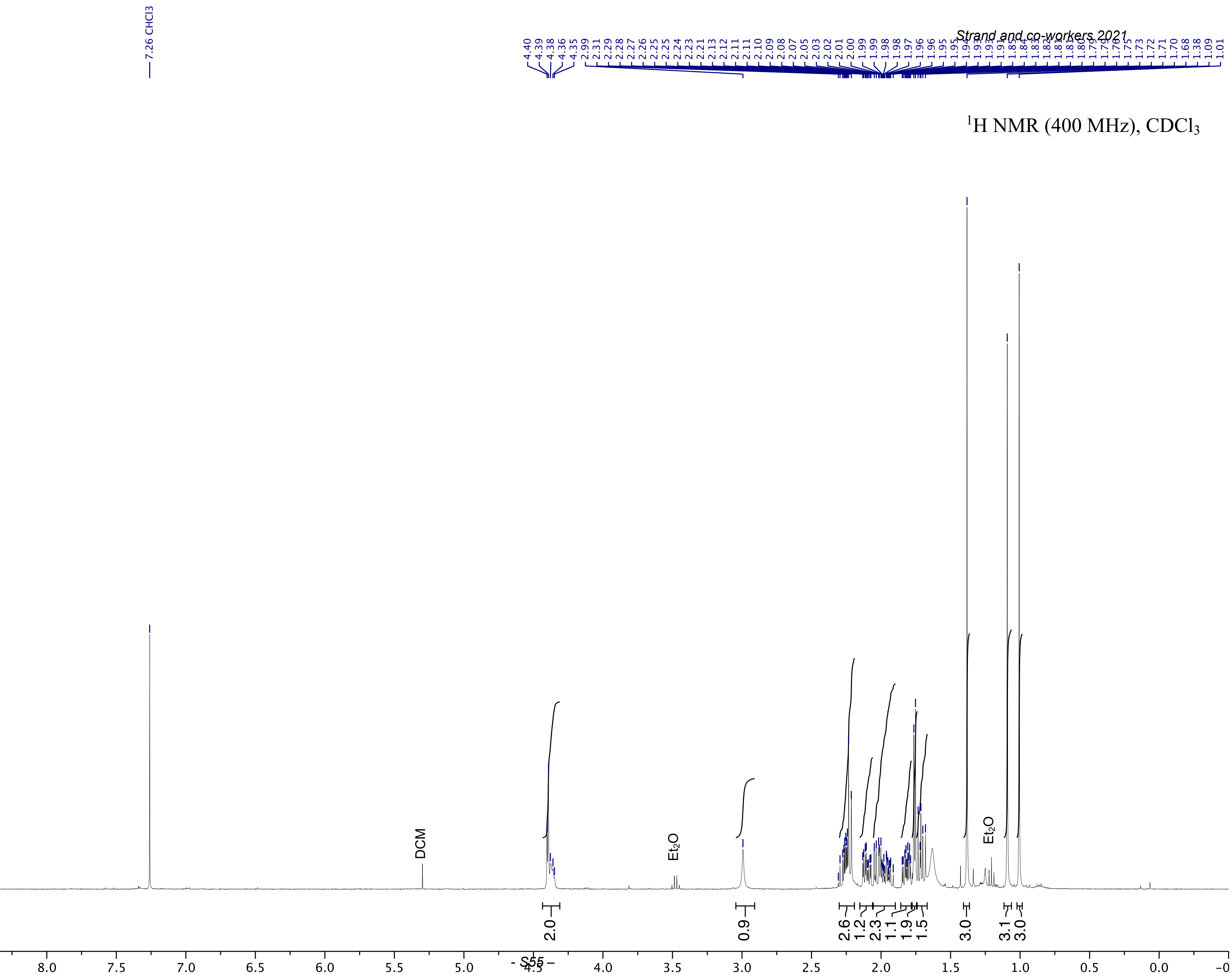

^1H NMR (400 MHz), $CDCl_3$

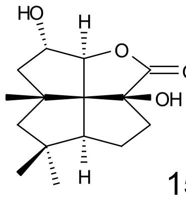

15

Strand and co-workers 2021

^{13}C NMR (101 MHz), $CDCl_3$

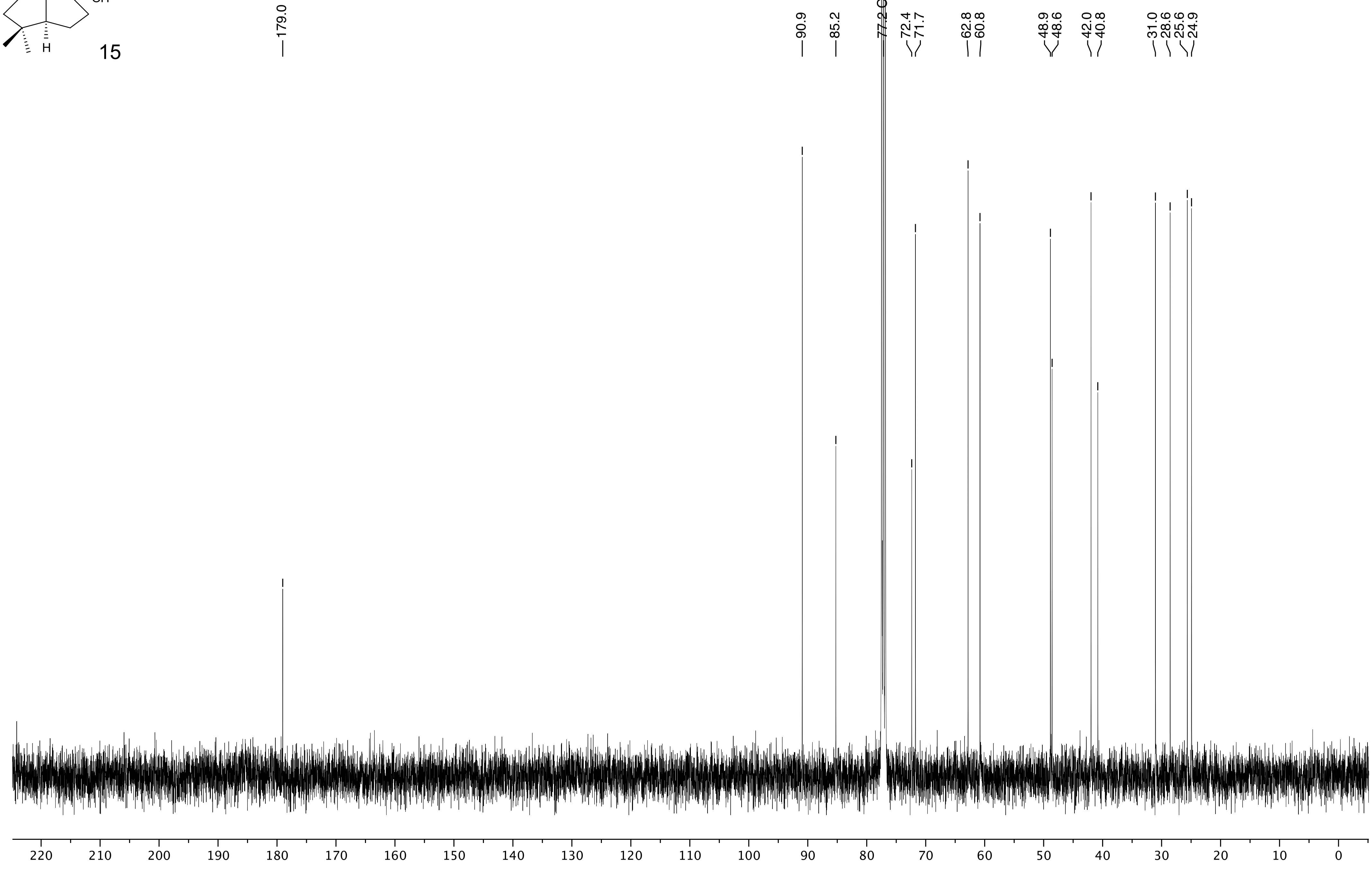

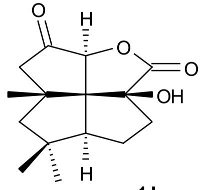

1b

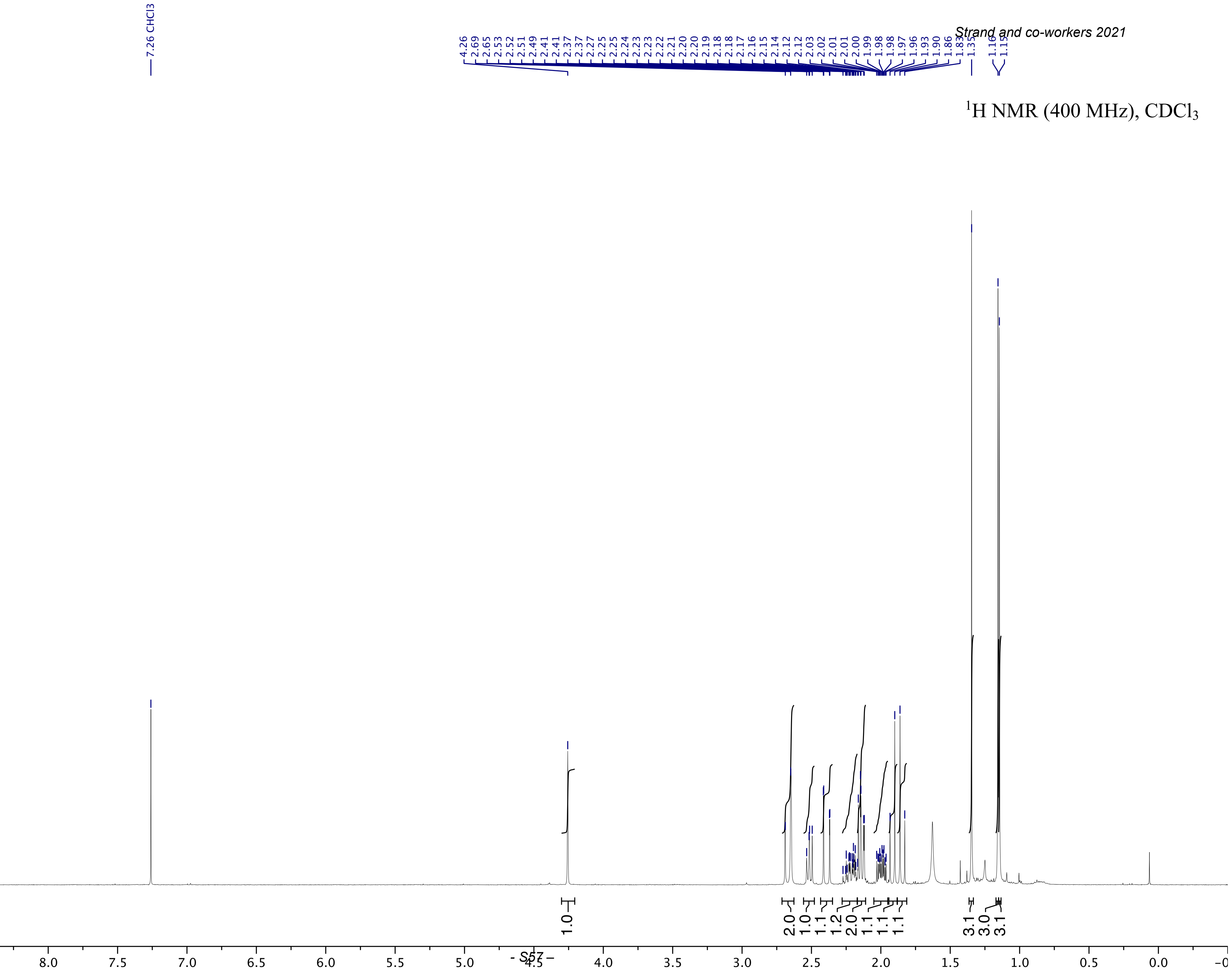

Strand and co-workers 2021

^{1}H NMR (400 MHz), $CDCl_3$

Strand and co-workers 2021

^{13}C NMR (101 MHz), $CDCl_3$

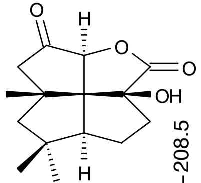

1b

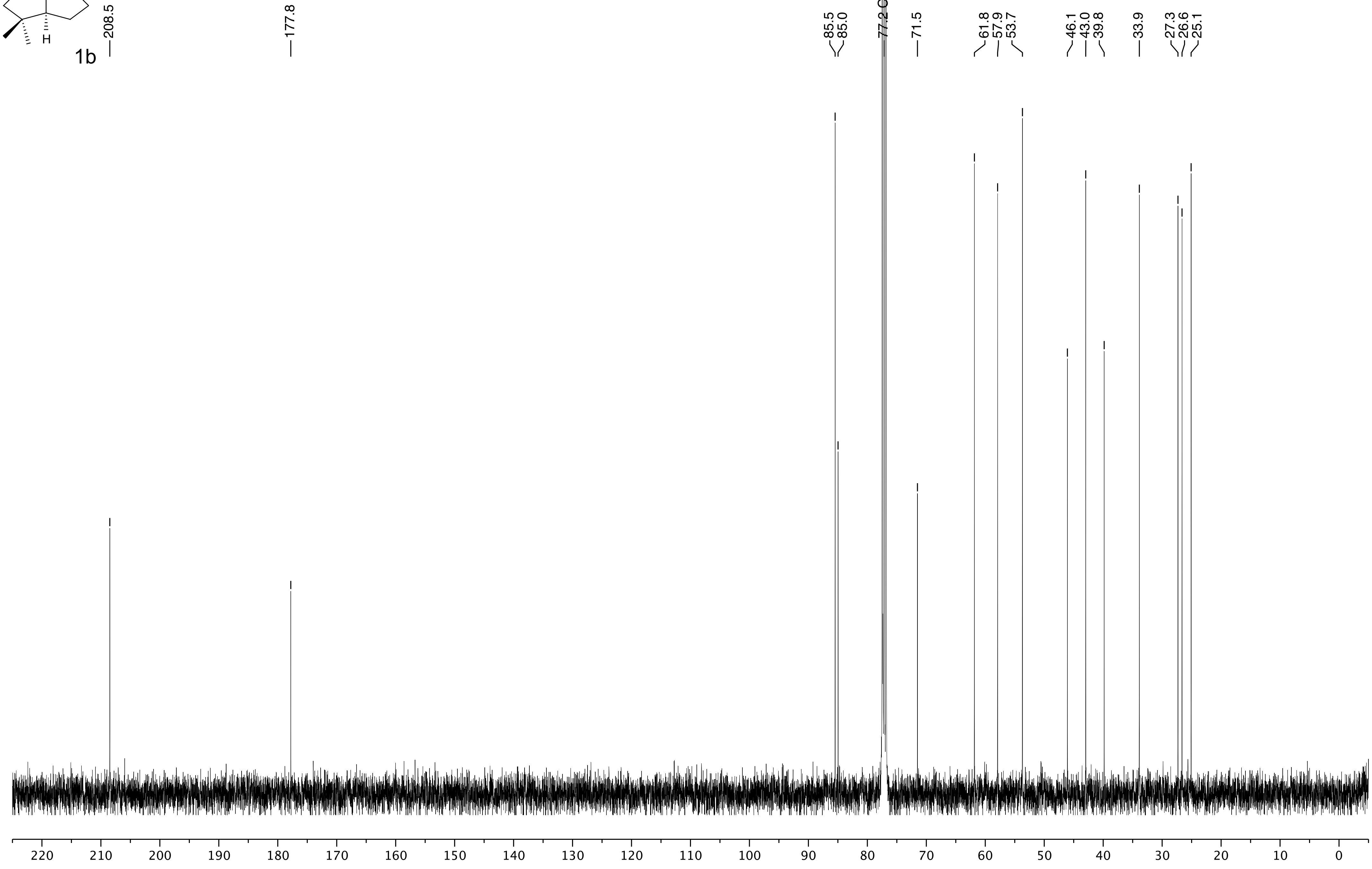

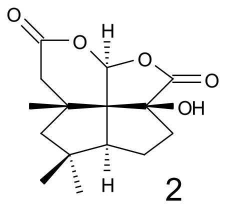

2

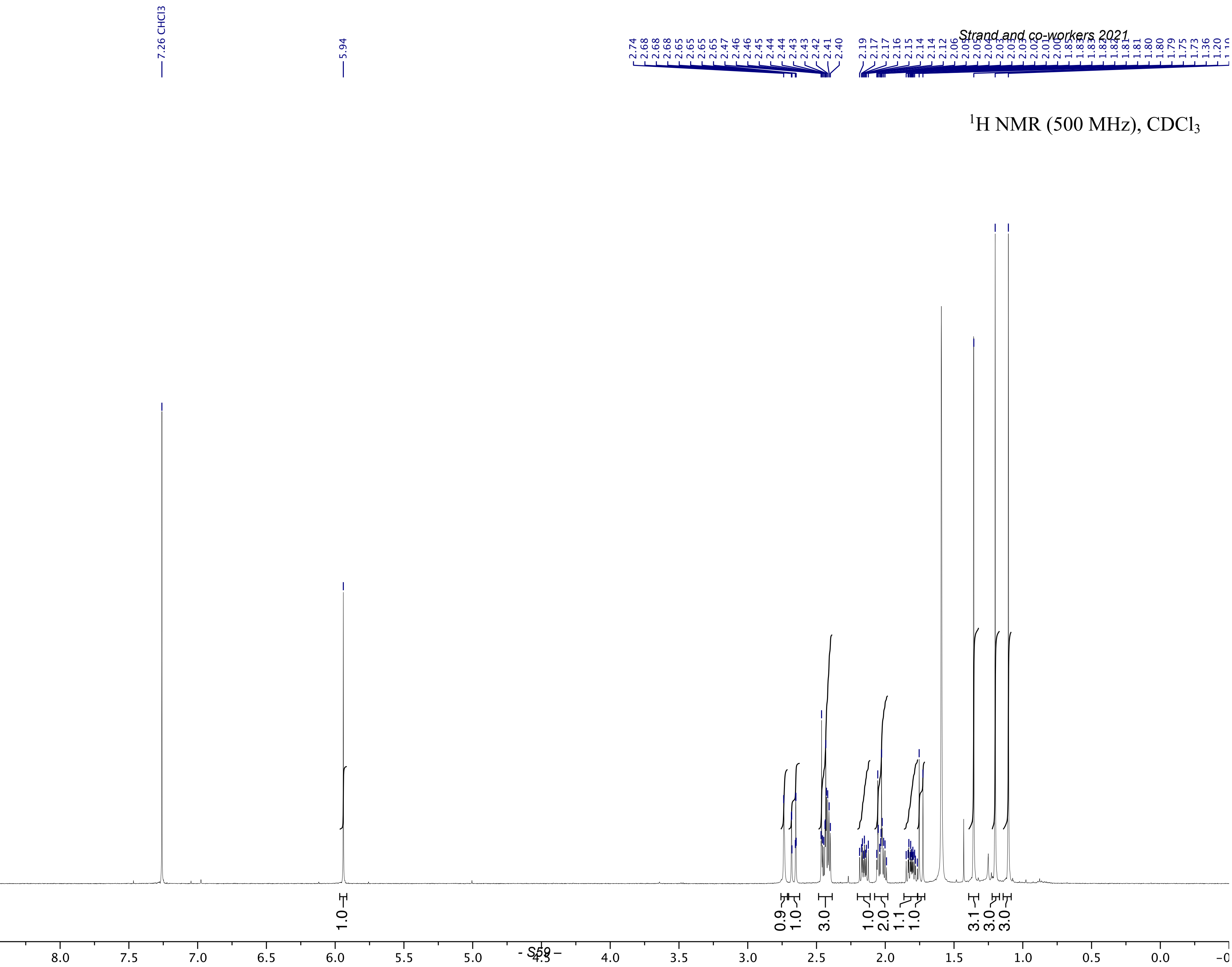

^1H NMR (500 MHz), $CDCl_3$

Strand and co-workers 2021

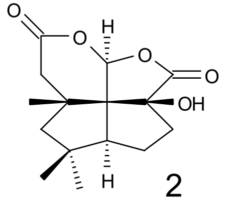

2

Strand and co-workers 2021

^{13}C NMR (126 MHz), $CDCl_3$

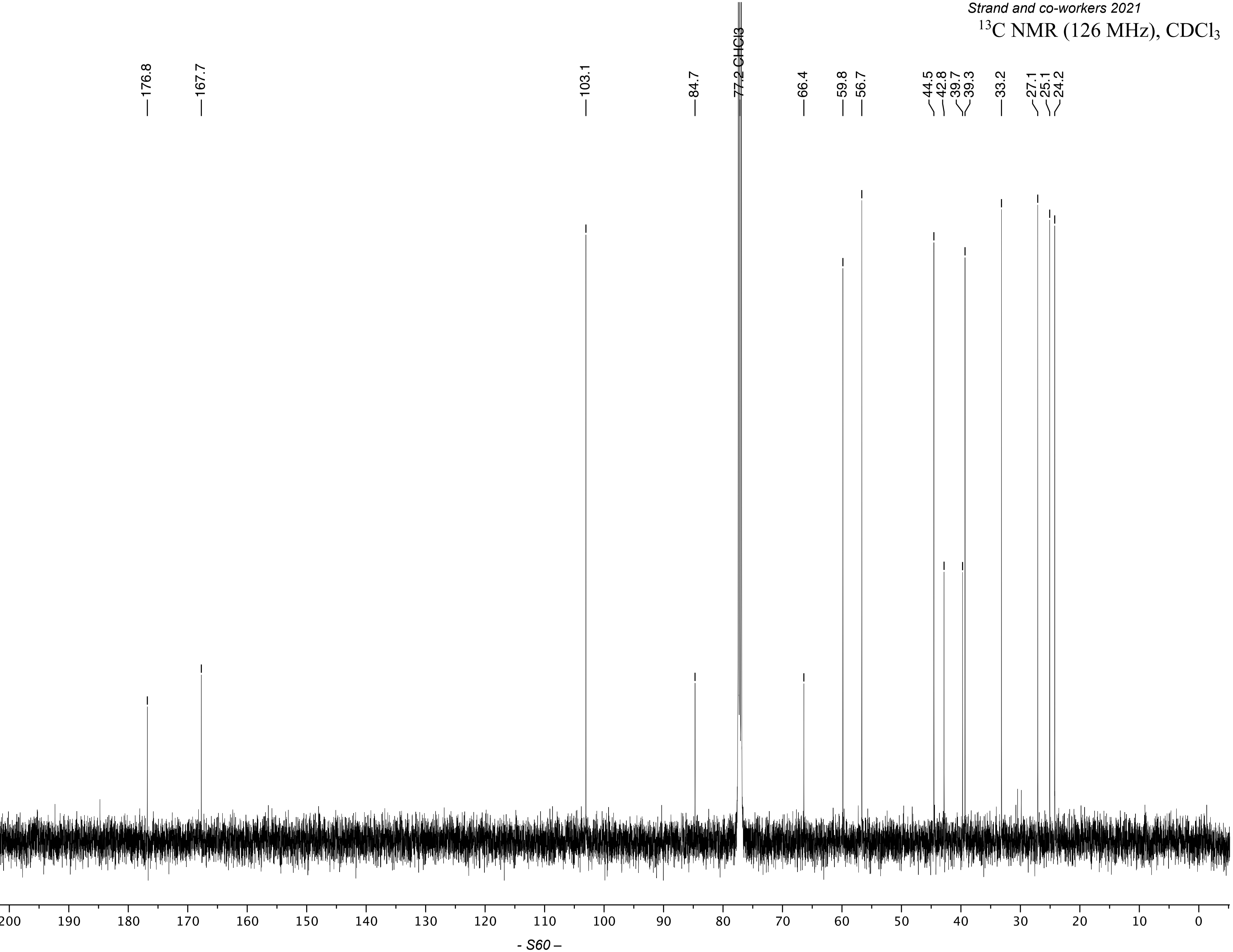

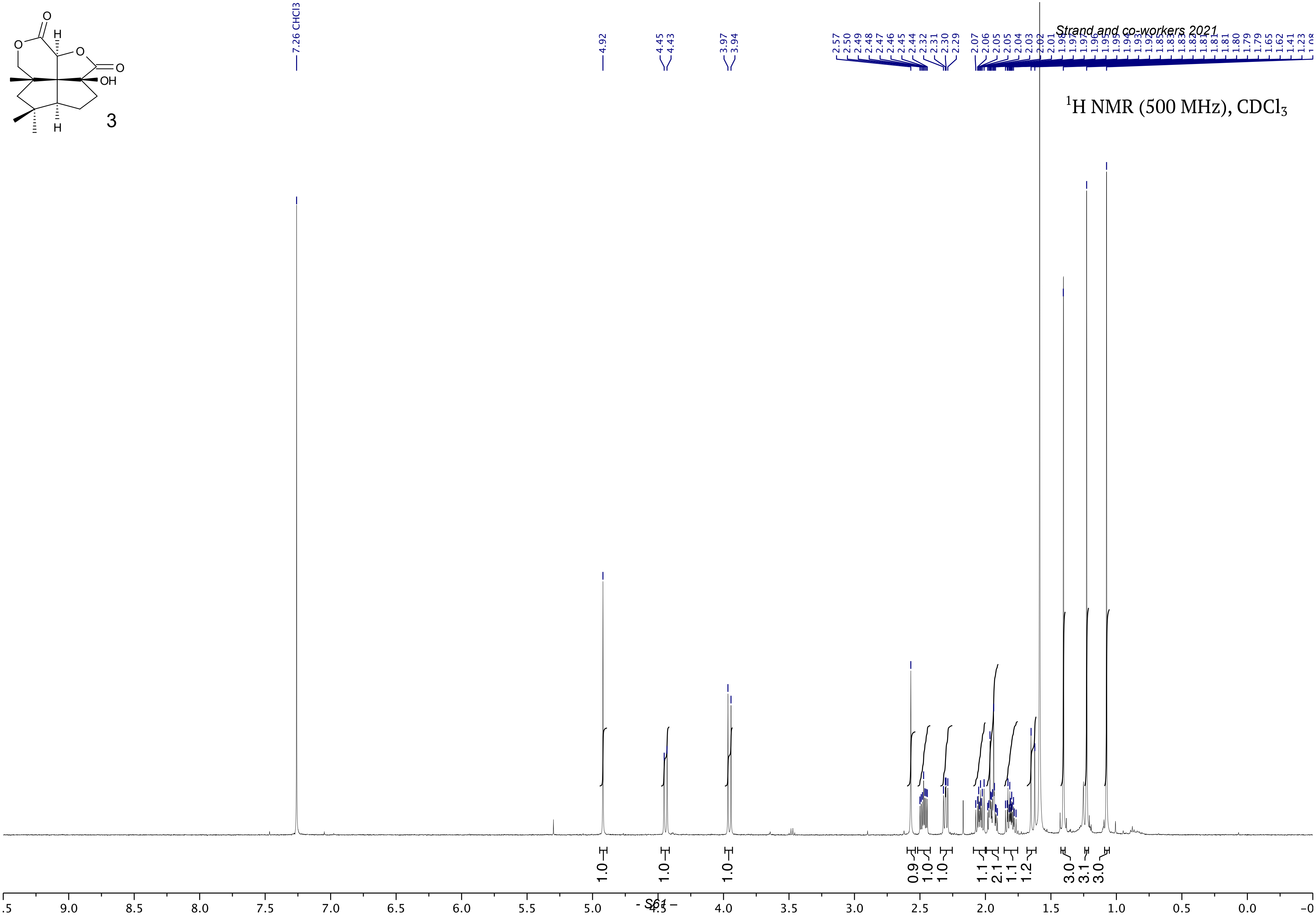

3
Strand and co-workers 2021
^{1}H NMR (500 MHz), $CDCl_3$
7.26 CHCl3
4.92
4.45
4.43
3.97
3.94
1.0
1.0
1.0
0.9
1.0
1.0
1.1
2.1
1.1
1.2
3.0
3.1
3.0

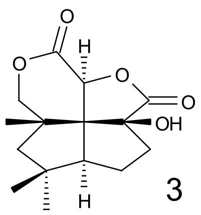

3

Strand and co-workers 2021

^{13}C NMR (126 MHz), $CDCl_3$

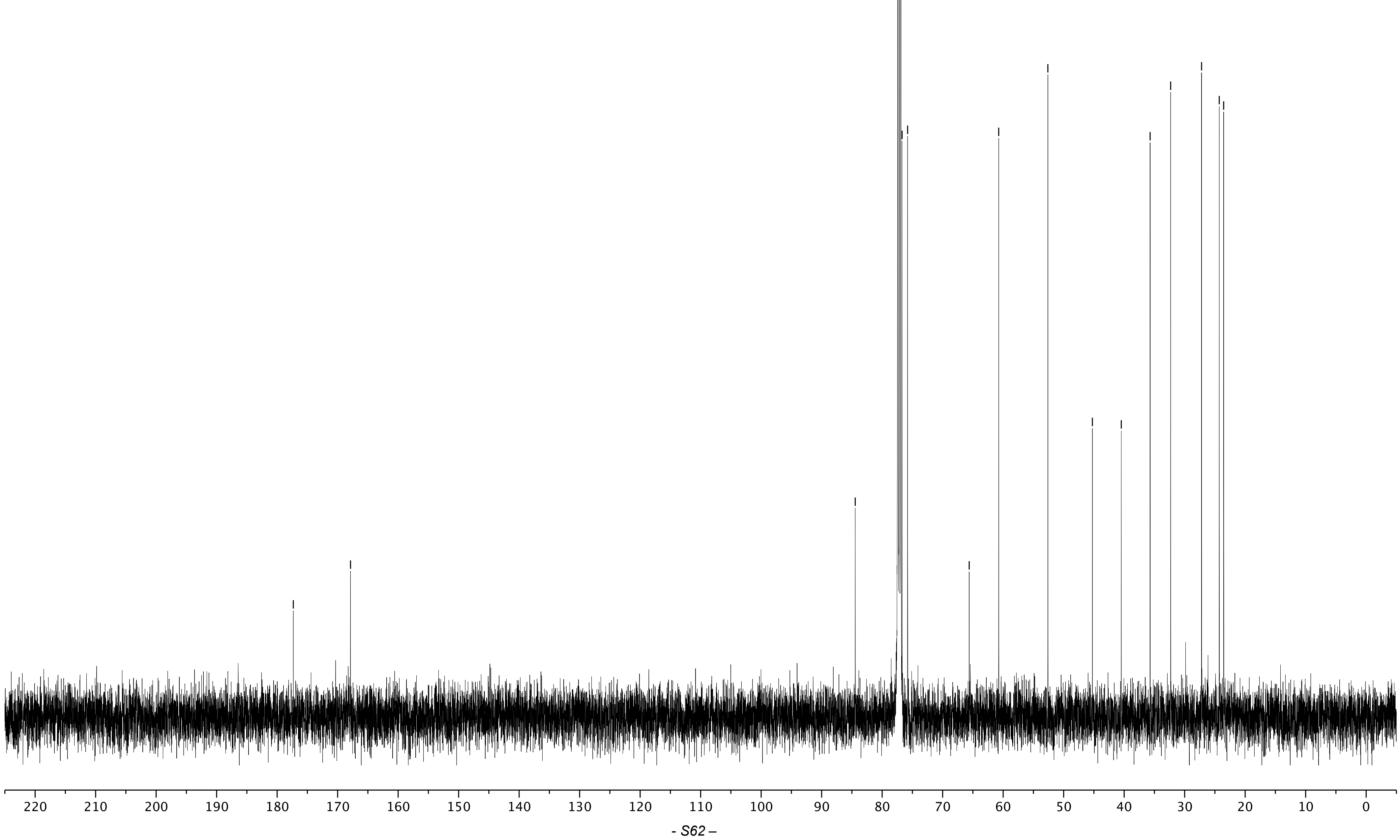

Supplement: Supplementary file 1 — ol1c00955_si_001.pdf [file ol1c00955_si_001.pdf]
